# Highly Conjugated π‐Systems Arising from Cannibalistic Hexadehydro‐Diels–Alder Couplings: Cleavage of C−C Single and Triple Bonds

**DOI:** 10.1002/chem.202002511

**Published:** 2020-10-29

**Authors:** Jan Maier, Marian Deutsch, Julia Merz, Qing Ye, Oliver Diamond, Maja‐Tessa Schilling, Alexandra Friedrich, Bernd Engels, Todd B. Marder

**Affiliations:** ^1^ Institut für Anorganische Chemie and Institute for Sustainable Chemistry &, Catalysis with Boron (ICB) Julius-Maximilians-Universität Würzburg Am Hubland 97074 Würzburg Germany; ^2^ Institut für Physikalische und Theoretische Chemie, Julius-Maximilians-Universität Würzburg Emil-Fischer-Straße 42 97074 Würzburg Germany; ^3^ Southern University of Science and Technology No 1088, Xueyuan Rd. Xili, Nanshan District Shenzhen, Guangdong P. R. China

**Keywords:** alkyne, aryne, biradical, C−C activation, hexadehydro-Diels–Alder reaction

## Abstract

We have investigated the cannibalistic self‐trapping reaction of an *ortho*‐benzyne derivative generated from 1,11‐bis(*p*‐tolyl)undeca‐1,3,8,10‐tetrayne in an HDDA reaction. Without adding any specific trapping agent, the highly reactive benzyne is trapped by another bisdiyne molecule in at least three different modes. We have isolated and characterized the resulting products and performed high‐level calculations concerning the reaction mechanism. During the cannibalistic self‐trapping process, either a C≡C triple bond or an sp–sp^3^ C−C single bond is cleaved. Up to seven rings and nine C−C bonds are formed starting from two 1,11‐bis(*p*‐tolyl)undeca‐1,3,8,10‐tetrayne molecules. Our experiments and calculations provide considerable insight into the variety of reaction pathways which the *ortho*‐benzyne derivative, generated from a bisdiyne, can take when reacting with another bisdiyne molecule.

## Graphical abstract

**Go for the throat**: The benzyne derivative generated from a linked bisdiyne in a hexadehydro‐Diels–Alder reaction undergoes cannibalistic self‐trapping by at least three different pathways. Three highly conjugated products resulting from self‐trapping were isolated, fully characterized and their photophysical properties examined. The mechanisms leading to their formation were investigated by high‐level calculations.

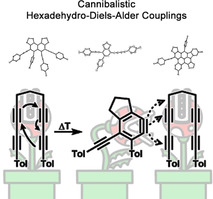

## Introduction

**Figure 1:**
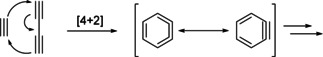
Hexadehydro‐Diels–Alder (HDDA) reaction.

Cyclization reactions have long been a way of generating structures of high complexity in an elegant fashion. For almost 90 years, the [4+2]‐addition, discovered by Diels and Alder, has been known and applied countless times.^[1]^ An evolution thereof is the so‐called hexadehydro‐Diels–Alder (HDDA) reaction, which results in highly reactive benzyne intermediates (Figure 1).^[2]^

**Figure 2:**
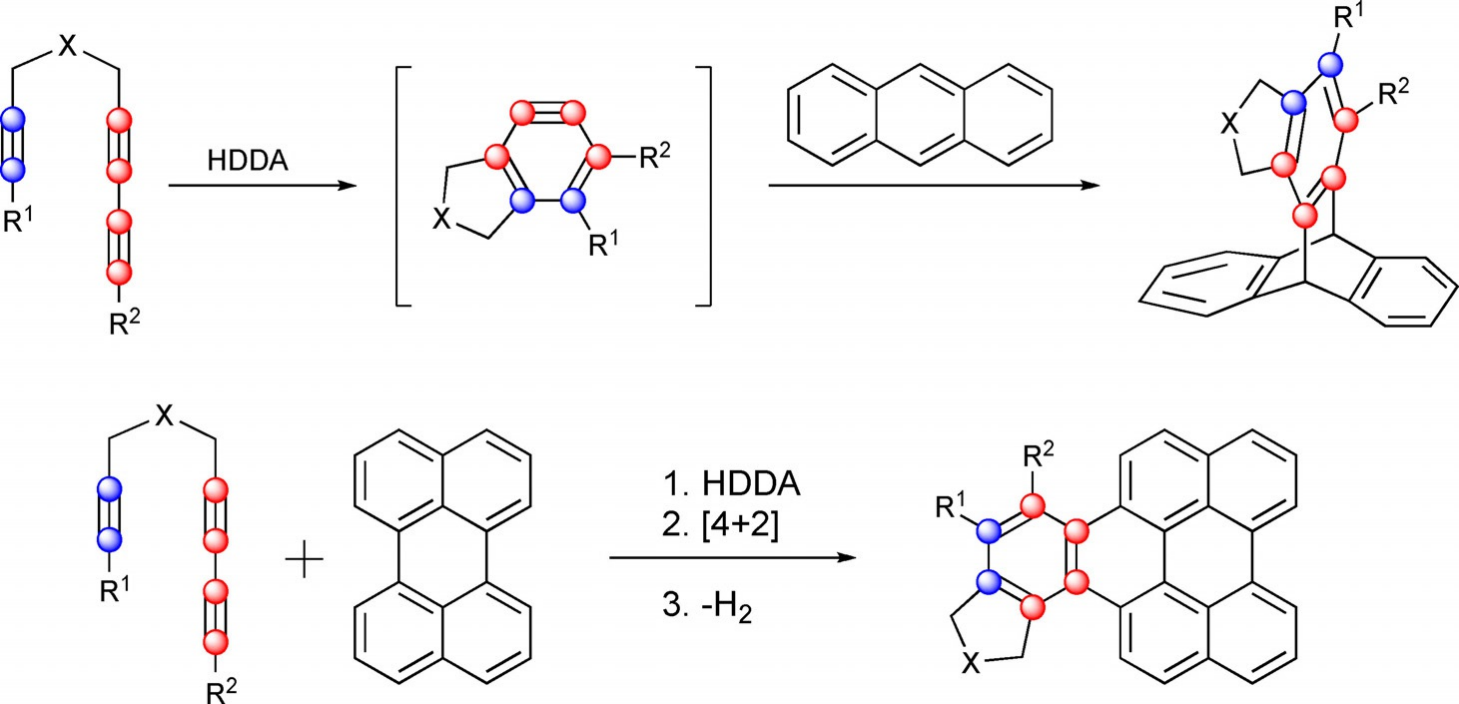
General trapping of HDDA‐generated benzyne with anthracene (top) and perylene (bottom).

This type of reaction was observed by Johnson^[3]^ and Ueda^[4]^ in 1997, and was further developed by Hoye and co‐workers starting in 2012, by the use of linked tri‐ and tetraynes.^[5]^ An established reagent for the intermolecular trapping of benzynes is anthracene, which gives the corresponding triptycene derivative in a Diels–Alder reaction (Figure 2, top).[^4^, ^6^] Furthermore, Hoye et al. reported a very interesting reaction of HDDA‐generated benzynes with perylenes in 2016.^[7]^ In this reaction, as in the trapping with anthracene, a [4+2]‐cycloaddition takes place. The elimination of dihydrogen, in order to re‐aromatize the perylene moiety, represents the final step in the proposed reaction mechanism (Figure 2, bottom).

**Figure 3:**
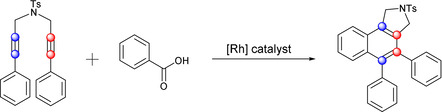
Rhodium‐promoted [2+1+2+1]‐cycloaddition of a 1,6‐diyne with benzoic acid.

A variety of follow‐up reactions to the HDDA process have been investigated, ranging from cleverly designed intramolecular reactions^[2]^ to external trapping with reagents ranging from furan^[8]^ to reactions with complex natural products.^[9]^ This versatility makes the HDDA reaction especially valuable. Bond activations include, among others, C−O, C−Si, Si−O and C−H.^[10]^ However, the cleavage of C−C bonds, especially of triple bonds, which is still one of the most challenging reactions in organic chemistry, has been reported only rarely with benzyne intermediates.^[11]^ Recently, the group of Hoye published the dimerization reaction of polyalkynes proceeding via benzocyclobutadiene intermediates, in which a C≡C triple bond is broken.^[12]^ This reaction will be discussed in detail later. More common methods of alkyne activation include the use of stoichiometric organometallic reagents and oxidants,^[13]^ as well as oxidative, metal‐free nitrogenation reactions of terminal alkynes, forming arylnitriles.^[14]^ Catalytic reactions involving ruthenium‐, gold‐ and palladium‐complexes can be utilized for the cleavage of C≡C triple bonds.^[15]^ Recently, rhodium‐promoted C≡C triple bond cleavage of diynes, including benzoic acid as a benzyne precursor, was reported by the group of Tanaka (Figure 3).^[16]^

**Figure 4:**
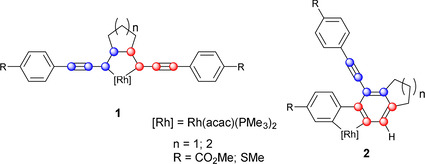
Rhodacyclopentadiene **1** and rhodium 2,2’‐biphenyl complex **2**.

Our group has been exploring the reactivity of metal complexes with 1,4‐diaryl‐1,3‐butadiynes and with α,ω‐bis(arylbutadiynyl)alkanes.^[17]^ In addition to the expected rhodacyclopentadienes **1**[^17a^, ^17b^, ^17c^] formed via metal‐mediated coupling of two alkynes, we have also reported the formation of rhodium 2,2’‐biphenyl complexes **2** in high yields (Figure 4),^[17e]^ which appear to arise from the trapping of an HDDA‐formed benzyne at the rhodium center followed by *ortho*‐CH activation and transfer of the resulting hydride to the β‐carbon atom. With the apparent formation of a benzyne intermediate in mind, we decided to explore metal‐free reactions of our *α*,ω‐bis(arylbutadiynyl)alkanes.

## Results and Discussion

**Figure 5:**
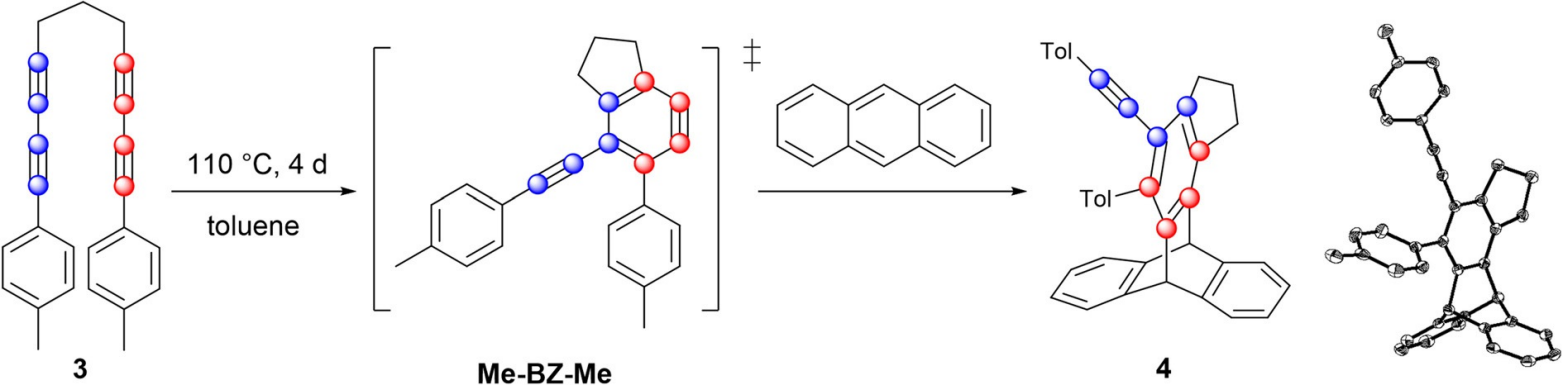
Left: Synthesis of **4** by trapping of the benzyne intermediate with anthracene. Right: Molecular structure of **4** in the solid state (ellipsoids set at 50 % probability). Hydrogen atoms and disorder of (CH_2_)_3_ are omitted for clarity.

In order to establish that indeed an HDDA reaction is operating, we turned to an established benzyne trapping reagent to confirm the HDDA pathway, and heated bisdiyne **3** in the presence of an equimolar amount of anthracene in toluene (Figure 5, left). This reaction gave the triptycene derivative **4** in 75 % yield, which clearly shows that an HDDA reaction is operative (Figure 5, right). In addition to confirming the formation of the benzyne intermediate, the photophysical properties of the reaction product **3** were investigated. Experimental details, as well as full NMR, HRMS, CHN, and single‐crystal X‐ray diffraction data can be found in the Supporting Information.

**Figure 6:**
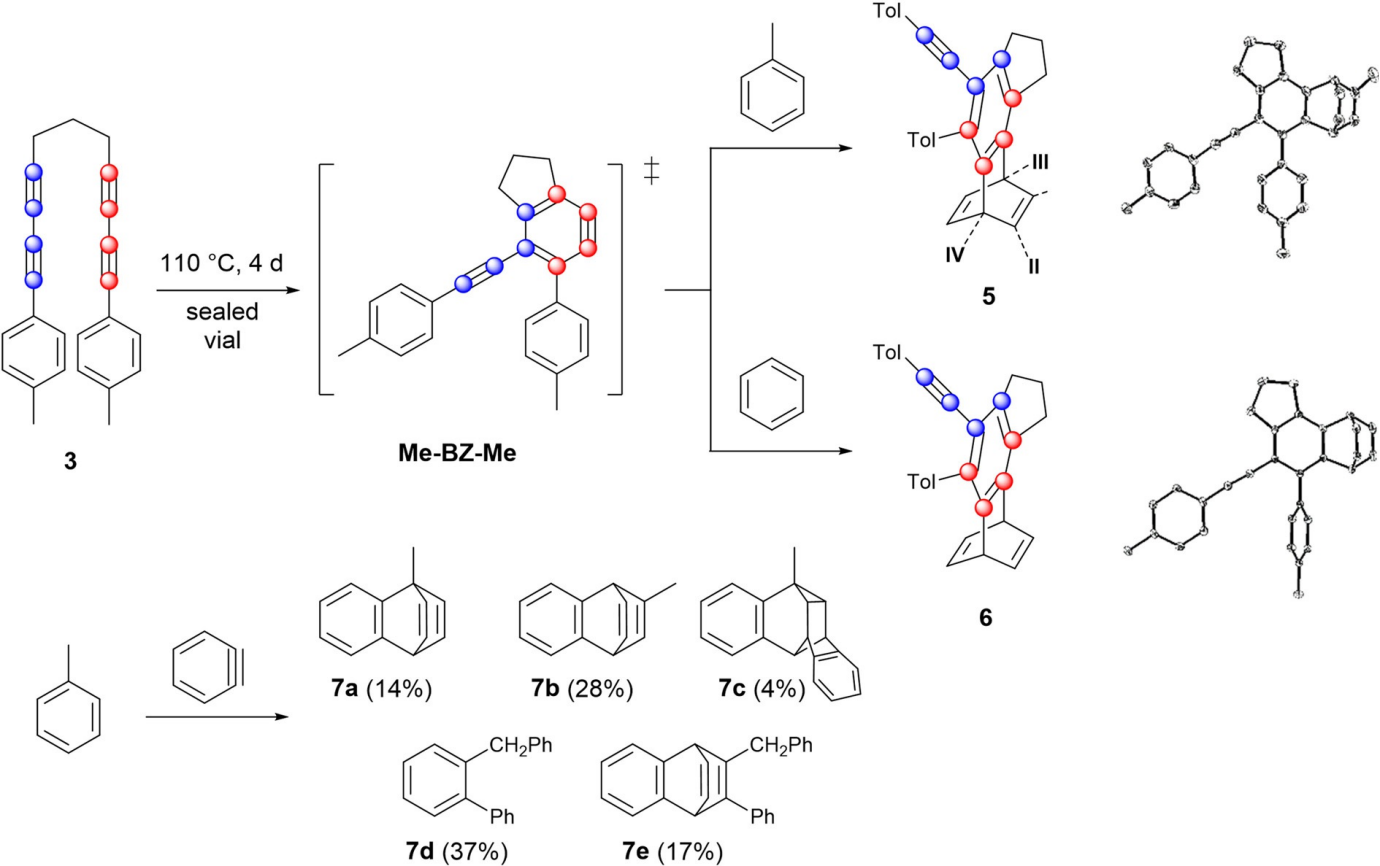
Top left: Trapping of the benzyne intermediate by aromatic solvents toluene, showing the four positional isomers, and benzene. Top right: Molecular structures of **5I** (top) and **6** (bottom) in the solid state. Hydrogen atoms are omitted for clarity and atomic displacement ellipsoids are drawn at 50 % probability. Bottom: The product distribution of the reaction of benzyne with toluene, as reported by Oda et al.^[20]^

In addition to the classical trapping reagents such as anthracene, benzyne intermediates can also react with aromatic solvents (e.g. toluene, benzene) in a [4+2] manner (Figure 6, top left), cycloalkanes and THF in a double hydrogen transfer reaction^[18]^ or, less common, with dichloromethane and acetonitrile.^[19]^ Five different modes (Figure 6, bottom) for the reaction between toluene and benzyne are described in the literature.^[20]^ Considering that our bisdiyne **3** has two tolyl substituents, we decided to test the thermal reaction of **3** with aromatic solvents in order to give us an indication of what to look for if a reaction of the benyzne (**Me‐BZ‐Me**) occurs with a tolyl group of a second molecule of **3**. In our reaction with toluene, we only observe the two reaction modes (**5I** and **5II** similar to **7b**, and **5III** and **5IV** similar to **7a**) that result from a 1:1 reaction of **Me‐BZ‐Me** with toluene. Due to the fact that **Me‐BZ‐Me** is unsymmetrical, this reaction gives four regioisomers **5I**–**5IV** and two enantiomers for **5I** and **5II**. As physical separation of the isomers proved difficult, a detailed analysis of the 2D ^1^H NMR spectra of the mixture allowed assignment of all of the relevant signals to the different isomers (for details see Supporting Information). According to the literature (Figure 6, bottom),[^20^, ^21^] the distribution of isomers in the reaction of benzyne with toluene should be about 2:1 in favor of the 2,5‐addition (**7b**) over the 1,4‐addition (**7a**) product. In our case, there is a distinct preference for isomers **5I** (55 %) and **5II** (33 %), compared to isomers **5III** (8 %) and **5IV** (4 %). This increase in regioselectivity is most likely due to the sterically more demanding substituents on **Me‐BZ‐Me**. The single‐crystal structure of isomer **5I** was obtained (Figure 6, top right). Furthermore, we also examined the thermolysis of **3** in benzene. In stark contrast to the reaction of benzyne with benzene,^[22]^ the benzobarrelene **6** (Figure 6, top right) was observed as the major product. The photophysical properties of the trapping products **5I**–**5IV** and **6** are described later in this paper.

**Figure 7:**
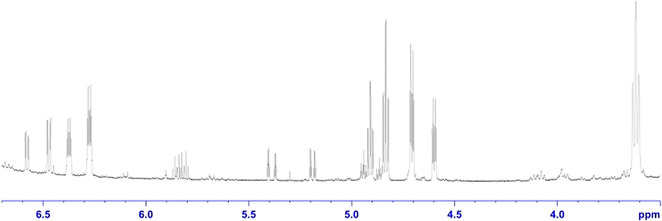
^1^H NMR spectrum (500 MHz, CDCl_3_, r.t.) of the reaction mixture after heating bisdiyne **3** without any other reactants in toluene.

**Figure 8:**
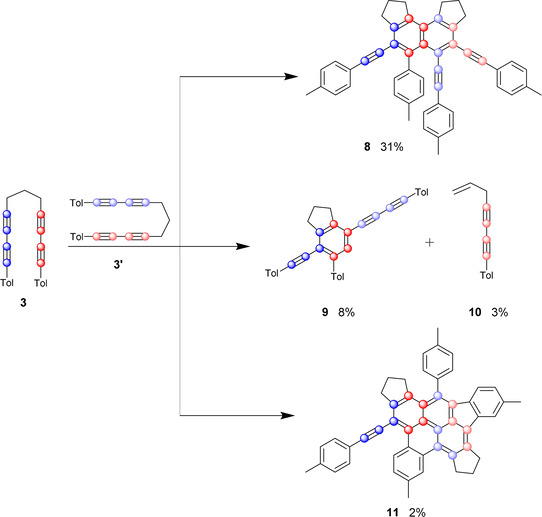
Products of the cannibalistic self‐trapping reaction and their respective isolated yields. Top: Connectivity of **8**, illustrating the cleavage of the C≡C triple bond. Middle: Connectivity of **9**, illustrating the cleavage of the sp–sp^3^ C−C bond. Bottom: Connectivity of **11**, illustrating the cleavage of the C≡C triple bond and the formation of seven rings and nine C−C bonds.

**Figure 9:**
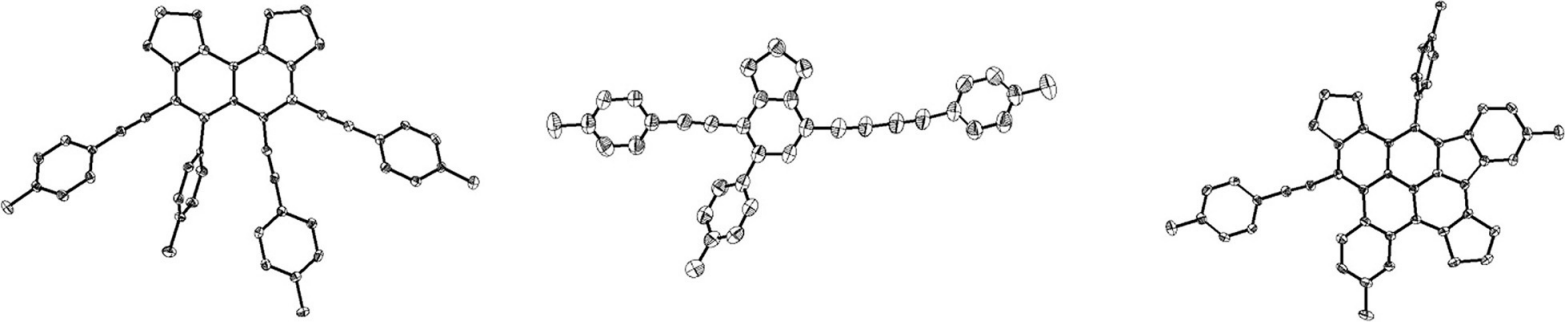
Left: Molecular structure of **8** in the solid state. Co‐crystallized solvent molecules are omitted for clarity. Middle: Molecular structure of **9** in the solid state. Right: Molecular structure of **11** in the solid state. In all three structures, hydrogen atoms are omitted for clarity and ellipsoids are drawn at 50 % probability.

Having demonstrated that the benzyne intermediate **Me‐BZ‐Me** can form in the absence of a metal center, we were intrigued to explore the self‐trapping products resulting from the reaction of **Me‐BZ‐Me** with an additional molecule of the α,ω‐bis(arylbutadiynyl)alkane **3**. The ^1^H NMR spectrum of the reaction mixture in toluene shows a large number of sharp signals (Figure 7), especially in the range of 6.7–3.5 ppm. The signals at 6.7–6.2 ppm and 5.0–4.5 ppm were already assigned to the reaction products of the HDDA‐derived benzyne with toluene. Judging by the integrals of the remaining signals, at least two other products had formed. HRMS analysis of the reaction mixture showed the formation of a dimeric product in addition to other signals. Careful flash chromatography was used to separate the products of the reaction as much as possible. Single‐crystal X‐ray diffraction analysis of the major product (31 % isolated yield) confirmed that a dimeric molecule had formed (Figure 8, top, and Figure 9, left). Inspection of the molecular structure reveals that it is a naphthalene derivative (compound **8**), a type of reaction product recently reported by Hoye from multiple linked polyalkynes.^[12]^ Naphthalene **8** arises from a formal C≡C triple bond cleavage process. The mechanism of this process will be discussed in detail below, but first we shall address the nature of additional intriguing self‐trapping products. One of the other products we identified by HRMS analysis contains one molecule of the α,ω‐bis(arylbutadiynyl)alkane **3** plus a C_11_H_8_ fragment. We were able to isolate and identify this as indane derivative **9**. Apparently, one of the sp–sp^3^ C−C bonds in the second molecule of bisdiyne **3** was cleaved, with the aryl butadiyne (Tol‐C_4_) moiety and an H atom derived from the central methylene group being transferred to the benzyne intermediate **Me‐BZ‐Me** (Figure 8, middle, and Figure 9, middle).

Compound **9** is an uncommon linear π‐system of the form *p*‐(arylethynyl)(arylbutadiynyl)benzene. The reaction mechanism leading to this product will also be discussed in detail later. Furthermore, we were able to isolate the side product **10** from the reaction mixture, which was further characterized by multinuclear NMR spectroscopy and HRMS. The isolation of the second self‐trapping product **9** and resulting fragment **10**, as well as products **4** and **8**, demonstrate that a variety of reactions of the benzyne intermediate are possible, including the cleavage of sp–sp C≡C triple and sp–sp^3^ C−C single bonds. The photophysical properties of compounds **8** and **9** are described later in this paper. The absorption spectra are of importance for another of our publications concerning the direct observation of **Me‐BZ‐Me** in solution via femtosecond transient absorption spectroscopy.^[23]^ Last but not least, another species with a molecular weight of **3**_2_‐2H was detected by HRMS. Single‐crystal X‐ray diffraction analysis revealed it to be a highly unusual pyrene derivative, better described as a benzo‐ [*l*]indeno[*cd*]pyrene **11** (Figure 8, bottom, and Figure 9, right).

**Figure 10:**
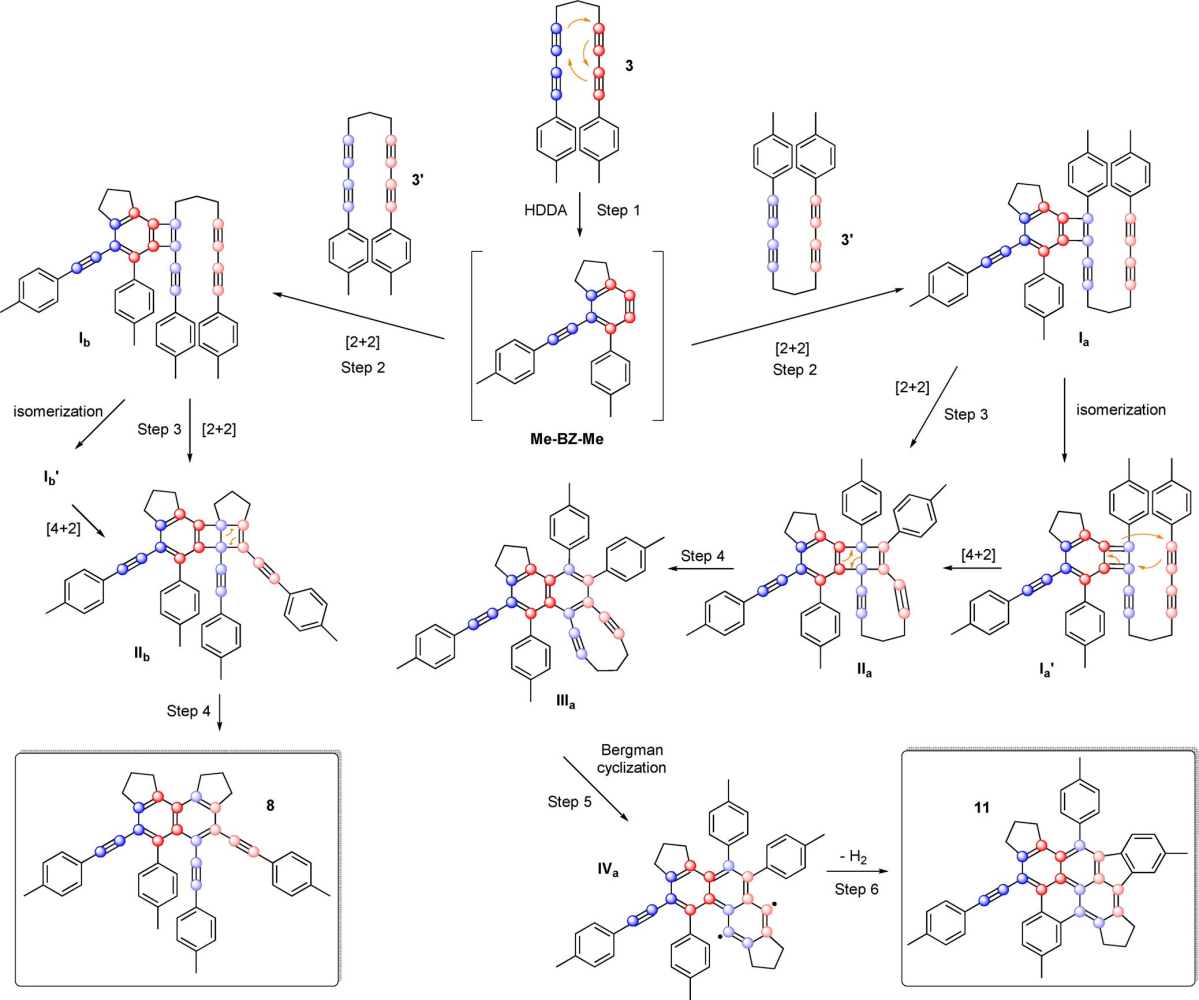
Proposed mechanism for metal‐free C≡C triple bond splitting leading to naphthalene **8** and benzindenopyrene **11** via similar initial reaction steps. The various steps are enumerated for later referencing.

As shown in Figure 10, we propose that the initial step in the formation of compound **11** is an HDDA reaction of **3** followed by a radical type [2+2]‐addition of the benzyne (**Me‐BZ‐Me**) to one alkyne moiety close to the aryl ring in a second bisdiyne **3**’ (Figure 10, right route). [2+2]‐Reactions of benzyne with acetylene derivatives are known to yield benzocyclobutadienes, which often further dimerize to give dibenzocyclooctenes.[^11a^, ^24^] In addition, Johnson et al.^[25]^ reported the analogous [2+2]‐reaction of benzyne with 1,3‐butadiyne. The resulting Dewar benzene (**II_a_**) then isomerizes to benzene (**III_a_**).^[26]^ At this stage, a Bergman cyclization yields a biradical intermediate (**IV_a_**),^[27]^ which then reacts with the two adjacent tolyl rings, yielding the benzo[*l*]indeno[*cd*]pyrene **11**. This product is only generated in small amounts. Nonetheless, the reaction pathways represent a complex combination of an HDDA reaction, benzyne‐alkyne annulation, Bergman cyclization and follow‐up C−C coupling, leading to the formation of a total of seven fused rings and nine C−C bonds, putting the 2 % yield into perspective. A similar mechanism can be proposed for the formation of naphthalene derivative **8** (Figure 10, left route). The difference herein is the triple bond of the second bisdiyne **3**’ (adjacent to the alkyl bridge) that reacts with the benzyne intermediate (**Me‐BZ‐Me**). The following two steps (**I_b_**→**II_b_**→**8**) are the same as described before for **I_a_**→**II_a_**→**III_a_**. Interestingly, naphthalene **8** does not immediately react in a Bergman cyclization as seen in the formation of the benzo[*l*]indeno[*cd*]pyrene **11**. This observation is most likely due to the lack of ring strain in **8**, in comparison to that in the intermediate (**III_a_**). Studies by Snyder show the immediate increase of activation energy by a factor of 1.4 from a propyl‐ (17.9 kcal mol^−1^) to a butyl‐bridged (24.7 kcal mol^−1^) enediyne.^[27c]^ In order to confirm our proposed mechanisms, quantum chemical calculations were carried out.

## Quantum chemical calculations on the dimerization mechanisms

### Computational methods

The stationary points were taken from relaxed scans at the UB3LYP/6‐311++G(d,p)[^28^, ^29^] level, calculated with the Gaussian 09 Rev. E^[30]^ package. Grimme's dispersion correction D3^[31]^ was applied. To investigate the influence of computational approaches on the geometries, we performed single‐point energy calculations with CCSD(T)/aug‐cc‐pVDZ^[32]^ employing the Turbomole 7.0.1^[33]^ program package. M06‐2X‐D3/aug‐cc‐pVDZ^[34]^ calculations were conducted with the Gaussian 09 Rev. E package. Single‐reference approaches are often sufficiently accurate;^[35]^ however, in some cases, multi‐reference approaches are needed to obtain accurate potential energy surfaces (PES),^[36]^ electronically excited states^[37]^ or even properties.^[38]^ To include possible multi‐reference effects, we performed CAS‐SCF/aug‐cc‐pVDZ single‐point calculations. We started with a (2,2) CAS‐space, which was subsequently enlarged stepwise to (4,4)‐, (6,6)‐ and (8,8)‐CAS‐spaces. Dynamic correlation was accounted for by second order Møller‐Plessit perturbation theory within the CAS‐OVB‐MP2^[39]^ method implemented in the Gaussian 09 Rev. E package. For the discussion we use electronic energies.

### Discussion of the various steps

The plausibility of the suggested mechanism depicted in Figure 10 is supported by precedent computations of other groups and new computations that we carried out. The first two steps of the overall reaction (Figure 10) were already computed by Johnson et al.^[25]^ using simpler analogs of the whole system. They found the formation of a benzocyclobutadiene moiety via a biradicaloid intermediate to be the kinetically favored reaction path. In the Supporting Information, the data are summarized in Figure S17 together with a brief description of the computations. Steps 3 and 4 were already computed by Jones and Krebs^[40]^ (Figure S18). However, they used the smaller model system cyclobutadiene with methylacetylene. For this model system, a reaction including the isomerization from **I_b_** and **I_b_** ^**’**^, as indicated in Figure 10, is possible because, for cyclobutadiene, both structures are energetically degenerate. For benzocyclobutadiene this is no longer the case because one bond is part of a benzene ring. Indeed, the energy increases by ca. 45 kcal mol^−1^ if the geometry is changed stepwise from **I_b_** to **I_b_’** in Figure 10. This excludes this pathway. A direct reaction by breaking the bond between the two blue carbon centers of the benzocyclobutadiene can also be excluded because this increases the energy by more than 130 kcal mol^−1^. Hence, only the [2+2] reaction course remains. Its barrier is computed to ca. 22 kcal mol^−1^. This is sufficiently small to make the suggested mechanism plausible. More information, including a detailed description of our computations, can be found in the Supporting Information (Figures S19, S20 and Tables S2 and S3). Step 5 of the overall reaction mechanism, which leads from **III_a_** to **IV_a_** and then, via a formal H_2_‐abstraction, to the final product (**11**), can be seen as a Bergman cyclization. As Snyder^[27c]^ has already shown, a nine‐membered ring enediyne similar to the subunit in molecule **III_a_** can undergo the cycloaromatization step with an activation barrier of only ca. 18 kcal mol^−1^. The resulting *p*‐benzyne subunit of **IV_a_** can then undergo a π‐bond addition to the neighboring *p*‐tolyl groups. Comandini and Brezinsky^[41]^ have calculated such an addition of a phenyl radical to a benzene molecule to have an activation barrier of only ca. 5 kcal mol^−1^.

**Figure 11:**
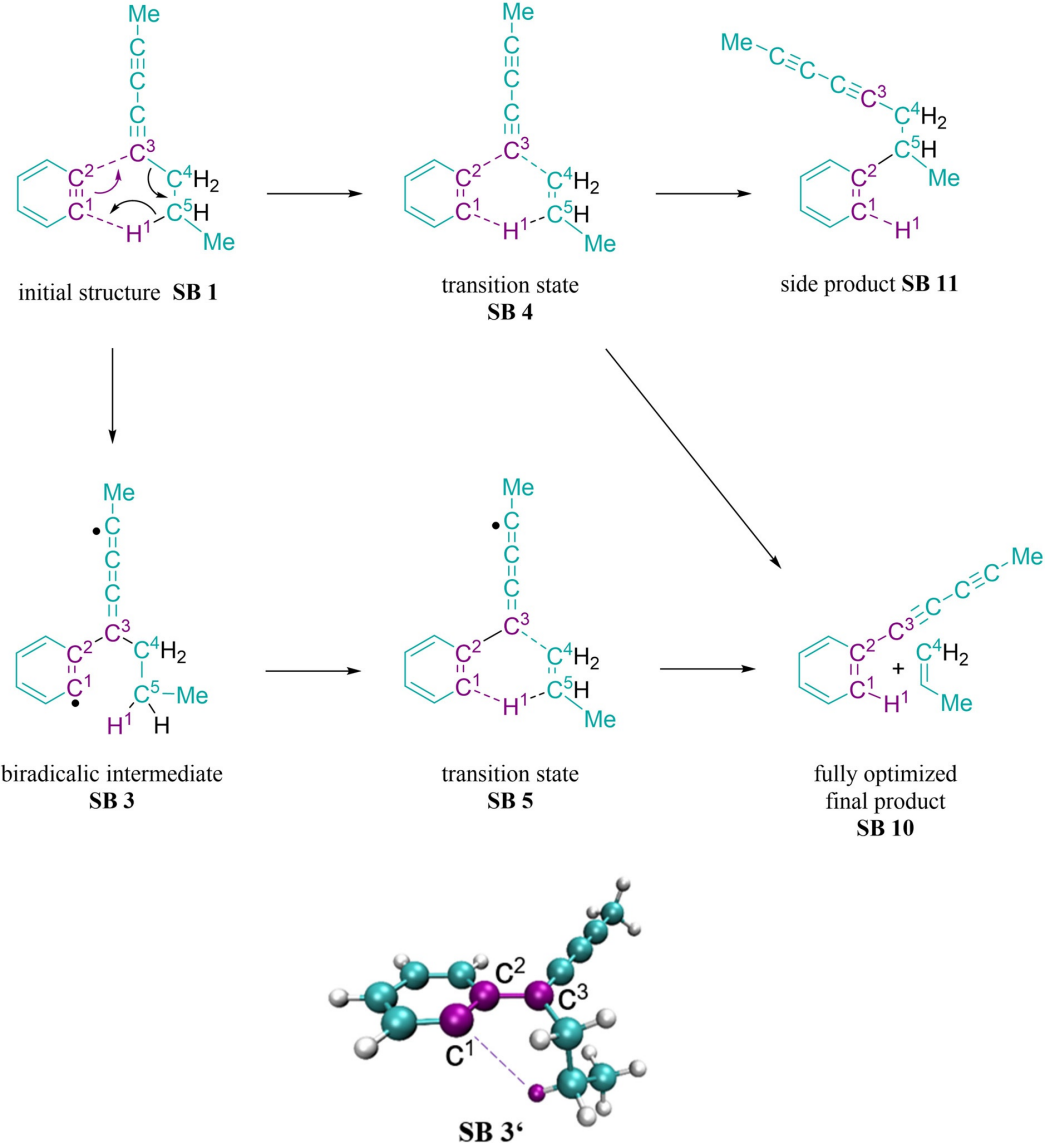
Lewis structures of one reaction path of the reaction of *o*‐benzyne and 1,11‐bis(*p*‐tolyl)undeca‐1,3,8,10‐tetrayne leading to strand cleavage of 1,11‐bis(*p*‐tolyl)undeca‐1,3,8,10‐tetrayne (top), and spatial representation of the fully optimized biradicalic intermediate **SB 3’** (bottom).

**Figure 12:**
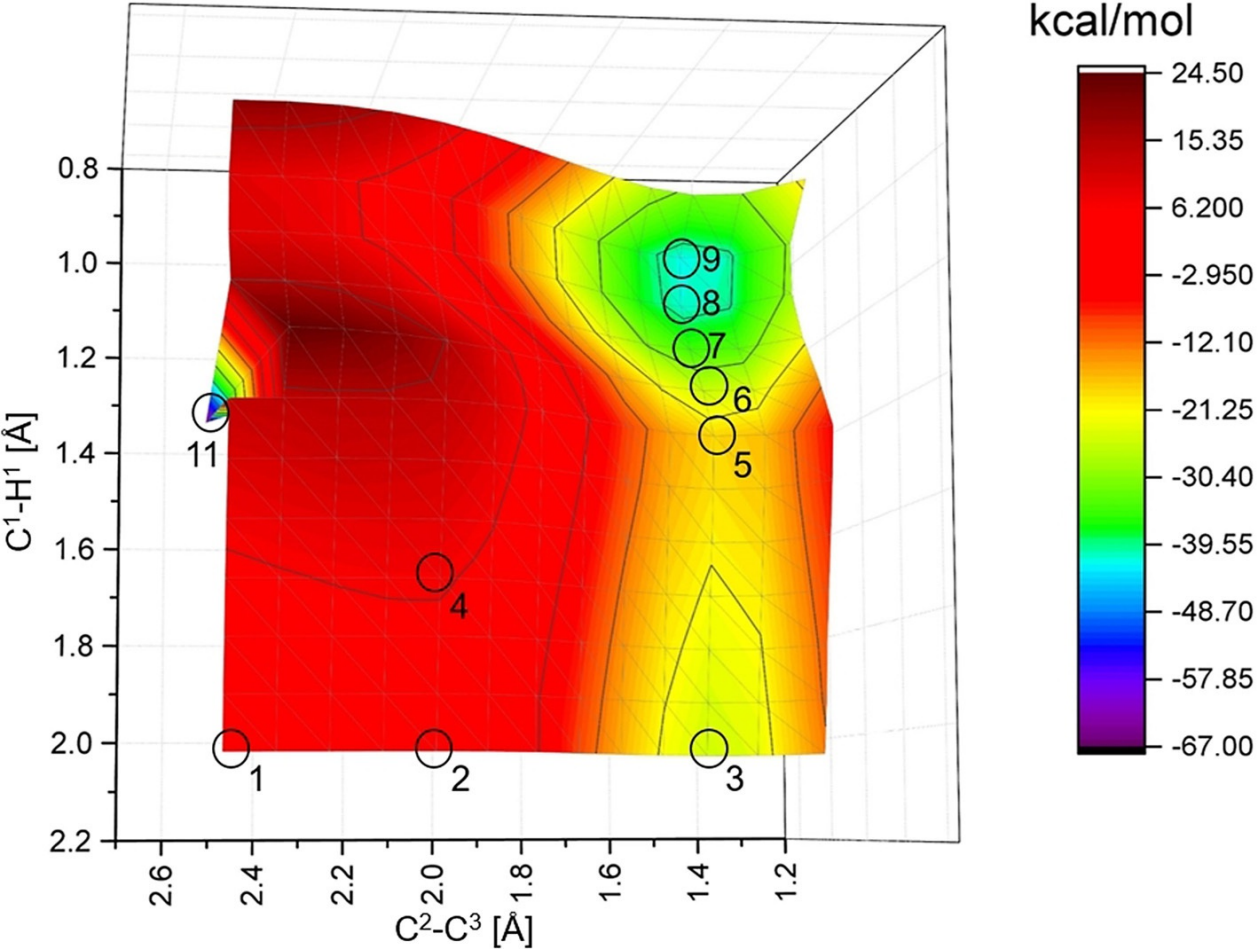
Energy surface of the reaction path of the reaction of *o*‐benzyne and 1,11‐bis(*p*‐tolyl)undeca‐1,3,8,10‐tetrayne leading to strand break of 1,11‐bis(*p*‐tolyl)undeca‐1,3,8,10‐tetrayne. While C^1^−H^1^ and C^2^−C^3^ were varied, all other geometries were optimized using the UB3LYP/6–311++G(d,p) level of theory. The cycles give the points **SB X** for which CAS‐OVB‐MP2(8,8)/aug‐cc‐pVDZ single‐point calculations were performed. The computed relative energies for **SB 1**–**SB 10** are summarized in Table S4 in the Supporting Information. The side product **SB 11** (Figure 11) has not been investigated further because of the high barrier of up to ca. 25 kcal mol^−1^ surrounding this point on the PES.

While the reaction course depicted in Figure 10 was, for the most part, already computed, computations for formation of the two products in Figure 8 (middle) found in the reaction of 1,11‐bis(*p*‐tolyl)undeca‐1,3,8,10‐tetrayne (**3**) have not been reported. Their formation can be explained by strand cleavage reactions between the initially formed *o*‐benzyne (Figure 10) and the C_3_H_6_ alkyl bridge of **3**. A possible mechanism is indicated in Figure 11. To characterize the energies of this mechanism, we computed a two‐dimensional potential energy surface (PES) by varying the leading internal coordinates C^2^−C^3^ and C^1^−H^1^ and optimizing all other coordinates (secondary coordinates) for given values of the two leading internal coordinates. Such PES are necessary if two coordinates are correlated.^[42]^ Figure 12 gives the corresponding surface. The numbering on Figures 11 and 12 correspond to each other. Structure **SB 3’** was obtained by a full geometry optimization starting at point **SB 3** without any restrictions. **SB 10** from Figure 11 represents the fully optimized product, which is separated from **SB 9** by a tiny barrier. As indicated in Figure 11, the reactants (**SB 1**) might lead to the biradical intermediate **SB 3’** if only the new C^2^−C^3^ bond is formed. Due to its reactivity, this biradical intermediate **SB 3** can react with other compounds or rearrange to the product **SB 10** via transition state **SB 5**. The product **SB 10** was observed in the product mixture as compounds **9** and **10**. Product **SB 10**, can also be formed from **SB 1** by a type of concerted one‐step reaction via **SB 4** if bond breaking and formation processes occur simultaneously. On the PES given in Figure 12, the optimization for C^2^−C^3^=2.51 Å and C^1^−H^1^=1.34 Å suddenly leads to structure **SB 11** which shows a completely different arrangement. Because of the strong variation in the arrangement, and the barrier between **SB 1** and **SB 11**, we did not take **SB 11** into consideration.

Figure 12 depicts the energetics of the possible reactions. To keep the computational effort manageable, the necessary geometry optimizations were performed at the UB3LYP‐D3/6‐311++G(d,p) level of theory. Using these geometries, we performed CAS‐OVB‐MP2(8,8) calculations for selected points (see circles in Figure 12) because the computed S^2^ values of the DFT computations indicated that various structures of the PES possess a high degree of biradical character. In such cases, only multi‐reference approaches are sufficiently accurate whether energies,^[36]^ structures,^[43]^ reactions^[44]^ or simple properties^[37b]^ have to be predicted. Hence, in the following we only discuss the CAS‐OVB‐MP2(8,8) results which are summarized in Table S4 in the Supporting Information. The corresponding DFT values differ considerably as is also shown in Table S4.

The CAS‐OVB‐MP2(8,8) values for the points **SB 1‐SB 10** predict that the stepwise reaction (**SB 1**→**SB 3**→**SB 5**→**SB 9**→**SB 10**) is favored with respect to the concerted one (**SB 1**→**SB 4**→**SB 9**→**SB 10**). If one goes from **SB 1** to **SB 3**, a barrier of ca. 19 kcal mol^−1^ (**SB 2**) has to be overcome. For the concerted mechanism, the barrier (**SB 4**) which has to be surmounted to reach the intermediate **SB 9** directly is higher (26 kcal mol^−1^). The biradical intermediate **SB 3’** is ca. 12 kcal mol^−1^ more stable than **SB 1** and represents a local minimum on the reaction surface. However, to reach **SB 9**, the barrier at **SB 5** is only 8–9 kcal mol^−1^. In structure **SB 9**, the hydrogen is already attached to the C^1^ center, but the C^3^−C^4^ bond is not yet broken. However, CAS‐OVB‐MP2(8,8) predicts that the barrier to **SB 10** is less than 1 kcal mol^−1^. In summary, the computed reaction path underlines the plausibility of the mechanism leading to the product compounds **9** and **10**. For more information, see Supporting Information.

### Photophysical measurements

**Figure 13:**
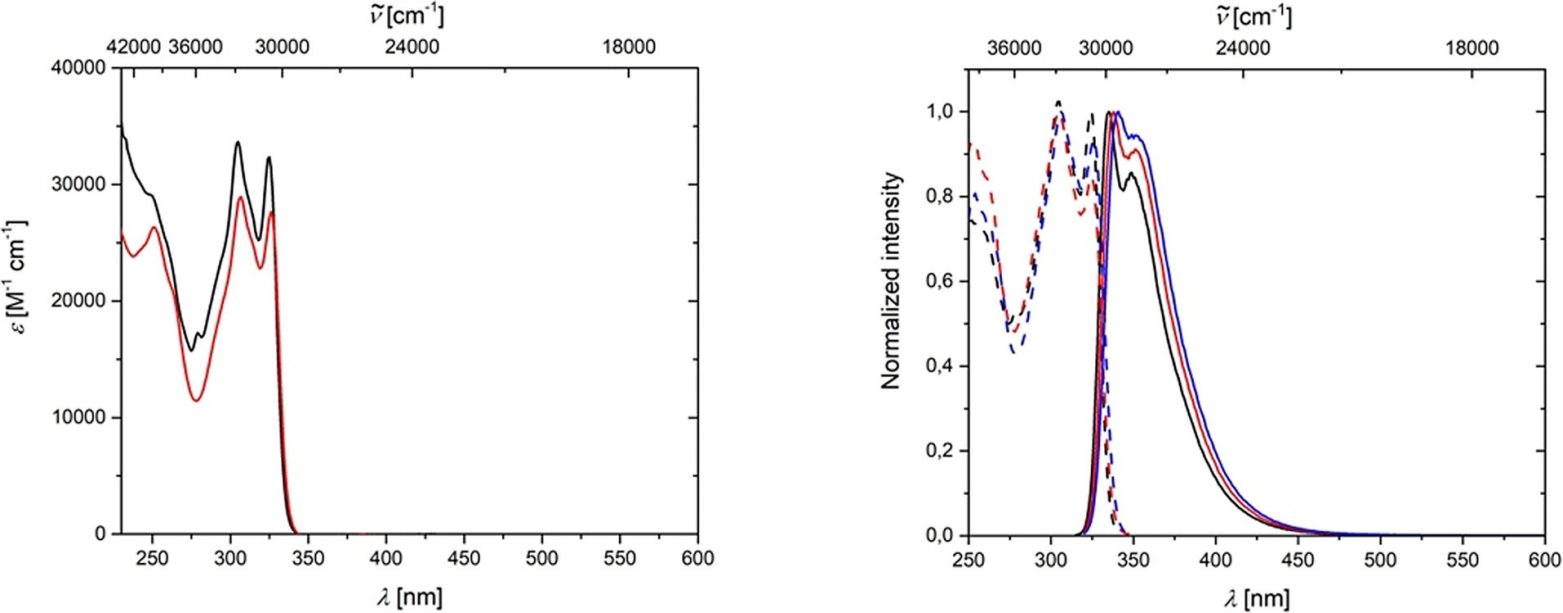
Left: Absorption spectra of **4** (black) and **6** (red) in CH_2_Cl_2_ solution. Right: Excitation (dashed) and emission (solid) spectra of **4** (black), isomers of **5** (blue) and **6** (red) in CH_2_Cl_2_ solution.

Triptycene **4**, the mixture of isomers of toluene adducts **5**, and the benzene adduct **6** all show very similar absorption, excitation and emission spectra (Figure 13). This indicates that the substituents attached to the barrelene core of **6** (methyl group for isomers of **5** and the two fused benzene rings for **4**) do not have a significant influence on the absorption and emission spectra. The first absorption maxima of **4** and **6** are located at 325 and 326 nm, respectively, and the second maxima are at 305 and 307 nm, respectively. The first emission maxima of **4** and **6** are located at 335 and 338 nm, respectively, and the second at 349 and 351 nm, respectively. The lifetime of **4** is *τ*=1.1 ns and the quantum yield is 54 %. In contrast, compound **6** has a significantly weaker emission with a quantum yield of only 5 % and a lifetime shorter than 1 ns. Thus, even though the absorption/excitation/emission spectra are not greatly influenced by the aforementioned substitution, a significant difference in quantum yield is observed due to differences in non‐radiative rate constants. No lifetime or quantum yield were measured for the mixture of the isomers of **5**. The calculated absorption spectra of compounds **4**, **5**, **6**, **8**, **9** and **11** (see Supporting Information) are in good agreement with the experimental absorption spectra.

**Figure 14:**
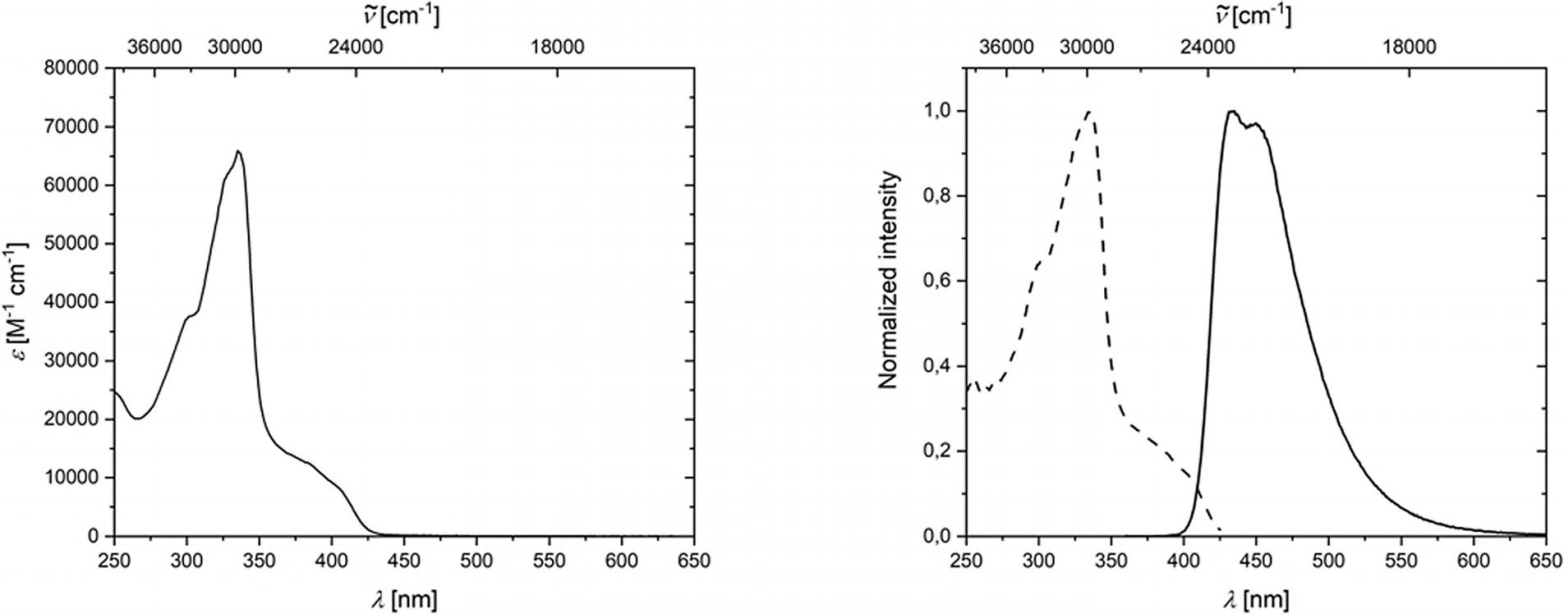
Left: Absorption spectrum of **8** in CH_2_Cl_2_ solution. Right: Excitation (dashed) and emission (solid) spectra of **8** in CH_2_Cl_2_ solution.

The absorption spectrum of naphthalene **8** displays a strong band at 336 nm and a shoulder from 360 to 420 nm (Figure 14, left). A broad emission from 400 to 600 nm with two maxima at 434 and 450 nm were detected (Figure 14, right). The excited state lifetime of **8** is *τ*=3.9 ns, and the quantum yield was determined to be 43 %.

**Figure 15:**
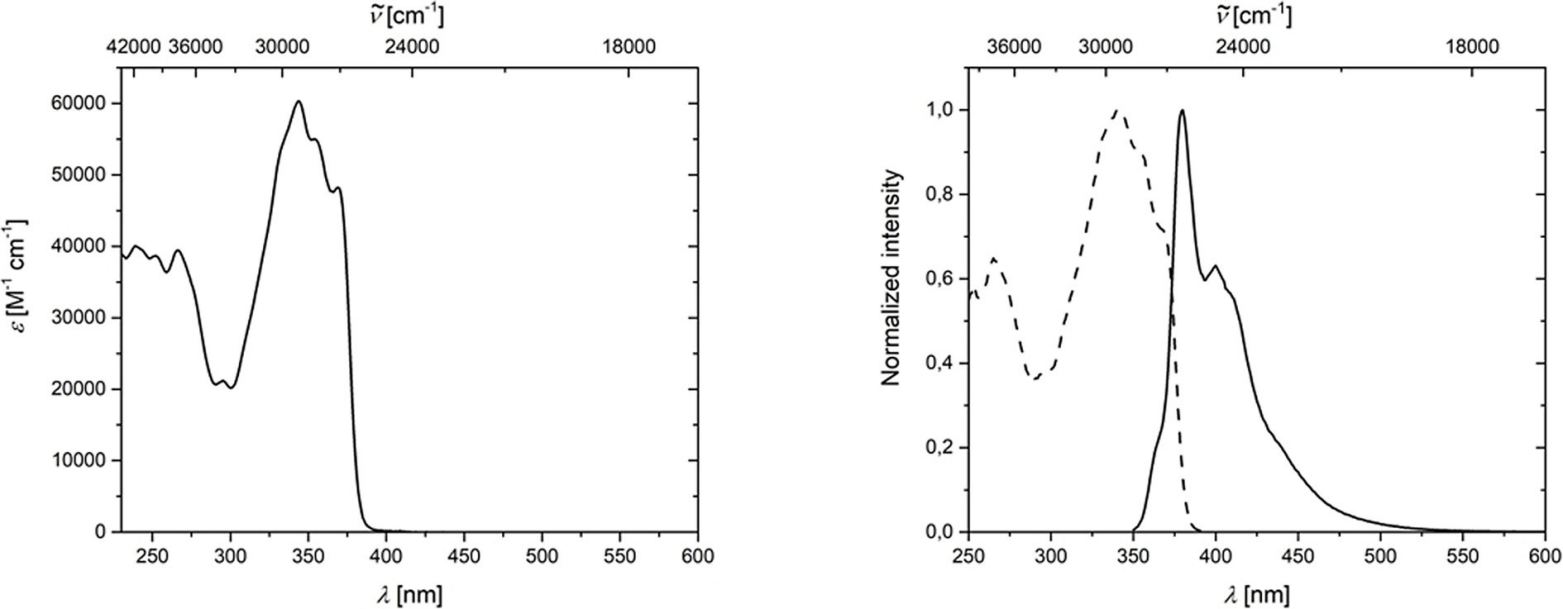
Left: Absorption spectrum of **9** in CH_2_Cl_2_ solution. Right: Excitation (dashed) and emission (solid) spectra of **9** in CH_2_Cl_2_ solution.

The absorption spectrum of indane **9** shows a broad band from 300 to 390 nm, with three maxima at 344/355/370 nm (Figure 15, left). The emission spectrum displays a broad band from 350 to 550 nm, with a sharp maximum at 380 nm and a second maximum at 400 nm (Figure 15, right). The excited state lifetime of **9** is shorter than 1 ns and the quantum yield is 26 %. The UV/VIS absorption properties of compounds **8** and **9**, as mentioned before, are of significant importance to another of our publications, in which we report femtosecond transient absorption spectra of the reaction process going from **3** via the benzyne **Me‐BZ‐Me** to its self‐trapping products **8** and **9**.^[23]^

**Figure 16:**
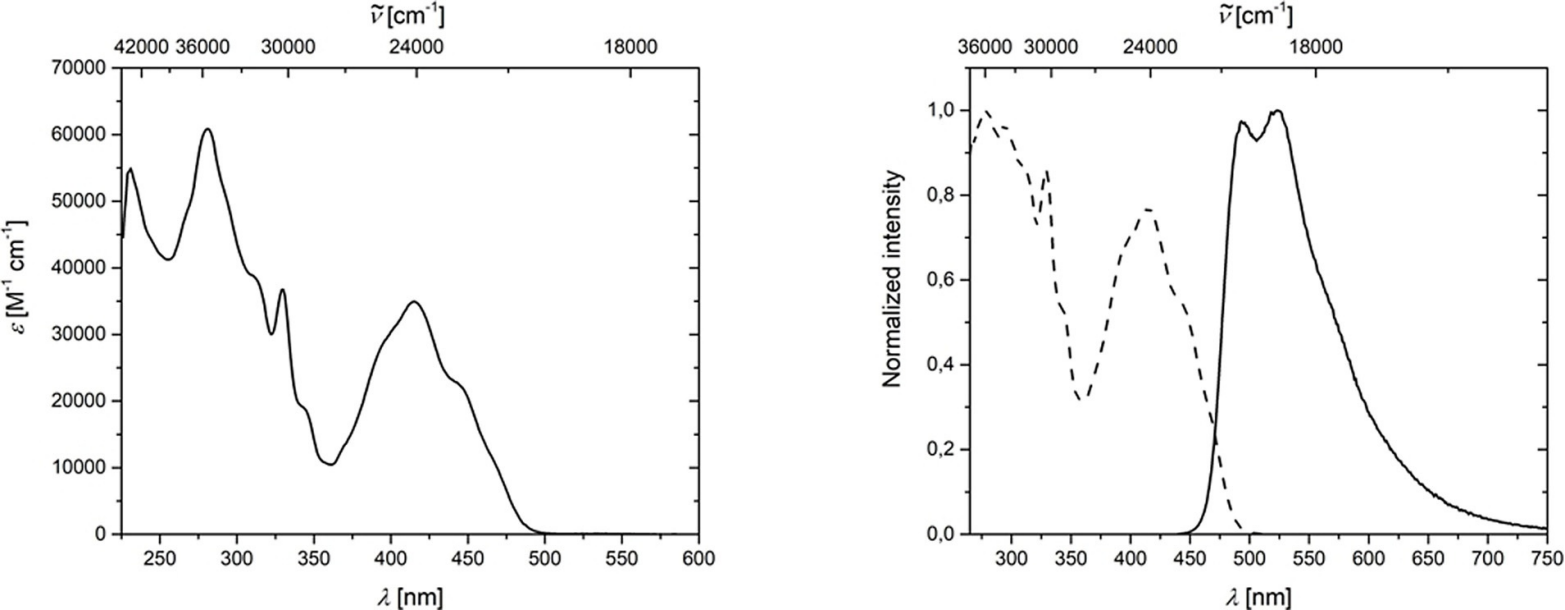
Left: Absorption spectrum of **11** in CH_2_Cl_2_ solution. Right: Excitation (dashed) and emission (solid) spectra of **11** in CH_2_Cl_2_ solution.

Benzo[*l*]indeno[*cd*]pyrene **11** shows a broad absorption from 360 to 500 nm with a maximum at 413 nm, and the strongest absorption band is located at 280 nm (Figure 16, left). A broad emission from 450 to 750 nm with two maxima at 493 and 524 nm were detected (Figure 16, right), and a lifetime of 7.6 ns and quantum yield of 35 % were measured in CH_2_Cl_2_. The calculated absorption spectrum shows that the HOMO→LUMO transition at 403 nm is an allowed transition with an oscillator strength of 1.0, and another absorption, which has major contributions (33 %) from the HOMO→LUMO+1 transition occurs at 290 nm.

**Figure 17:**
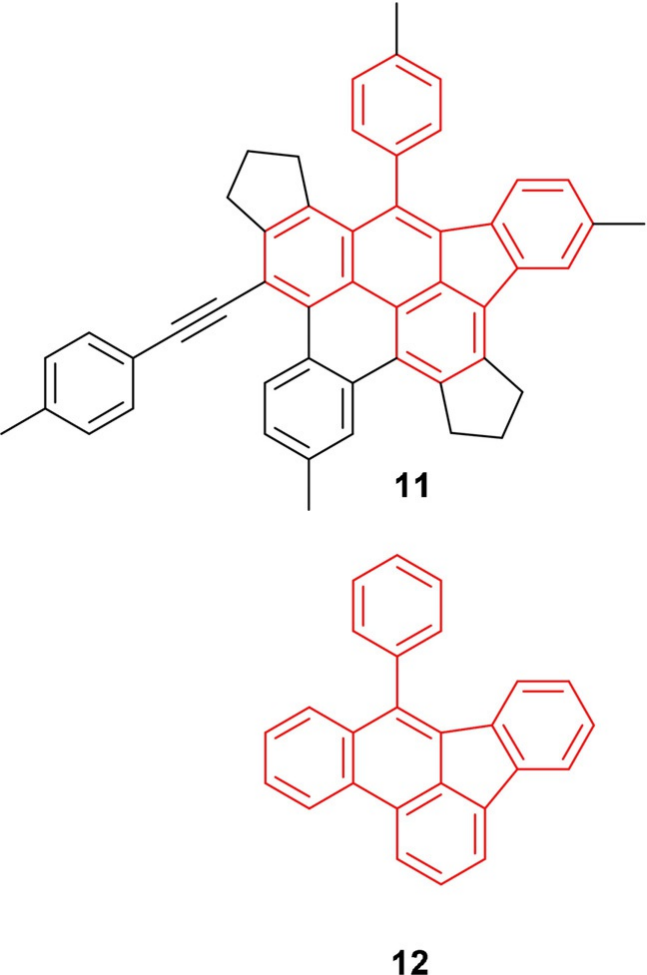
Structural relationship of compounds **11** and **12**.

Categorizing compound **11** as a pyrene derivative might seem logical from a purely structural standpoint; however, the photophysical properties are not comparable to pyrene, which might result from the substitution of every position around the aromatic core. In order to determine the type of aromatic core that best describes compound **11**, NICS calculations (details in the Supporting Information) were carried out. They revealed that its structure is better described as a phenanthrene derivative than a pyrene derivative. A comparison of the absorption spectra of compound **11** with that of the related compound **12** (Figure 17) reported by Tang et al.,^[45]^ also shows significant differences. The absorption spectrum of **12** in acetonitrile shows a broad band from 330 to 370 nm with a maximum around 355 nm. A more intense absorption was detected close to 325 nm, but this is not shown in detail in the publication of Tang et al.

Even though the structure suggests a pyrene core and the NICS calculations suggest phenanthrene as the basic motif, it is not trivial to compare the aromatic core to other, simple aromatic molecules, due to the high degree of substitution. Thus, the photophysical properties of novel compound **11**, and possible derivatives thereof, synthesized in a single step from the respective 1,11‐bis(aryl)undeca‐1,3,8,10‐tetrayne, are of general interest.

## Conclusions

In summary, we have been able to confirm that the exemplary compound 1,11‐bis(*p*‐tolyl)undeca‐1,3,8,10‐tetrayne **3** converts, via an HDDA reaction, into a highly reactive benzyne intermediate, which then either reacts in a metal‐free reaction with the toluene or benzene solvent or, more interestingly, in a cannibalistic self‐trapping process with another molecule of bisdiyne **3** in at least three different ways. We have isolated and fully characterized naphthalene **8**, indane **9** and a fascinating benzo[*l*]indeno[*cd*]pyrene **11** as reaction products. The reaction mechanisms were investigated by high‐level calculations, revealing the process leading to the cleavage of C≡C triple and sp–sp^3^ C−C single bonds. This provides considerable insight into the diverse reactivity of the benzyne intermediate which, in our case, generates nine C−C bonds and seven rings in the formation of compound **11**. Investigations on further reaction products and other reaction pathways arising from this system are currently underway. Preliminary results show similar reaction processes for bisdiynes containing donor or acceptor substituents at the *para*‐position of the aryl ring, indicating the generality of the reactions we have observed. These derivatives, and their photophysical properties, will be reported in due course.

## Experimental Section

**Crystallographic data**: Deposition numbers 1940941 (**3**), 1940942 (**4**), 1940945 (**5I**), 1940946 (**6**), 1940943 (**8**), 1940944 (**9**), and 1940947 (**11**) contain(s) the supplementary crystallographic data for this paper. These data are provided free of charge by the joint Cambridge Crystallographic Data Centre and Fachinformationszentrum Karlsruhe Access Structures service.

## Conflict of interest

The authors declare no conflict of interest.

## Supporting information

As a service to our authors and readers, this journal provides supporting information supplied by the authors. Such materials are peer reviewed and may be re‐organized for online delivery, but are not copy‐edited or typeset. Technical support issues arising from supporting information (other than missing files) should be addressed to the authors.

Supplementary
