# Supplementary material for: Highly Conjugated π‐Systems Arising from Cannibalistic Hexadehydro‐Diels–Alder Couplings: Cleavage of C−C Single and Triple Bonds
Source: Chemistry. 2020 Oct 29;26(68):15989–6000. doi: 10.1002/chem.202002511 (PMC7756338; doi:10.1002/chem.202002511)
Supplement: Supplementary file 1 — Supplementary [file CHEM-26-15989-s001.pdf]

Chemistry–A European Journal

Supporting Information

Highly Conjugated π-Systems Arising from Cannibalistic Hexadehydro-Diels–Alder Couplings: Cleavage of C–C Single and Triple Bonds

Jan Maier,[a] Marian Deutsch,[b] Julia Merz,[a] Qing Ye,[a, c] Oliver Diamond,[a] Maja-Tessa Schilling,[a] Alexandra Friedrich,[a] Bernd Engels,*[b] and Todd B. Marder*[a]

SUPPORTING INFORMATION

Table of Contents

Experimental section....................2

Photophysical measurements....................23

Crystal structure determinations....................28

Computational details 1....................35

Computational details 2....................74

References....................100

Experimental section

General Information

Unless otherwise noted, all reactions were performed using standard Schlenk or glovebox (Innovative Technology Inc.) techniques under argon. HPLC grade solvents were argon saturated, dried using an Innovative Technology Inc. Pure-Solv Solvent Purification System, and further deoxygenated by using the freeze-pump-thaw method. The compounds 1-ethynyl-4-methylbenzene[1] and 1,7-dibromohepta-1,6-diyne were prepared *via* a modification of a bromination procedure employed for the synthesis of bromoacetylenes.[2] All other starting materials were purchased from commercial sources and used without further purification.

Microwave-heating was performed in a Biotage® Initiator+ reactor. Automated flash chromatography was performed using a Biotage® Isolera Four System on silica gel (Biotage® SNAP cartridge KP-Sil 10 g, KP-Sil 25 g and HP-Sil 50 g). Commercially available, precoated TLC plates (Polygram® Sil G/UV_{254} and Polygram® Alox N/UV_{254}) were purchased from Machery-Nagel. The removal of solvent was performed on a rotary evaporator *in vacuo* at a maximum temperature of 45 °C.

GC-MS analyses were performed using an Agilent 7890A gas chromatograph equipped with an Agilent 5975C inert MSD with triple-axis detector operating in EI mode and an Agilent 7693A series auto sampler/injector. Elemental analyses were performed in the Institute of Inorganic Chemistry, Würzburg on an Elementar vario MICRO cube elemental analyzer. High resolution mass spectra were obtained using a Thermo Fisher Scientific Exactive™ Plus Orbitrap MS System in $APCI^+$ mode.

NMR spectra were recorded at ambient temperature using a Bruker DRX-300 (^1H, 300 MHz; $^{13}C\{^1H\}$, 75 MHz), or a Bruker Avance 500 NMR (^1H, 500 MHz; $^{13}C\{^1H\}$, 125 MHz) spectrometer. ^1H NMR chemical shifts are referenced *via* residual proton resonances of the corresponding deuterated solvent ($CDCl_3$: 7.26 ppm) whereas $^{13}C\{^1H\}$ NMR spectra are referenced *via* the carbon signals of the deuterated solvent ($CDCl_3$: 77.16 ppm).

Synthesis of 3

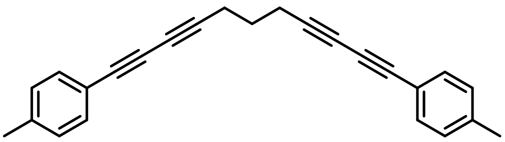

The compounds CuCl (40 mg, 0.40 mmol) and $NH_2OH{\cdot}HCl$ (28 mg, 0.40 mmol) were added to a degassed solution of *n*-$BuNH_2$ (50 mL) and MeOH (450 mL). One equivalent of 1,7-dibromohepta-1,6-diyne (2.00 g, 8.00 mmol) was added and the mixture was cooled to 0 °C. Two equivalents of 1-ethynyl-4-methylbenzene (1.85 g, 16.0 mmol) were added and the reaction mixture was stirred for 30 min at 0 °C. Then, the reaction mixture was heated to 75 °C and stirred for 1.5 h, after which the volatiles were removed *in vacuo*. The resulting residue was recrystallized from CH_2Cl_2/hexane over night at −30 °C to give **3** as an off-white solid. Yield: 1.34 g (52%).

^{1}H NMR (500 MHz, $CDCl_3$, r.t., ppm): δ = 7.38 (m, 4H, CH), 7.11 (m, 4H, CH), 2.53 (t, J = 7 Hz, 4H, CH_2), 2.35 (s, 6H, CH_3), 1.84 (quin., J = 7 Hz, 2H, CH_2).

^{13}C{^{1}H} NMR (125 MHz, $CDCl_3$, r.t., ppm): δ = 139.4, 132.6, 129.3, 118.9, 82.8, 75.6, 73.7, 66.3, 27.1, 21.7, 18.9.

Elem. Anal. Calc. (%) for $C_{25}H_{20}$: C 93.71, H 6.29; found: C 93.45, H 6.35.

HRMS (APCI$^+$): [$C_{25}H_{20}$ + H^+] calc.: m/z 321.1638; found: m/z 321.1636 (|Δ| = 0.62 ppm)

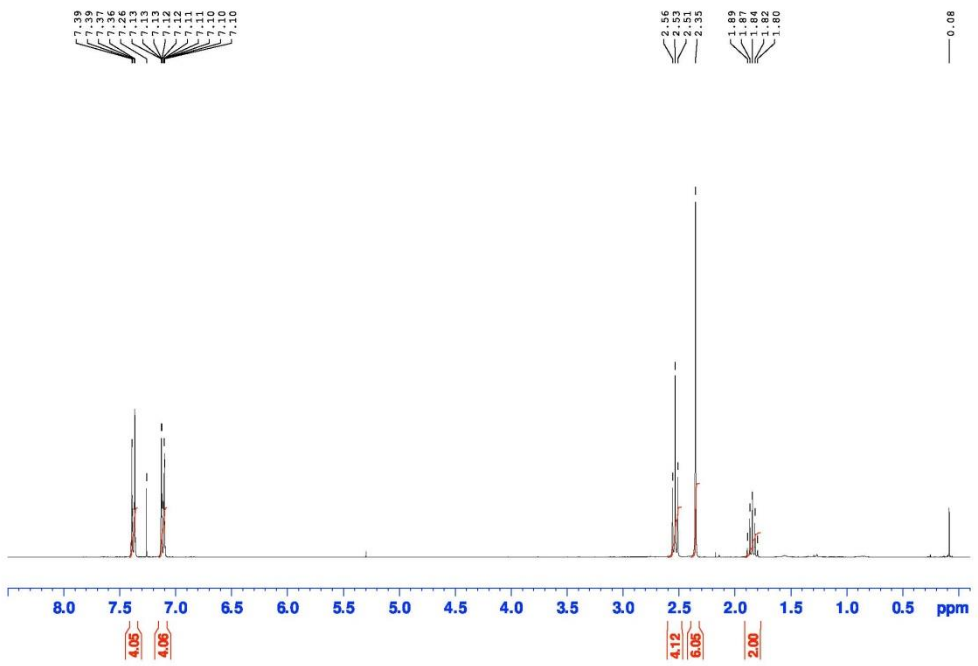

^{1}H NMR spectrum, 500 MHz ($CDCl_3$).

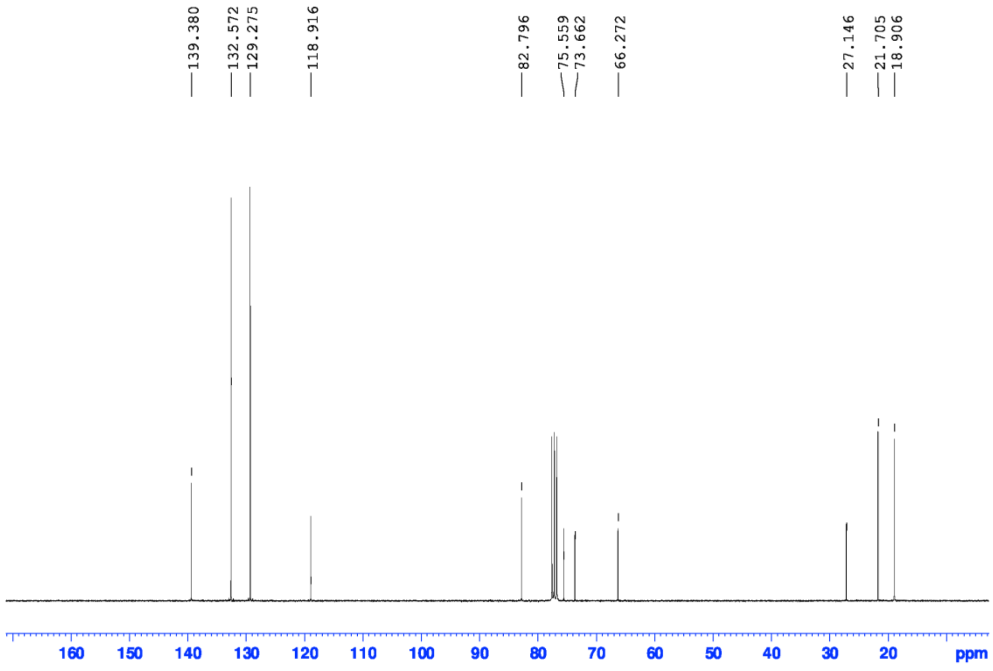

^{13}C{^{1}H} NMR spectrum, 125 MHz ($CDCl_3$).

Synthesis of triptycene 4

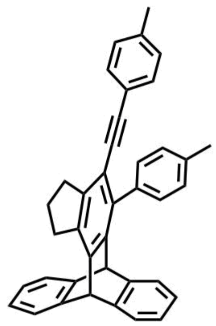

In an argon-filled glovebox, a toluene (ca. 10 mL) solution of 1,11-bis(*p*-tolyl)undeca-1,3,8,10-tetrayne (**3**) (0.10 g, 0.31 mmol) and anthracene (56 mg, 0.31 mmol) was prepared in a sealable 25 mL microwave vial equipped with magnetic stirring bar. The vial was subsequently crimp sealed and heated with stirring to 110 °C for 4 d, after which the mixture was cooled to room temperature. The solvent was removed in *vacuo*. The residue was purified by flash chromatography, using a gradient of hexane/CH_2Cl_2 (0% to 5% CH_2Cl_2) as eluent, yielding **4** as a bright yellow solid (0.12 g, 75%).

^{1}H NMR (500 MHz, $CDCl_3$, r.t., ppm): δ = 7.44–7.34 (m, 6H, CH), 7.28–7.26 (m, overlap with chloroform peak, 2H, CH), 7.10–6.98 (m, 8H, CH), 5.54 (s, 1H, $CH_{bridgehead}$), 5.49 (s, 1H, $CH_{bridgehead}$), 3.19 (t, J = 8 Hz, 2H, CH_2), 3.07 (t, J = 8 Hz, 2H, CH_2), 2.55 (s, 3H, CH_3), 2.32 (s, 3H, CH_3), 2.17 (quin., J = 8 Hz, 2H, CH_2).

^{13}C{^{1}H} NMR (125 MHz, $CDCl_3$, r.t., ppm): δ = 145.6, 145.2, 144.5, 141.7, 140.8, 137.8, 137.6, 137.2, 136.7, 136.3, 131.3, 130.4, 129.0, 128.7, 125.4, 125.2, 123.80, 123.78, 121.0, 115.7, 94.6, 87.9, 51.8, 51.4, 33.5, 31.1, 24.8, 21.58, 2 x 21.57.

Elem. Anal. Calc. (%) for $C_{39}H_{30}$: C 93.94, H 6.06; found: C 93.94, H 6.31.

HRMS (APCI$^+$): [$C_{39}H_{30}$ + H^+] calc.: m/z = 499.2420; found: m/z = 499.2411 (|Δ| = 1.80 ppm)

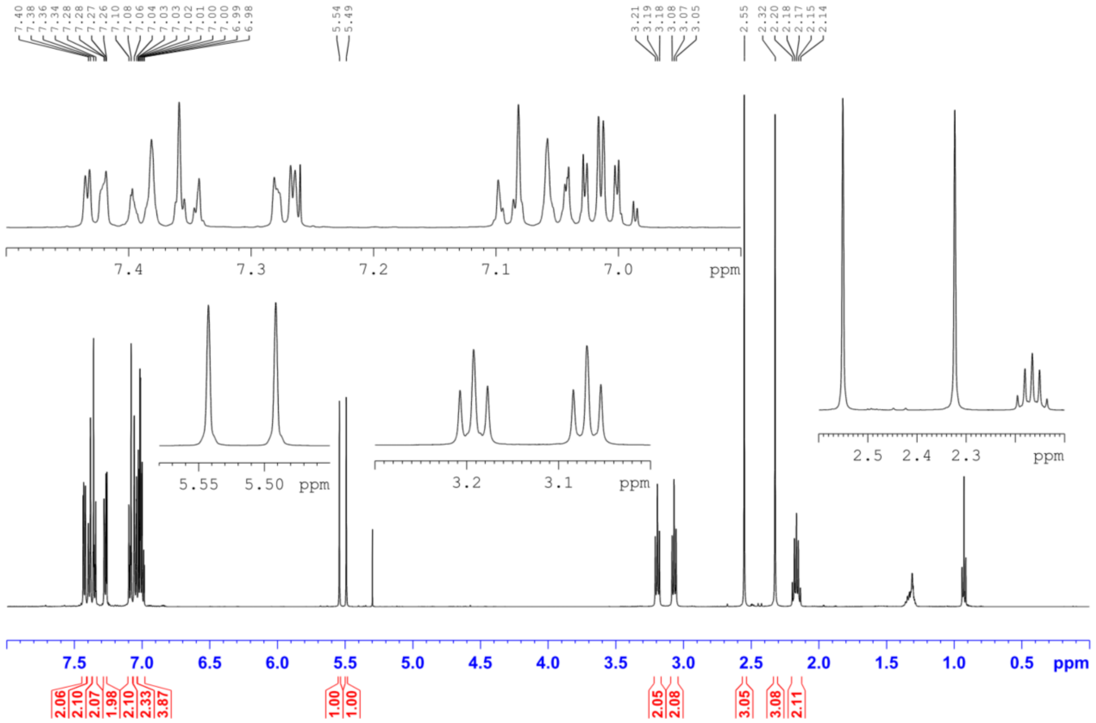

^{1}H NMR spectrum, 500 MHz ($CDCl_3$).

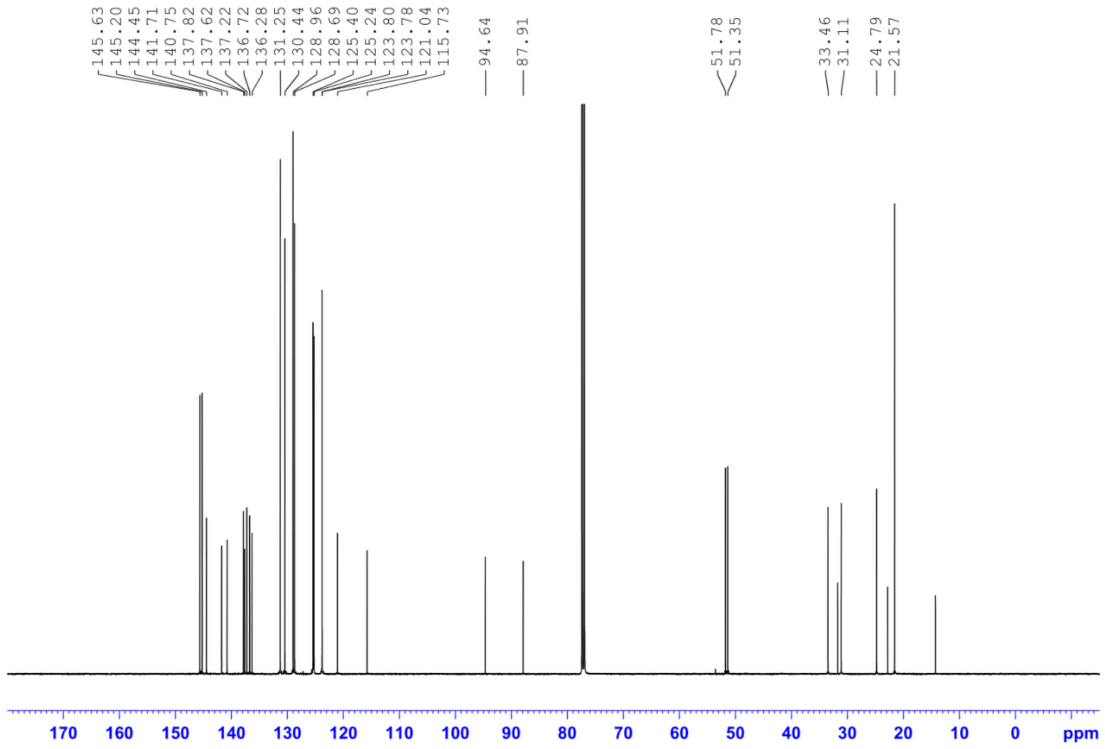

^{13}C{^{1}H} NMR spectrum, 125 MHz ($CDCl_3$).

Synthesis of toluene adduct 5

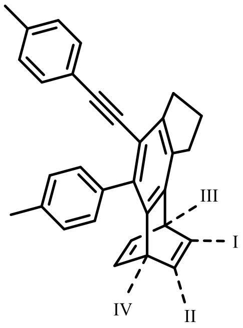

The compound 1,11-bis(*p*-tolyl)undeca-1,3,8,10-tetrayne **3** (0.50 g, 1.56 mmol) was dissolved in 125 mL of degassed toluene and stirred at 110 °C for 4 d under an argon atmosphere. The solvent was evaporated, the dark yellow solid was re-dissolved in CH_2Cl_2 and rotary-evaporated onto silica. Following flash column chromatography, using hexane/CH_2Cl_2 (0% to 100% CH_2Cl_2) as eluent, the solvent from the fourth fraction was evaporated. The crude product was re-dissolved in hexane/toluene (4:1) and recrystallized by slow solvent evaporation giving a mixture of four regioisomers of **5** (**I** (55%); **II** (33%); **III** (8%); **IV** (4%)) as colorless crystals. Crude yield: 80 mg (0.19 mmol, 12%)

HRMS (APCI$^+$): [$C_{32}H_{28}$ +H^+] calc.: m/z = 413.2264; found: m/z = 413.2253 (|Δ| = 2.66 ppm)

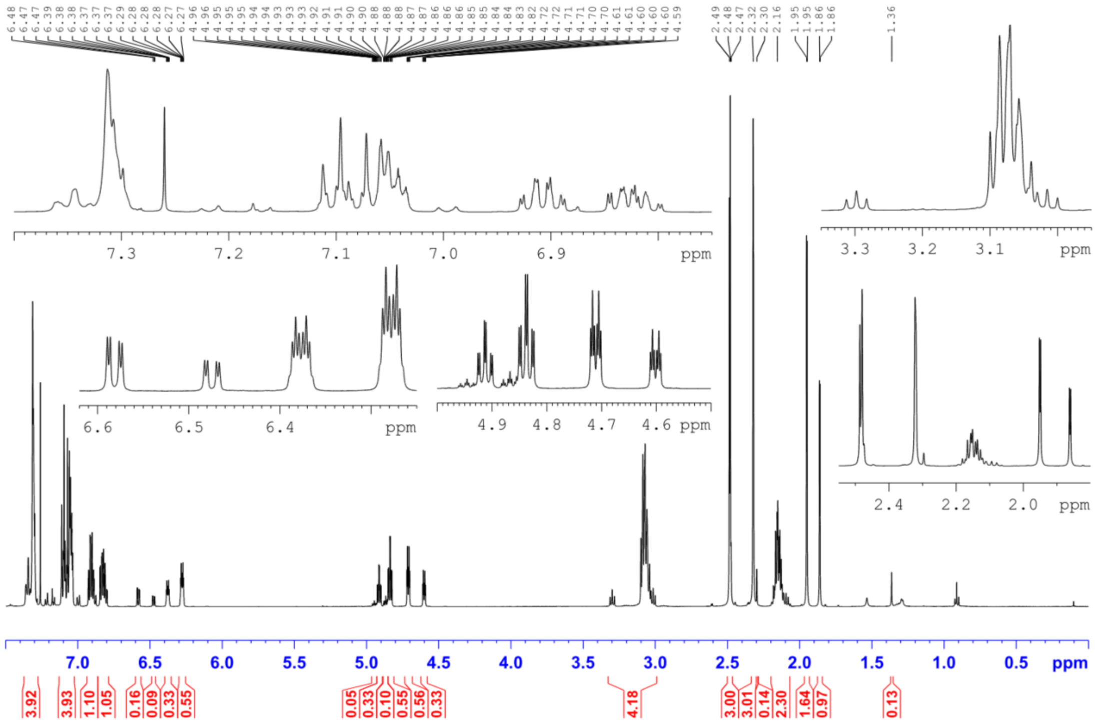

^1H NMR spectrum, 500 MHz ($CDCl_3$).

Isomer **5I**:

^{1}H NMR (500 MHz, $CDCl_3$, r.t., ppm): δ = 7.38−7.28 (m, 4H, CH), 7.23−6.98 (m, 4H, CH_{arom}), 6.93−6.87 (m, 1H, CH), 6.85−6.79 (m, 1H, CH), 6.28 (dt, J = 6; 2 Hz, 1H, CH), 4.84 (td, J = 6; 2 Hz, 1H, $CH_{bridgehead}$), 4.71 (dt, J = 6; 2 Hz, 1H, $CH_{bridgehead}$), 3.11−2.99 (m, 4H, CH_2), 2.49−2.47 (s, 3H, CH_3), 2.32 (s, 3H, CH_3), 2.20−2.10 (m, 2H, CH_2), 1.95 (d, J = 2 Hz, 3H, CH_3).

Isomer **5II**:

^{1}H NMR (500 MHz, $CDCl_3$, r.t., ppm): δ = 7.38−7.28 (m, 4H, CH), 7.23−6.98 (m, 4H, CH), 6.93−6.87 (m, 1H, CH), 6.85−6.79 (m, 1H, CH), 6.38 (dt, J = 6; 2 Hz, 1H, CH), 4.91 (td, J = 6; 2 Hz, 1H, $CH_{bridgehead}$), 4.60 (dt, J = 6; 2 Hz, 1H, $CH_{bridgehead}$), 3.11−2.99 (m, 4H, CH_2), 2.49−2.47 (s, 3H, CH_3), 2.32 (s, 3H, CH_3), 2.20−2.10 (m, 2H, CH_2), 1.86 (d, J = 2 Hz, 3H, CH_3).

Isomer **5III**:

^{1}H NMR (500 MHz, $CDCl_3$, r.t., ppm): δ = 7.38−7.28 (m, 4H, CH), 7.23−6.98 (m, 4H, CH), 6.85−6.79 (m, 2H, CH), 6.58 (dd, J = 6; 2 Hz, 2H, CH), 4.87 (tt, J = 6; 2 Hz, 1H, $CH_{bridgehead}$), 3.30 (t, J = 8 Hz, 2H, CH), 3.11−2.99 (m, 2H, CH_2), 2.49−2.47 (s, 3H, CH_3), 2.32 (s, 3H, CH_3), 2.20−2.10 (m, 2H, CH_2), 2.16 (s, 3H, CH_3).

Isomer **5IV**:

^{1}H NMR (500 MHz, $CDCl_3$, r.t., ppm): δ = 7.38−7.28 (m, 4H, CH), 7.23−6.98 (m, 4H, CH), 6.93−6.87 (m, 2H, CH), 6.48 (dd, J = 6; 2 Hz, 2H, CH), 4.95 (tt, J = 6; 2 Hz, 1H, $CH_{bridgehead}$), 3.11−2.99 (m, 4H, CH_2), 2.49−2.47 (s, 3H, CH_3), 2.30 (s, 3H, CH_3), 2.20−2.10 (m, 2H, CH_2), 1.36 (s, 3H, CH_3).

The chemical shift of the bridgehead H atoms at 5.0 to 4.5 ppm and their through-space coupling via NOESY are characteristic for each of the four isomers. Therefore, the assignment of the resonances to the four isomers will begin with those signals.

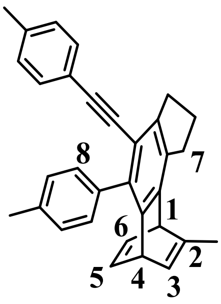

Figure S1: **Isomer I** of compound **5** including referencing numbers.

Isomer I has two bridgehead H atoms in positions **1** and **4**. The H atom in position **1** (4.71 ppm) shows NOESY cross-peaks to the CH_2 group of the five-membered ring in position **7** (3.11-2.99 ppm) and the CH_3 group, resulting from the trapped toluene molecule, in position **2** (1.95 ppm) (Fig. S5). The H atom in position **4** resonates at 4.84 ppm. This shows a NOESY cross-peak to the aromatic H atoms in position **8** (7.38-7.28 ppm) (Fig. S6). Furthermore, a NOESY cross-peak between the CH_3 group in position **2** (1.95 ppm) and the H atom in position **3** (6.28 ppm) was observed (Fig. S7). Continuing from the H atom in position **3** (6.28 ppm), a through-space coupling to the bridgehead H in position **4** (4.84 ppm) was detected (Fig. S8). With the help of the COSY-45 spectrum, the coupling of the H atom in position **3** (6.28 ppm) to the two bridgehead H atoms **1** (4.71 ppm) and **4** (4.84 ppm) was confirmed (Fig. S9). Lastly, the COSY-45 spectrum shows strong coupling of the bridgehead H atom in position **1** (4.71 ppm) with the H atom in position **6** (6.93-6.87 ppm) as well as strong coupling between the bridgehead H atom in position **4** (4.84 ppm) and the H atom in position **5** (6.85-6.79 ppm) (Fig. S9).

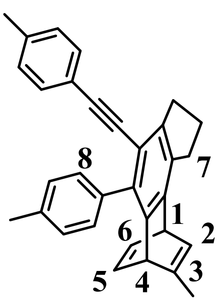

Figure S2: **Isomer II** of compound **5** including referencing numbers.

Isomer II has two bridgehead H atoms in positions **1** and **4**. The H atom in position **1** (4.91 ppm) shows a NOESY cross-peak to the CH_2 group of the five-membered ring in position **7** (3.11-2.99 ppm) (Fig. S5). The H atom in position **4** resonates at

4.60 ppm, showing a NOESY cross-peak to the CH_3 group, resulting from the trapped toluene molecule, in position **3** (1.86 ppm) and the aromatic H atoms in position **8** (7.38-7.28 ppm) (Fig. S6). Furthermore, a NOESY cross-peak between the CH_3 group in position **3** (1.86 ppm) and the H atom in position **2** (6.38 ppm) was observed (Fig. S7). Continuing from the H atom in position **2** (6.38 ppm), a through-space coupling to the bridgehead H in position **1** (4.91 ppm) was detected (Fig. S8). With the help of the COSY-45 spectrum, the coupling of the H atom in position **2** (6.38 ppm) to the two bridgehead H atoms **1** (4.91 ppm) and **4** (4.60 ppm) was confirmed (Fig. S9). Lastly, the COSY-45 spectrum shows strong coupling of the bridgehead H atom in position **1** (4.91 ppm) with the H atom in position **6** (6.93-6.87 ppm) as well as coupling between the bridgehead H atom in position **4** (4.60 ppm) and the H atom in position **5** (6.85-6.79 ppm) (Fig. S9).

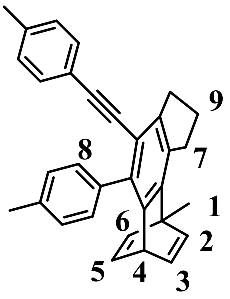

Figure S3: **Isomer III** of compound **5** including referencing numbers.

Isomer III has one bridgehead H atom in position **4**, which resonates at 4.87 ppm, showing a NOESY cross-peak to the aromatic H atoms in position **8** (7.38-7.28 ppm) (Fig. S6). Furthermore, a NOESY cross-peak between the CH_3 group in position **1** (2.20-2.10 ppm) and the H atoms in position **2** and **6** (6.58 ppm) was observed (Fig. S7). The cross-peak expected for the coupling of the CH_3 group in position **1** (2.20-2.10 ppm) and the CH_2 group in position **7** (3.11-2.99 ppm) overlaps with the cross-peaks of the CH_2 groups in positions **7** and **9** (2.20-2.10 ppm). With the help of the COSY-45 spectrum, the coupling of the H atoms in positions **3** and **5** (6.85-6.79 ppm) to the bridgehead H atom in position **4** (4.87 ppm) was confirmed (Fig. S9). Lastly, the COSY-45 spectrum shows coupling of the bridgehead H atom in position **4** (4.87 ppm) with the H atoms in position **2** and **6** (6.58 ppm) (Fig. S9).

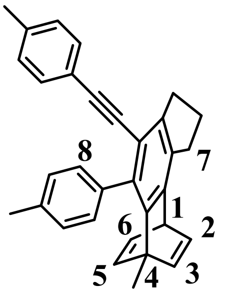

Figure S4: **Isomer IV** of compound **5** including referencing numbers.

Isomer IV has one bridgehead H atom in position **1**, which resonates at 4.95 ppm, showing a NOESY cross-peak to the CH_2 group of the five-membered ring in position **7** (3.11-2.99 ppm) (Fig. S5). Furthermore, a NOESY cross-peak between the CH_3 group in position **4** (1.36 ppm) and the H atoms in position **3** and **5** (6.48 ppm) was observed (Fig. S7). With the help of the COSY-45 spectrum, the coupling of the H atoms in position **2** and **6** (6.93-6.87 ppm) to the bridgehead H atom in position **1** (4.95 ppm) was confirmed (Fig. S9). Lastly, the COSY-45 spectrum shows coupling of the bridgehead H atom in position **1** (4.95 ppm) with the H atoms in position **3** and **5** (6.48 ppm) (Fig. S9).

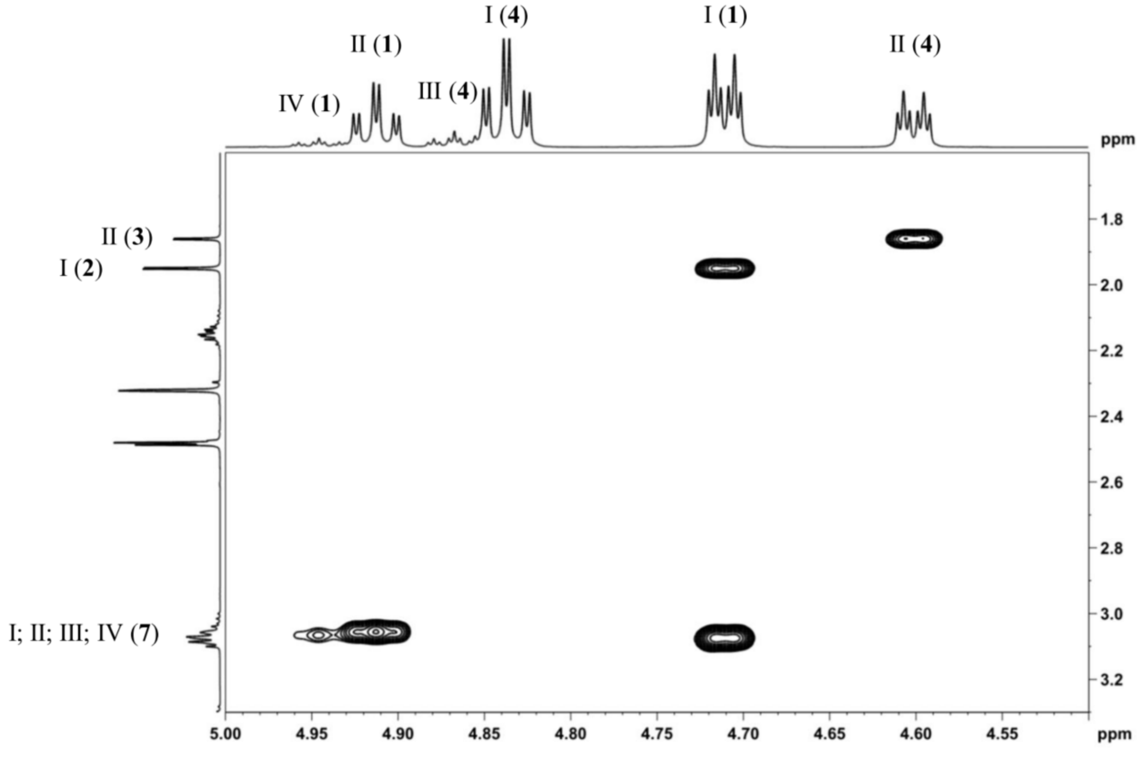

Figure S5: ^{1}H NOESY spectrum, 500 MHz ($CDCl_3$).

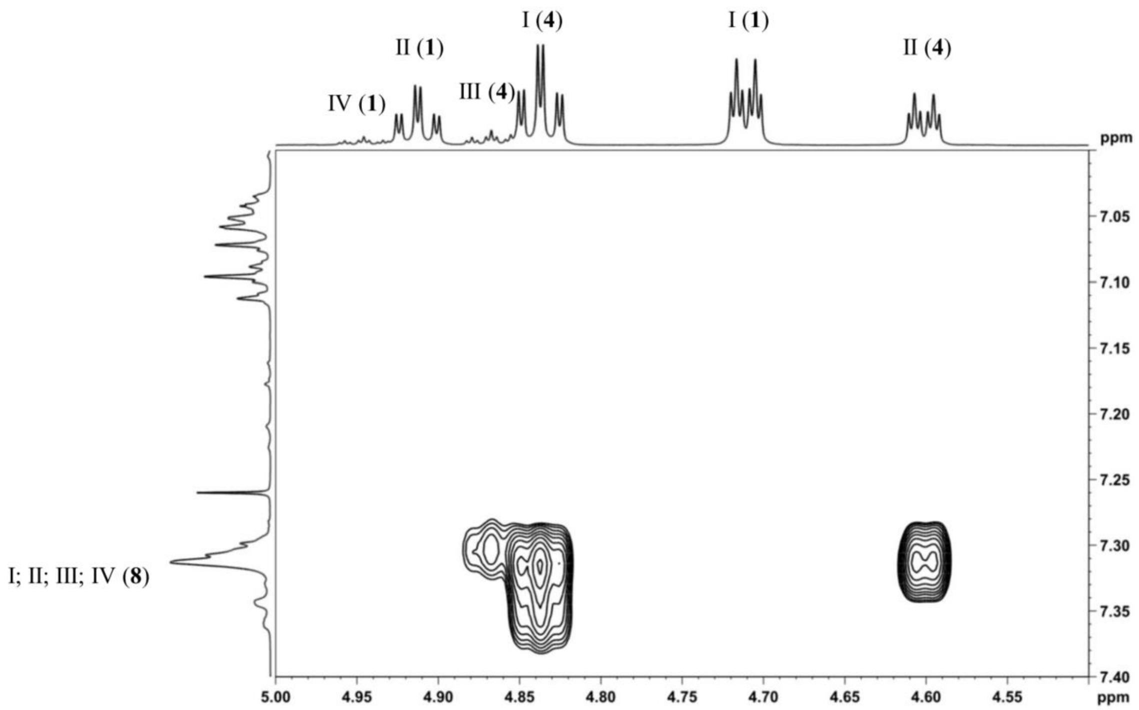

Figure S6: ^{1}H NOESY spectrum, 500 MHz ($CDCl_3$).

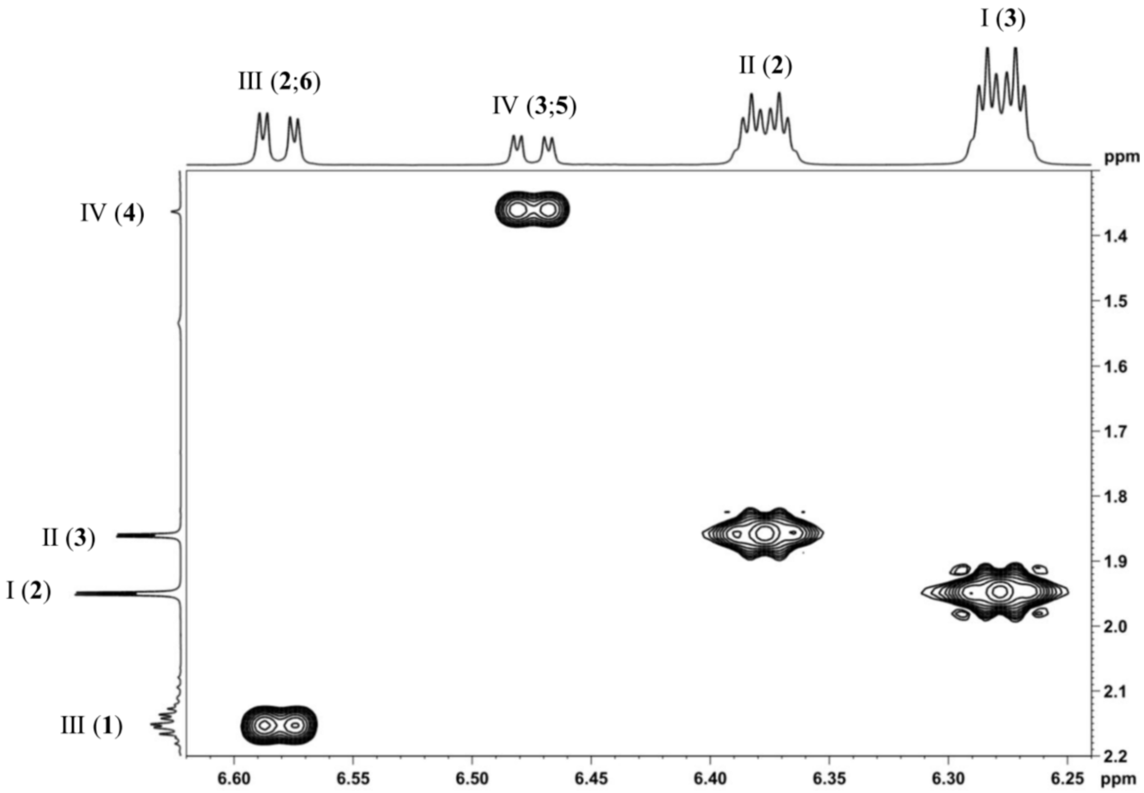

Figure S7: ^{1}H NOESY spectrum, 500 MHz ($CDCl_3$).

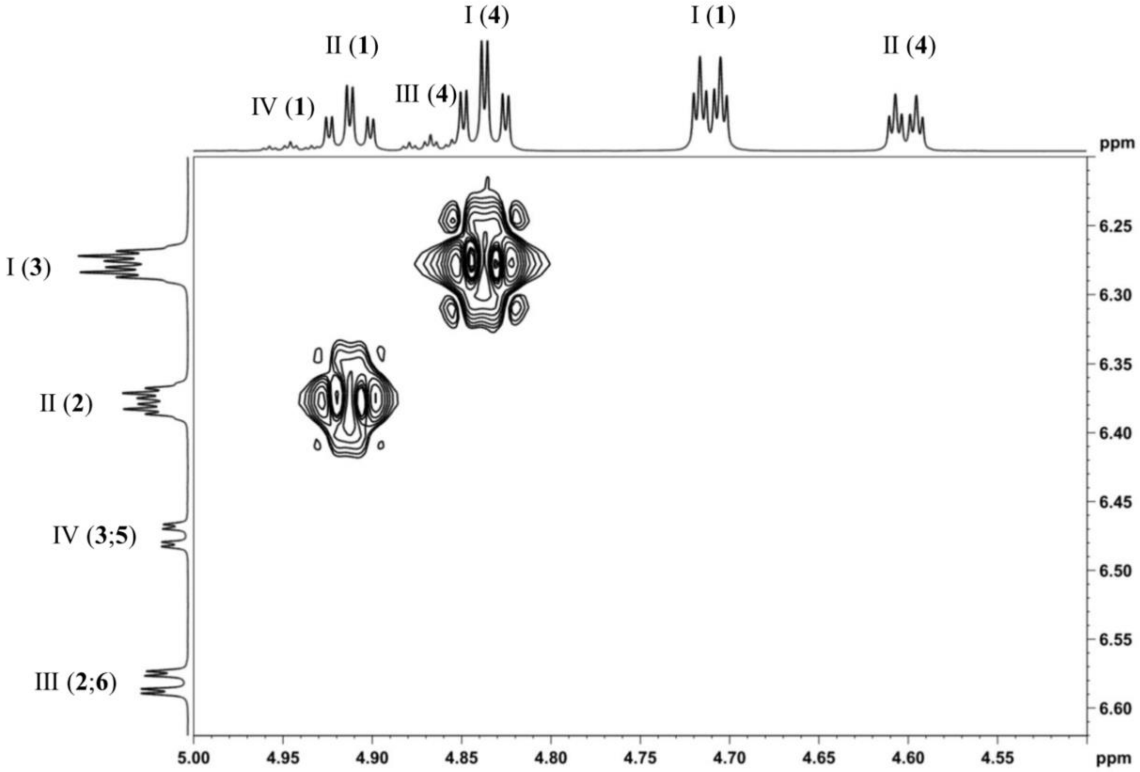

Figure S8: ^{1}H NOESY spectrum, 500 MHz ($CDCl_3$).

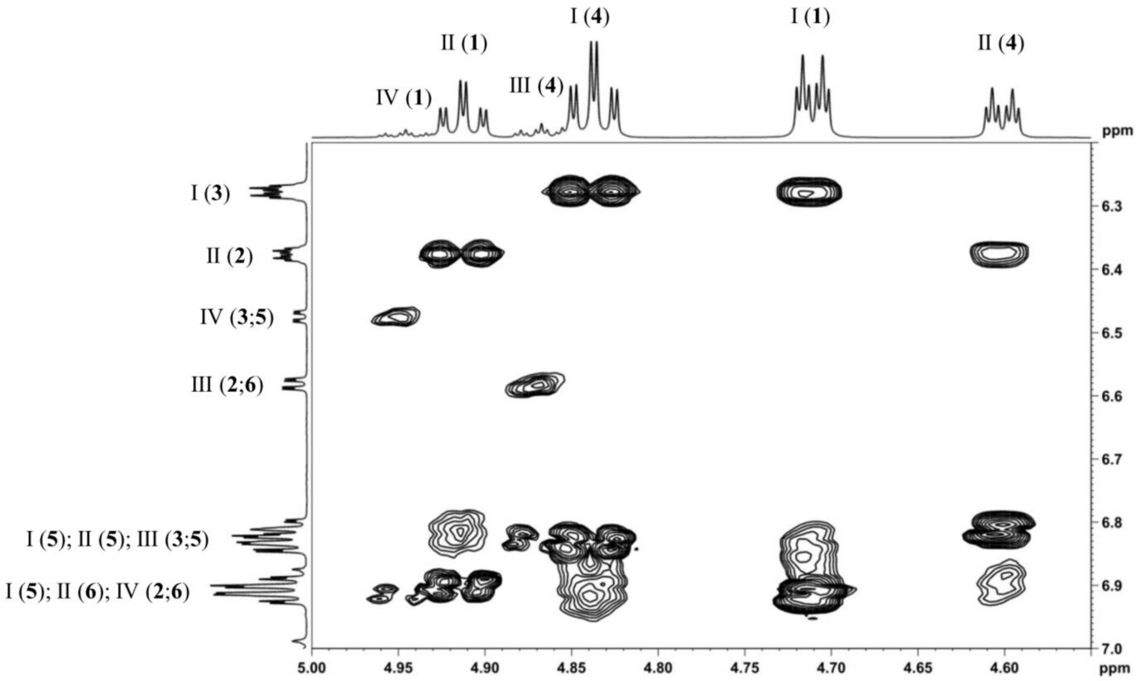

Figure S9: ^{1}H COSY-45 spectrum, 500 MHz ($CDCl_3$).

Synthesis of benzene adduct 6

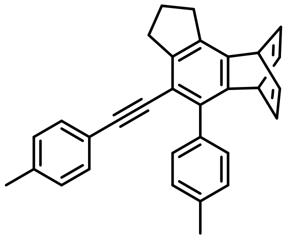

The compound 1,11-bis(*p*-tolyl)undeca-1,3,8,10-tetrayne **3** (0.50 g, 1.56 mmol) was dissolved in 125 mL of degassed benzene in a Young's tap tube which was then sealed with a teflon tap and stirred at 110 °C for 4 d under an argon atmosphere. The solvent was evaporated, and the dark yellow solid was re-dissolved in CH_2Cl_2 and rotary-evaporated onto silica. Following flash chromatography, using hexane/CH_2Cl_2 (0% to 25% CH_2Cl_2) as eluent, the solvent from the third fraction was evaporated and the crude product was recrystallized by diffusion of hexane into CH_2Cl_2, giving **6** as colorless crystals. Yield: 0.10 g (0.26 mmol, 16%)

^{1}H NMR (500 MHz, $CDCl_3$, r.t., ppm): δ = 7.32–7.29 (m, 4H, CH), 7.10–7.03 (m, 4H, CH), 6.92–6.89 (m, 2H, CH), 6.83–6.81 (m, 2H, CH), 5.07–5.04 (tt, J = 9; 2 Hz, 1H, $CH_{bridgehead}$), 4.99–4.96 (tt, J = 9; 2 Hz, 1H, $CH_{bridgehead}$), 3.07 (t, J = 7 Hz, 2H, CH_2), 3.06 (t, J = 7 Hz, 2H, CH_2), 2.48 (s, 3H, CH_3), 2.31 (s, 3H, CH_3), 2.14 (quin., J = 7 Hz, 2H, CH_2).

^{13}C{^{1}H} NMR (125 MHz, $CDCl_3$, r.t. ppm): δ = 143.6, 143.2, 143.1, 140.2, 139.5, 137.7, 136.6, 136.3, 136.1, 136.0, 131.2, 130.4, 129.0, 128.5, 121.2, 113.9, 94.3, 88.2, 46.9, 46.7, 33.3, 31.0, 24.9, 21.6, 21.5.

Elem. Anal. Calc. (%) for $C_{31}H_{26}$: C 93.42; H 6.58; found: C 93.39; H 6.75.

HRMS (APCI$^+$): [$C_{31}H_{26}$+H^+] calc.: m/z = 399.2107; found: m/z = 399.2099 (|Δ| = 2.00 ppm)

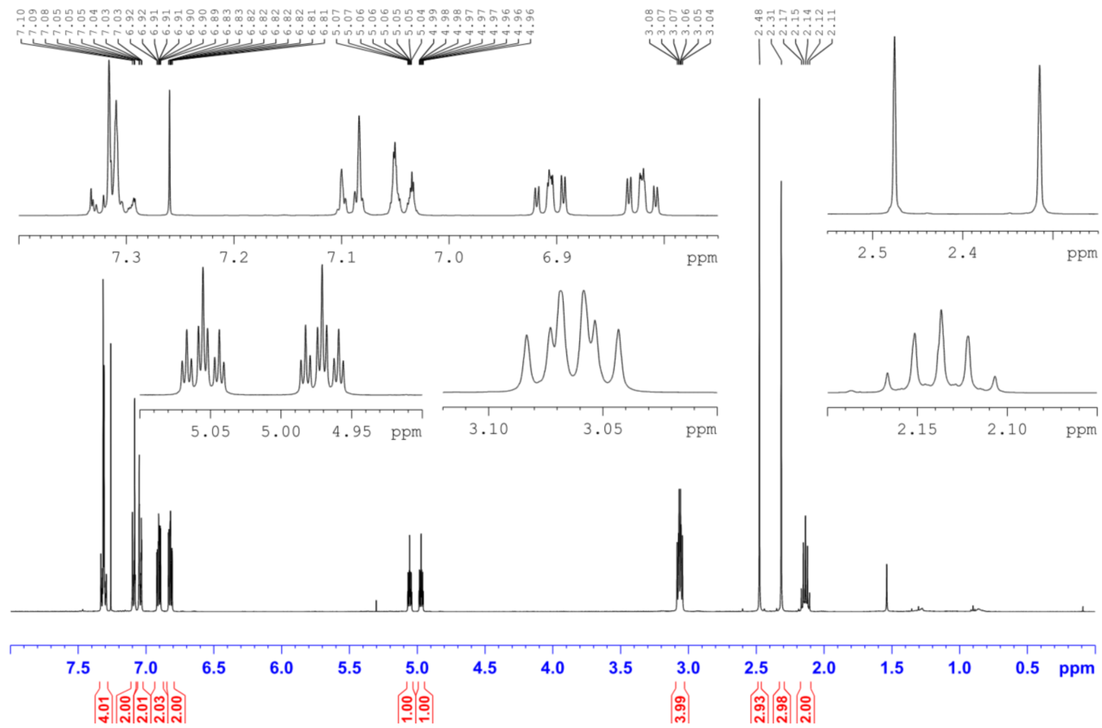

^{1}H NMR spectrum, 500 MHz ($CDCl_3$).

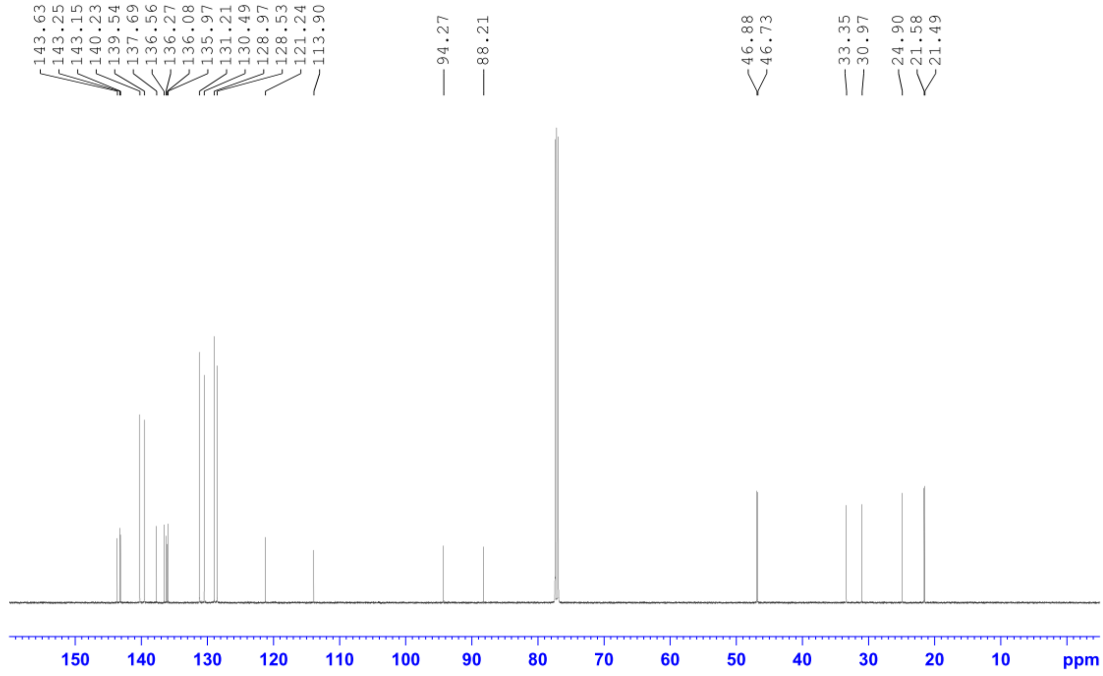

$^{13}C\{^{1}H\}$ NMR spectrum, 125 MHz ($CDCl_3$).

Synthesis of 8 and 9

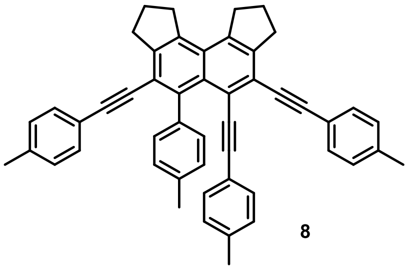
8

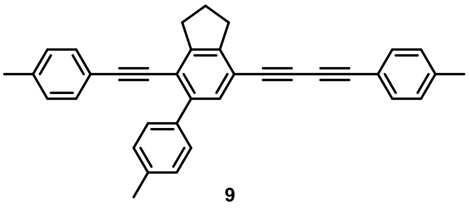
9

In an argon-filled glovebox, a solution of 1,11-bis(*p*-tolyl)undeca-1,3,8,10-tetrayne (**3**) (0.50 g, 1.56 mmol) in toluene (18 mL) was prepared in a sealable microwave vial equipped with magnetic stirring bar. The vial was subsequently crimp sealed and heated with stirring to 110 °C for 4 d, after which the mixture was cooled to room temperature and all volatiles were removed *in vacuo*. The resulting residue was purified by flash chromatography, using a gradient of hexane/CH_2Cl_2 (0% to 20% CH_2Cl_2) as eluent. Compounds **8** (156 mg, 31%) and **9** (26 mg, 8%) were isolated as yellowish and colorless solids, respectively.

8: **^{1}H NMR** (500 MHz, $CDCl_3$, r.t., ppm): δ = 7.37–7.35 (m, 4H, CH), 7.21–7.19 (m, 2H, CH), 7.09–6.97 (m, 10H, CH), 3.61 (t, J = 7 Hz, 2H, CH_2), 3.61 (t, J = 7 Hz, 2H, CH_2), 3.25–3.20 (m, 4H, CH_2), 2.34 (s, 6H, CH_3), 2.32 (s, 6H, CH_3), 2.25–2.20 (m, 4H, CH_2).

^{13}C{^{1}H} NMR (125 MHz, $CDCl_3$, r.t. ppm): δ = 143.9, 143.7, 142.6, 139.6, 139.4, 138.5, 138.4, 138.1, 137.4, 136.9, 131.8, 131.7, 131.5, 131.4, 130.7, 129.2, 129.0, 128.9, 128.5, 128.2, 125.3, 122.7, 121.3, 120.9, 120.8, 120.6, 103.8, 97.7, 97.1, 89.6, 88.6, 88.4, 36.2, 36.0, 33.7 (2C), 24.3, 24.2, 21.66, 21.63, 21.60, 21.5.

Elem. Anal. Calc. (%) for $C_{50}H_{40}$: C 93.71, H 6.29; found: C 93.23, H 6.41.

HRMS (APCI$^+$): [$C_{50}H_{40}$ + H^+] calc.: m/z = 641.3203; found: m/z = 641.3196 (|Δ| = 1.09 ppm).

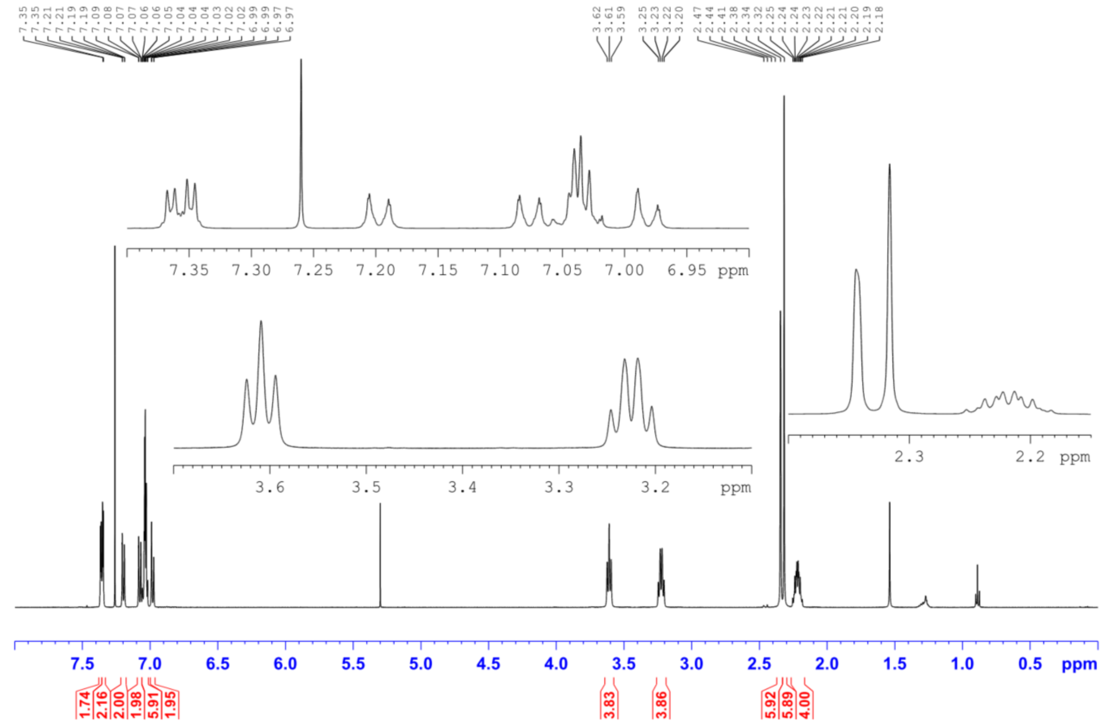

^{1}H NMR spectrum, 500 MHz ($CDCl_3$).

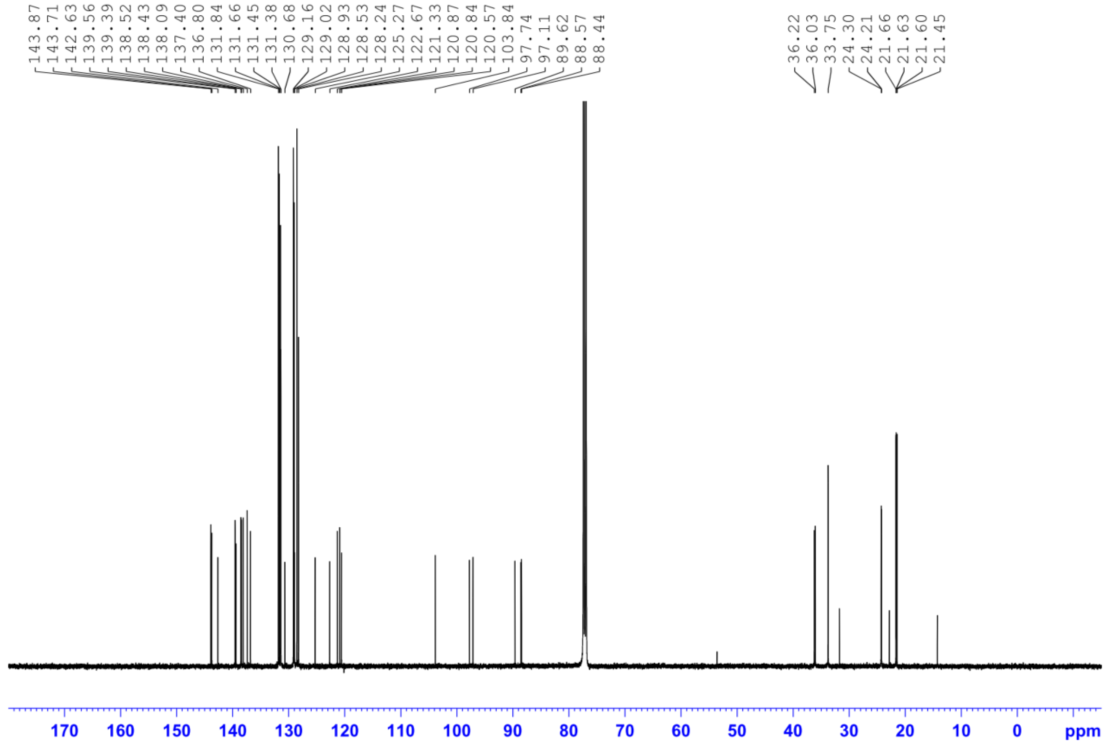

^{13}C{^{1}H} NMR spectrum, 125 MHz ($CDCl_3$).

9: **^{1}H NMR** (500 MHz, $CDCl_3$, r.t., ppm): δ = 7.55 (m, 2H, CH), 7.43 (m, 2H, CH), 7.38 (s, 1H, CH), 7.26–7.23 (m, 4H, CH), 7.15 (m, 2H, CH), 7.11 (m, 2H, CH), 3.16 (t, J = 8 Hz, 2H, CH_2), 3.13 (t, J = 8 Hz, 2H, CH_2), 2.42 (s, 3H, CH_3), 2.37 (s, 3H, CH_3), 2.35 (s, 3H, CH_3), 2.18 (quin., J = 8 Hz, CH_2).

^{13}C{^{1}H} NMR (125 MHz, $CDCl_3$, r.t. ppm): δ = 148.0, 146.4, 141.8, 139.8, 138.6, 137.3, 137.1, 132.6, 131.5, 131.4, 129.4, 129.3, 129.2, 128.8, 120.6, 119.3, 118.9, 117.7, 97.7, 87.4, 83.1, 80.2, 77.8, 73.7, 33.8, 33.1, 24.3, 21.8, 21.7, 21.4.

Elem. Anal. Calc. (%) for $C_{36}H_{28}$: C 93.87, H 6.13; found: C 93.97, H 6.02.

HRMS (APCI$^+$) [$C_{36}H_{28}$ + H^+] calc.: m/z = 461.2264; found: m/z = 461.2259 (|Δ| = 1.08 ppm)

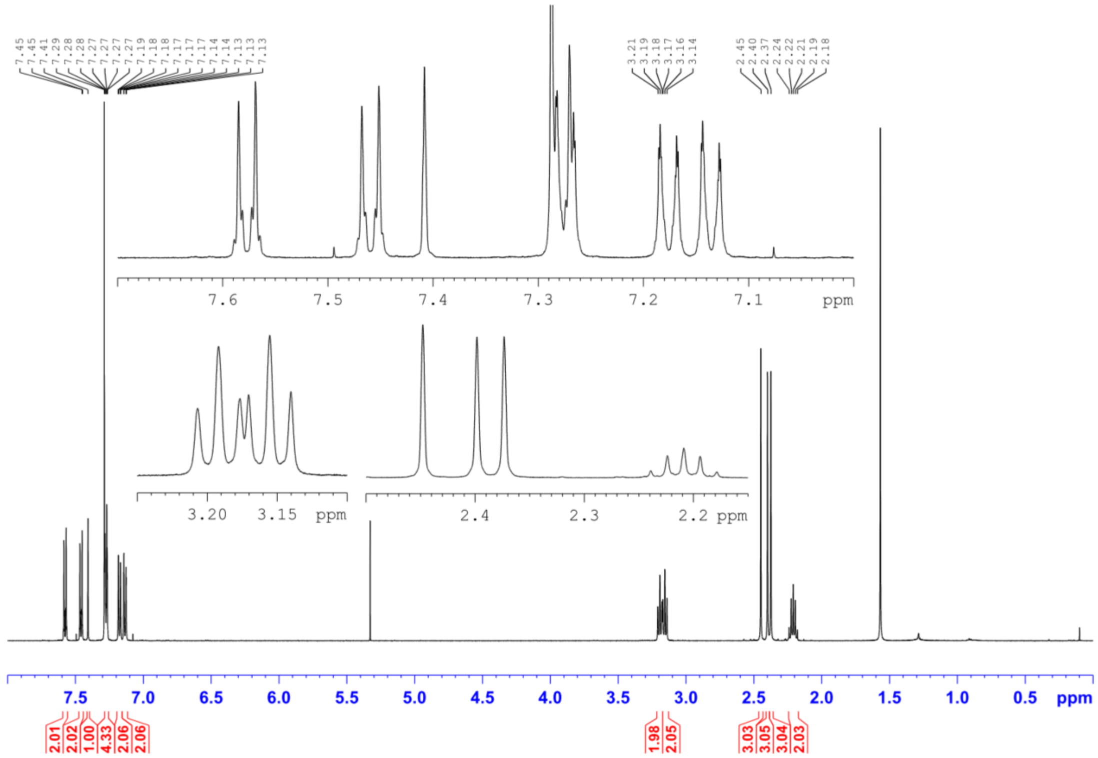

^{1}H NMR spectrum, 500 MHz ($CDCl_3$).

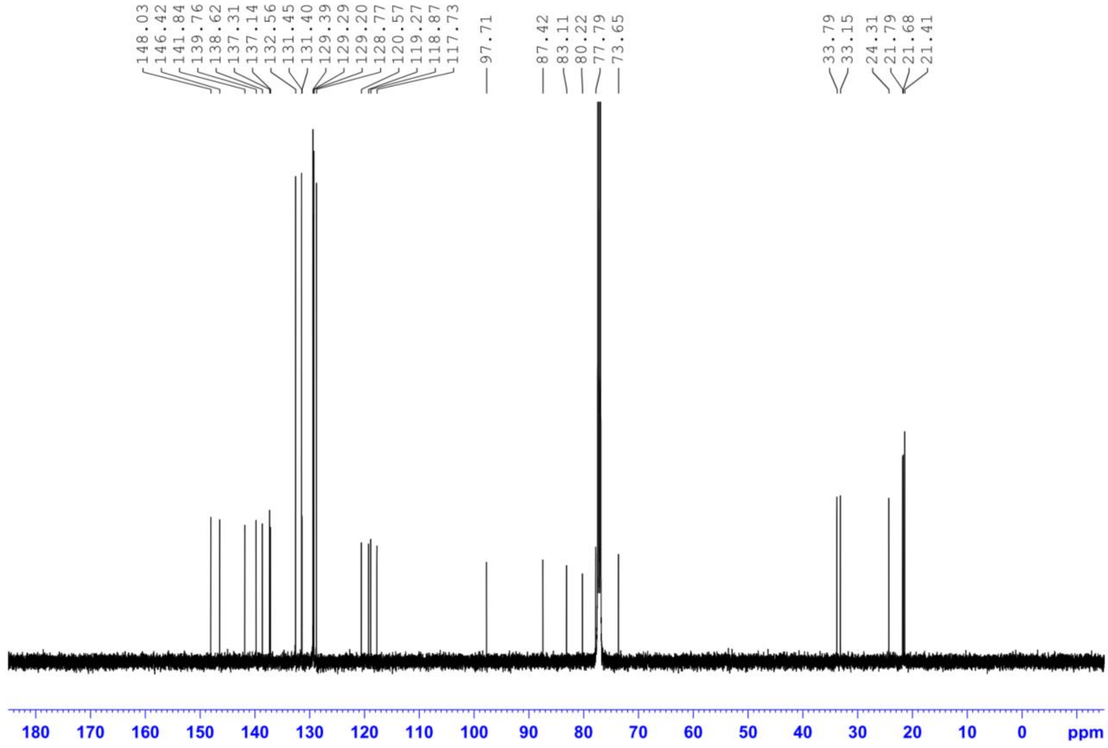

$^{13}C\{^{1}H\}$ NMR spectrum, 125 MHz ($CDCl_3$).

Synthesis of 10

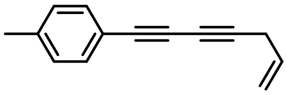

The compound 1,11-bis(*p*-tolyl)undeca-1,3,8,10-tetrayne **3** (0.50 g, 1.56 mmol) was dissolved in 125 mL of degassed toluene and stirred at 110 °C for 4 d under an argon atmosphere. The solvent was evaporated, and the dark yellow solid was re-dissolved in CH_2Cl_2 and rotary-evaporated onto silica. Flash column chromatography, using hexane/CH_2Cl_2 (0% to 20% CH_2Cl_2) as eluent, gave **10** as a colorless solid. Crude yield: 7.0 mg (0.04 mmol, 2.5%)

^{1}H NMR (300 MHz, $CDCl_3$, r.t., ppm): δ = 7.40−7.36 (m, 2H, CH), 7.13−7.09 (m, 2H, CH), 5.88−5.75 (ddt, J = 17; 10; 5 Hz, 1H, CH), 5.40−5.33 (ddt, J = 17; 2; 2 Hz, 1H, CH), 5.20−5.15 (ddt, J = 10; 2; 2 Hz, 1H, CH), 3.15−3.13 (ddd, J = 5; 2; 2 Hz, 2H, CH_2), 2.35 (s, 3H, CH_3).

$^{13}C\{^1H\}$ NMR (75 MHz, $CDCl_3$, r.t. ppm): δ = 139.4, 132.6, 131.3, 129.3, 118.9, 117.2, 80.6, 76.0, 73.6, 67.5, 24.0, 21.7.

HRMS (APCI$^+$): [$C_{14}H_{12}$+H^+] calc.: m/z = 181.1012; found: m/z = 181.1009 (|Δ| = 1.66 ppm)

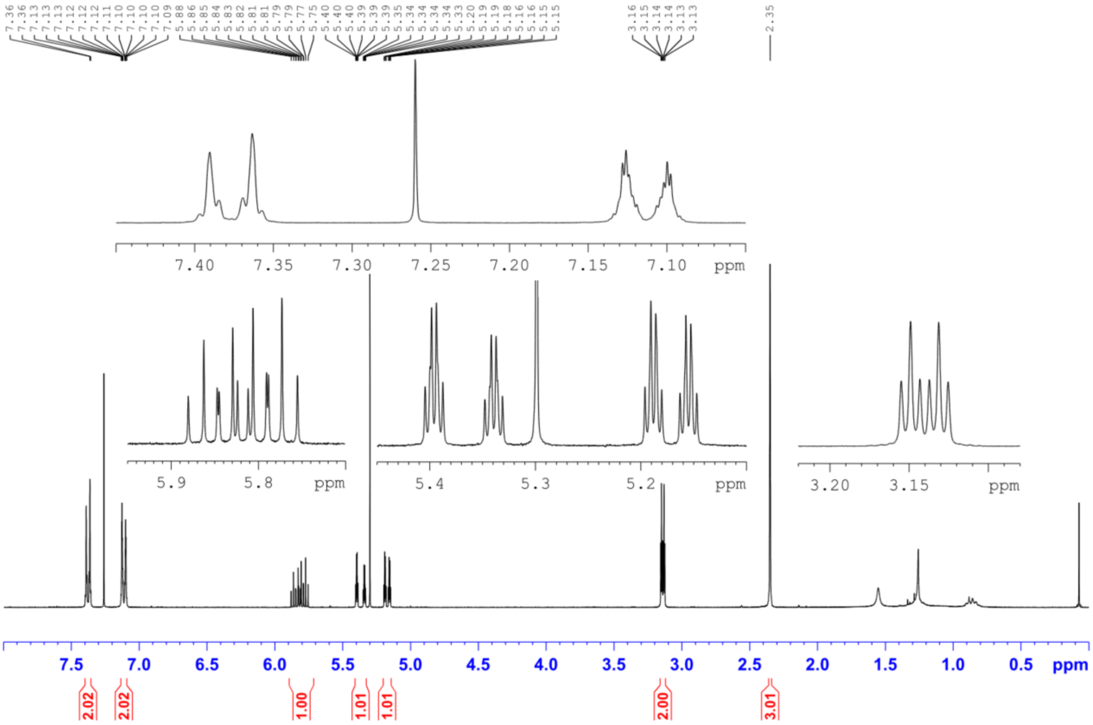

^1H NMR spectrum, 300 MHz ($CDCl_3$).

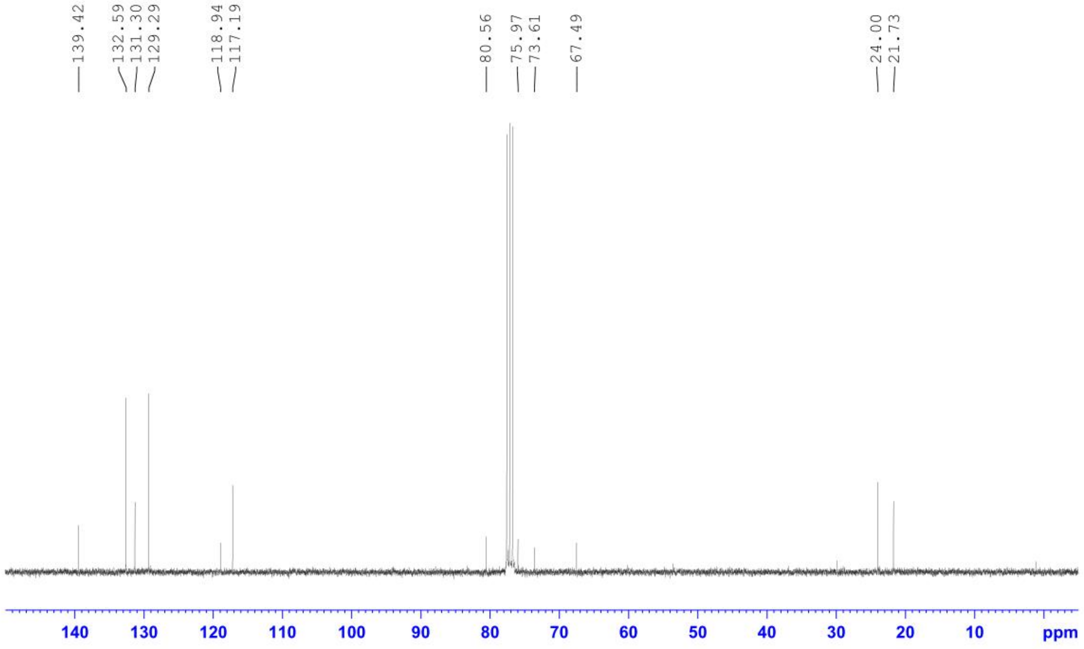

$^{13}C\{^1H\}$ NMR spectrum, 75 MHz ($CDCl_3$).

Synthesis of pyrene derivative 11

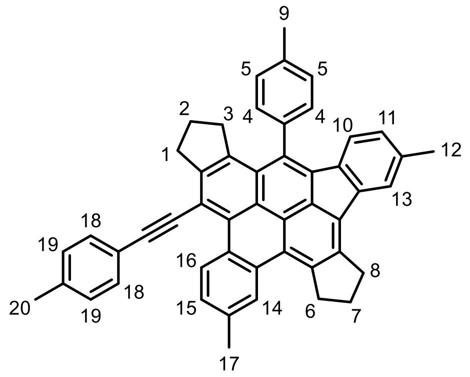

The compound 1,11-bis(*p*-tolyl)undeca-1,3,8,10-tetrayne **3** (3.00 g, 9.36 mmol) was dissolved in 130 mL of benzene and stirred at 110 °C for 7 d. The solvent was removed *in vacuo* and the remaining yellow solid was re-dissolved in CH_2Cl_2 and rotary-evaporated onto silica. The compound was purified twice by flash chromatography with a solvent system composed of hexane/CH_2Cl_2 (0% to 5% CH_2Cl_2). All volatiles were removed *in vacuo*. The yellow solid was recrystallized by diffusion of hexane into CH_2Cl_2. Yield: 70 mg (0.11 mmol, 2%)

^{1}H NMR (500 MHz, $CDCl_3$, r.t., ppm): δ = 10.29 (d, J = 9, 1H, C*H*-16), 8.65 (s, 1H, C*H*-14), 7.72 (m, 1H, C*H*-13), 7.57 (m, 2H, C*H*-18), 7.52 (m, 1H, C*H*-15), 7.44 (m, 2H, C*H*-4), 7.38 (m, 2H, C*H*-5), 7.24 (m, 2H, C*H*-19), 6.86 (m, 1H, C*H*-11), 6.43 (d, J = 9, 1H, C*H*-10), 3.92 (t, J = 7 Hz, 2H, CH_2-6), 3.61 (t, J = 8 Hz, 2H, CH_2-8), 3.42 (t, J = 8 Hz, 2H, CH_2-1), 2.74 (t, J = 8 Hz, 2H, CH_2-3), 2.67 (s, 3H, CH_3-17), 2.59 (s, 3H, CH_3-9), 2.45 (s, 3H, CH_3-12), 2.45 (m, 2H, CH_2-7), 2.42 (s, 3H, CH_3-20), 2.00 (quint., J = 8 Hz, 2H, CH_2-2).

^{13}C{^{1}H} NMR (125 MHz, $CDCl_3$, r.t. ppm): δ = 148.1 (C_q), 141.9 (C_q), 141.6 (C_q), 140.3 (C_q), 139.94 (C_q), 138.7 (C_q), 138.6 (C_q), 137.63 (C_q), 137.61 (C_q), 137.1 (C_q), 136.6 (C_q), 135.7 (C_q), 134.5 (C_q), 132.4 (C_q), 131.4 (^{Ar}CH), 130.0 (^{Ar}CH), 129.8 (C_q), 129.56 (C_q), 129.49 (C_q), 129.44 (^{Ar}CH), 129.41 (C_q), 129.23 (^{Ar}CH), 128.8 (C_q), 128.2 (^{Ar}CH), 127.2 (^{Ar}CH), 126.95 (^{Ar}CH), 126.85 (^{Ar}CH), 126.3 (C_q), 125.1 (C_q), 124.3 (^{Ar}CH), 123.4 (^{Ar}CH), 121.8 (C_q), 121.3 (C_q), 114.4 (C_q), 100.4 (C_q), 91.3 (C_q), 37.4 (CH_2), 36.7 (CH_2), 34.7 (CH_2), 31.6 (CH_2), 26.9 (CH_2), 24.7 (CH_2), 22.2 (CH_3), 22.0 (CH_3), 21.77 (CH_3), 21.76 (CH_3).

HRMS (APCI+): [$C_{50}H_{38}$ +H^+] calc.: m/z = 639.3007; found: m/z = 639.3021 (|Δ| = 2.19 ppm).

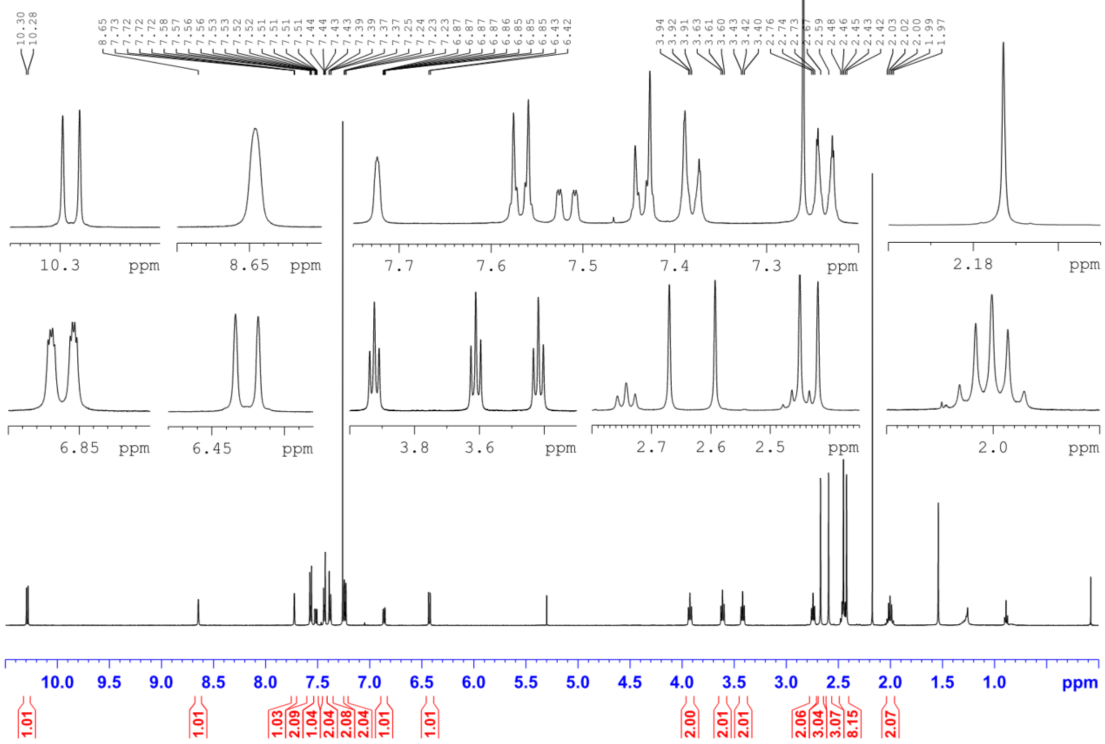

^{1}H NMR spectrum, 500 MHz ($CDCl_3$).

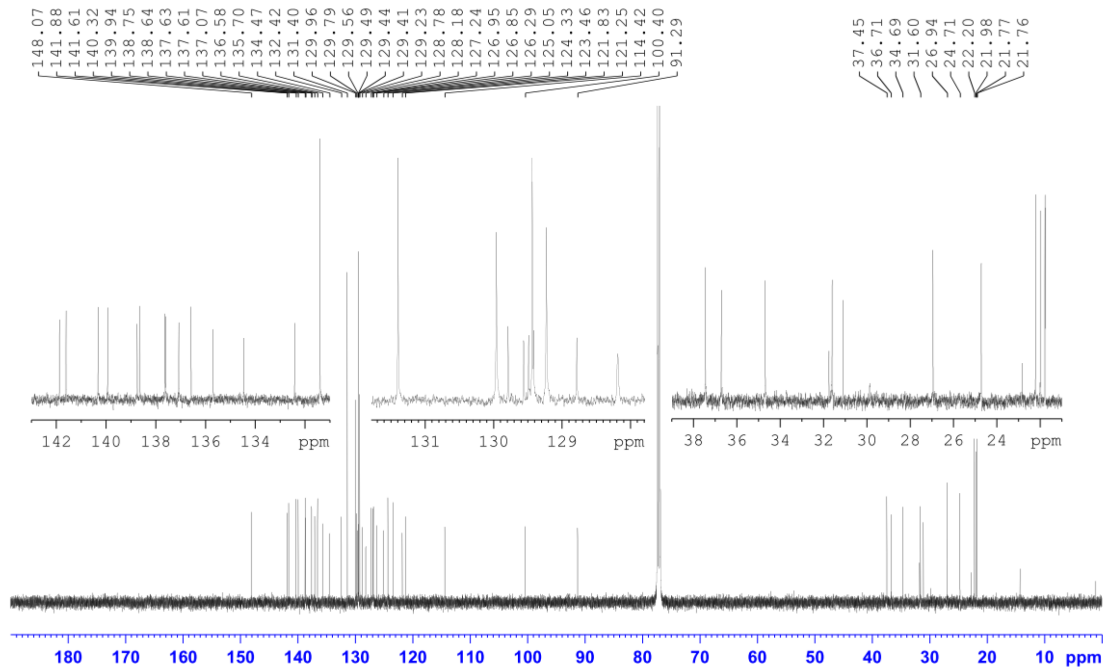

^{13}C{^{1}H} NMR spectrum, 125 MHz ($CDCl_3$).

Photophysical measurements

General photophysical measurements. All measurements were performed in standard quartz cuvettes (1 cm x 1 cm cross-section). UV-visible absorption spectra were recorded using an Agilent 8453 diode array UV-visible spectrophotometer. The emission spectra were recorded using an Edinburgh Instruments FLSP920 spectrometer equipped with a double monochromator for both excitation and emission, operating in right-angle geometry mode, and all spectra were fully corrected for the spectral response of the instrument. All solutions used in photophysical measurements had concentrations lower than $5 \cdot 10^{-6}$ M to minimize inner filter effects during fluorescence measurements.

Fluorescence quantum yield measurements. The fluorescence quantum yields were measured using a calibrated integrating sphere (inner diameter: 150 mm) from Edinburgh Instruments combined with the FLSP920 spectrometer described above. For solution-state measurements, the longest-wavelength absorption maximum of the compound in the respective solvent was chosen as the excitation wavelength, unless stated otherwise.

Lifetime measurements. Fluorescence lifetimes were recorded using the time-correlated single-photon counting (TCSPC) method using an Edinburgh Instruments FLS980 spectrometer equipped with a high speed photomultiplier tube positioned after a single emission monochromator. Measurements were made in right-angle geometry mode, and the emission was collected through a polarizer set to the magic angle. Solutions were excited with a pulsed diode laser at a wavelength of 316 nm (for **4**, **6**, **8**, **9**) and 472 nm (for **11**). The full-width-at-half-maximum (FWHM) of the pulse from the diode laser was ca. 75-90 ps with an instrument response function (IRF) of ca. 230 ps FWHM. The IRFs were measured from the scatter of an aqueous suspension of Ludox at the excitation wavelength. Decays were recorded to 10000 counts in the peak channel with a record length of 8192 channels. The band pass of the emission monochromator and a variable neutral density filter on the excitation side were adjusted to give a signal count rate of <60 kHz. Iterative reconvolution of the IRF with one decay function and non-linear least-squares

analysis were used to analyze the data. The quality of all decay fits was judged to be satisfactory, based on the calculated values of the reduced χ^2 and Durbin-Watson parameters and visual inspection of the weighted residuals.

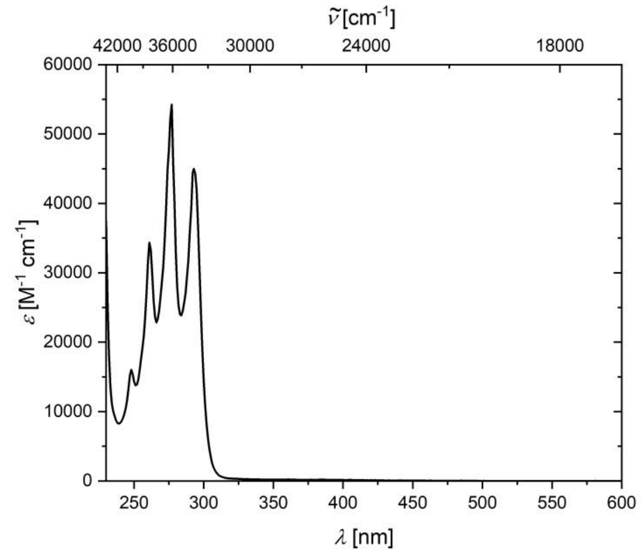

UV/Vis absorption spectrum of 3 in THF

Quantum yield and lifetime were not measured for compound **5** because of the mixture of four isomers (see text). Absorption between 350 and 450 nm is due to trace quantities of **11**. The excitation spectrum (λ_{em} = 352 nm) shows no band between 350 and 450 nm, because compound **11** does not emit at 352 nm.

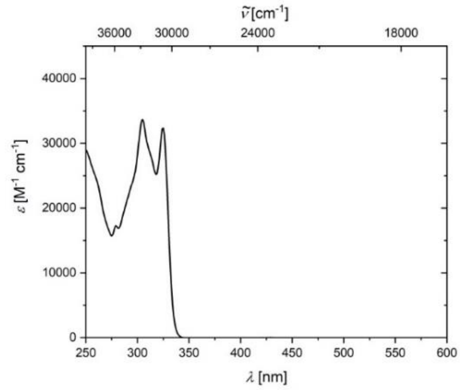

UV/Vis absorption spectrum of 4 in CH_2Cl_2

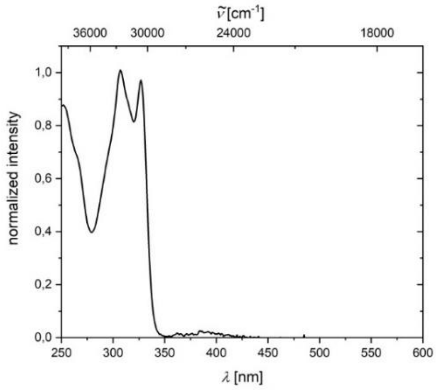

UV/Vis absorption spectrum of 5 in CH_2Cl_2

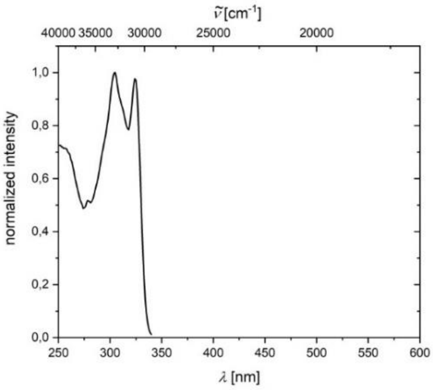

Excitation spectrum of 4 in CH_2Cl_2 (λ_{em} = 350 nm)

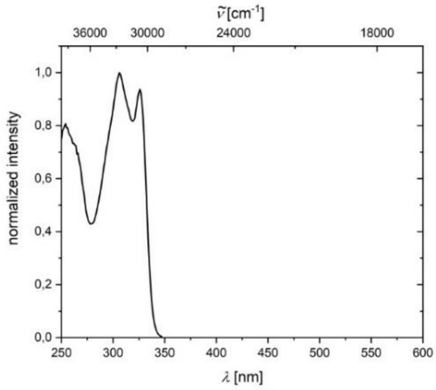

Excitation spectrum of 5 in CH_2Cl_2 (λ_{em} = 352 nm)

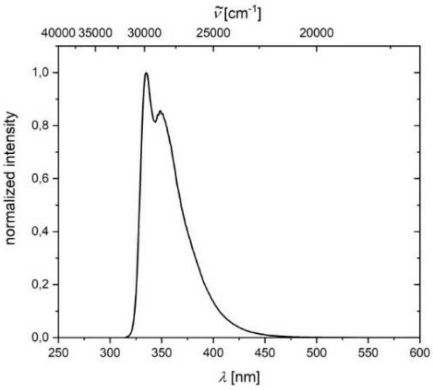

Emission spectrum of 4 in CH_2Cl_2 (λ_{ex} = 305 nm)

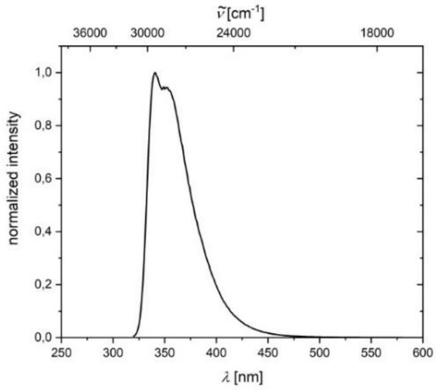

Emission spectrum of 5 in CH_2Cl_2 (λ_{ex} = 307 nm)

Quantum yield: 54%

Lifetime 1.1 ns

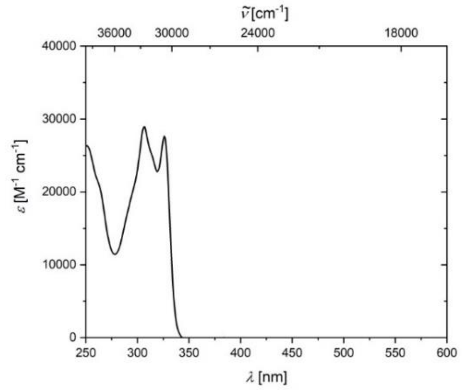

UV/Vis absorption spectrum of 6 in CH_2Cl_2

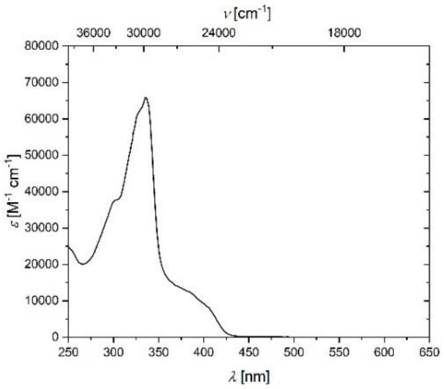

UV/Vis absorption spectrum of 8 in CH_2Cl_2

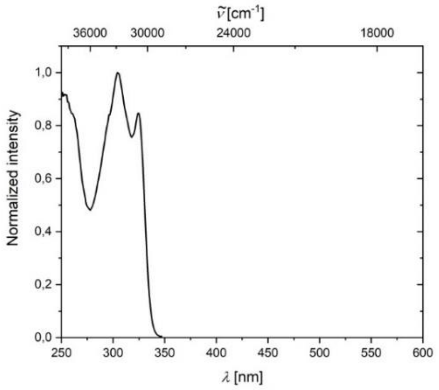

Excitation spectrum of 6 in CH_2Cl_2 (λ_{em} = 352 nm)

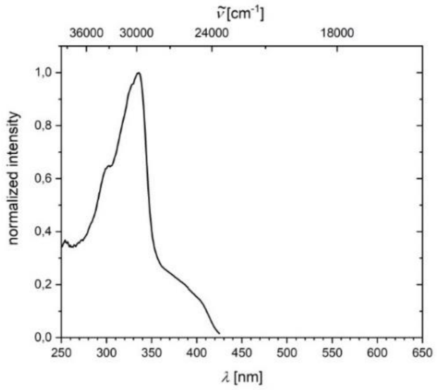

Excitation spectrum of 8 in CH_2Cl_2 (λ_{em} = 437 nm)

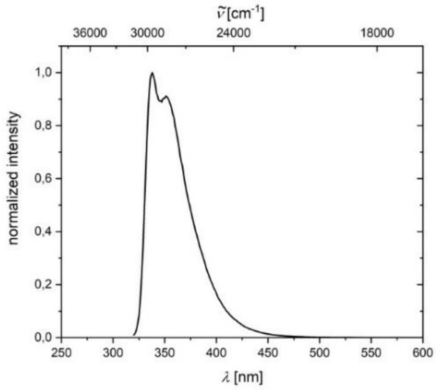

Emission spectrum of 6 in CH_2Cl_2 (λ_{ex} = 307 nm)

Quantum yield: 5%

Lifetime: < 1 ns

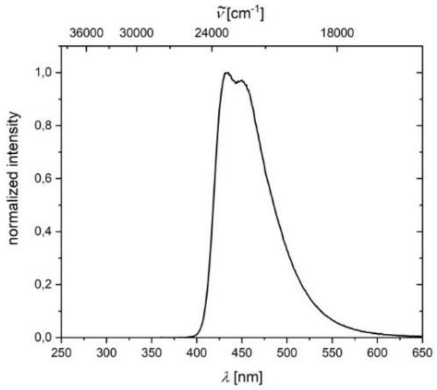

Emission spectrum of 8 in CH_2Cl_2 (λ_{ex} = 350 nm)

Quantum yield: 21%

Lifetime: 3.9 ns

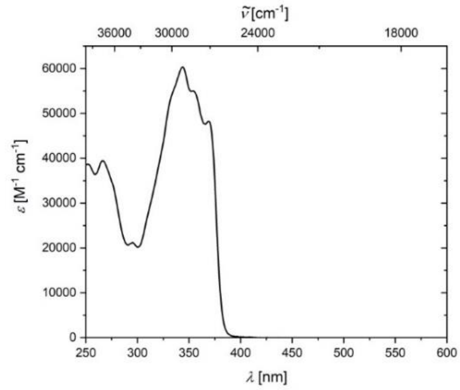

UV/Vis absorption spectrum of 9 in CH_2Cl_2

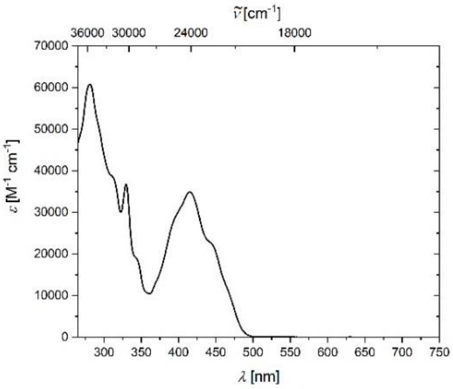

UV/Vis absorption spectrum of 11 in CH_2Cl_2

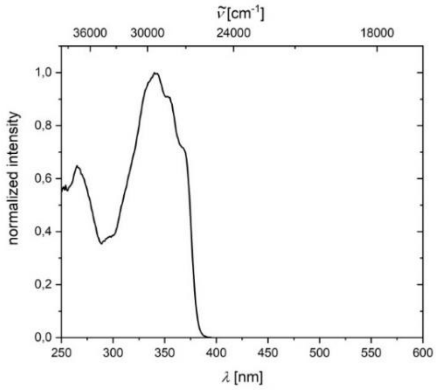

Excitation spectrum of 9 in CH_2Cl_2 (λ_{em} = 400 nm)

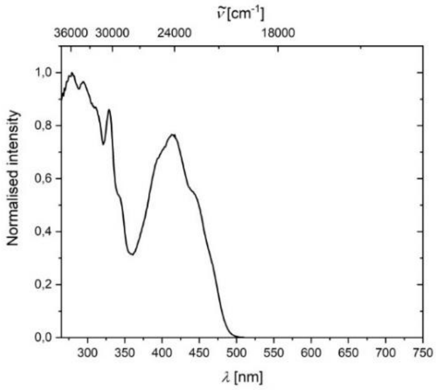

Excitation spectrum of 11 in CH_2Cl_2 (λ_{em} = 524 nm)

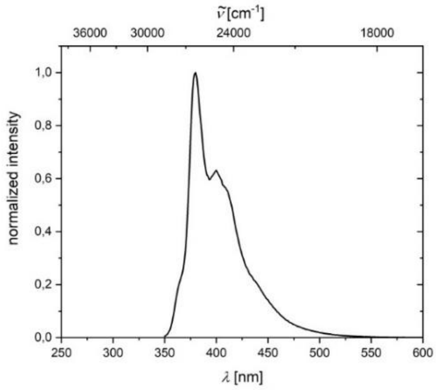

Emission spectrum of 9 in CH_2Cl_2 (λ_{ex} = 344 nm)

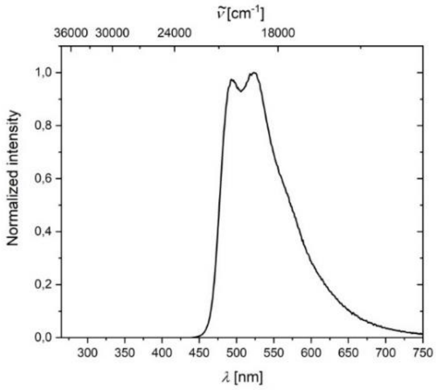

Emission spectrum of 11 in CH_2Cl_2 (λ_{ex} = 430 nm)

Quantum yield: 26%

Lifetime: < 1 ns

Quantum yield: 35%

Lifetime: 7.6 ns

Crystal structure determinations

Crystals suitable for single-crystal X-ray diffraction were selected, coated in perfluoropolyether oil, mounted on a polyimide microloop (MicroMounts™ from MiTeGen), and transferred to a stream of cold nitrogen (Bruker Kryoflex2 or Oxford Cryostream 700 or 800). Diffraction data were collected on Bruker Apex II 4-circle diffractometers with CCD area detectors using Mo-K_α radiation monochromated by graphite (for **3**, **5I**, **6**, and **8**) or multi-layer focusing mirrors (for **4** and **11**) at 100 K. Diffraction data of **6** were collected on a Rigaku Oxford Diffraction XtaLAB Synergy Dualflex diffractometer with a Pilatus 300 K area detector and multi-layer mirror monochromated Mo-K_α radiation at 220 K. The images were processed and corrected for Lorentz-polarization effects and absorption as implemented in the Bruker software packages (**3-5I**, **6, 8**, and **11**) or using the CrysAlisPro software from Rigaku Oxford Diffraction (**9**). The structures were solved using the intrinsic phasing method (SHELXT)[3] and Fourier expansion technique. All non-hydrogen atoms were refined in anisotropic approximation, with hydrogen atoms "riding" in idealized positions, by full-matrix least squares against F^2 of all data, using SHELXL[4] software and the SHELXLE graphical user interface.[5] The crystal of compound **3** was a non-merohedral twin with domains rotated by 179.9° around the real axis [0.097 -0.001 1.000]. The twin fraction was refined to 31.5%. Crystals of compound **9** showed reflection splitting and decreased diffraction quality at 200 K, which worsened on further lowering the temperature. This can be attributed to the occurrence of a low-temperature phase transition. As the crystals were already optically identified as twins between the crossed polarizers at ambient temperature, the crystal structure was solved at 220 K, considering a non-merohedral twin with domains rotated by 179.97° around reciprocal axis (0 0 1) or real axis [-0.08 -0.43 0.90]. The twin fraction was refined to 40.3%. Diamond[6] software was used for graphical representation. Crystal data and experimental details are listed in Table S1; full structural information has been deposited with the Cambridge Crystallographic Data Centre. CCDC-1940941 (**3**), 1940942 (**4**), 1940945 (**5I**), 1940946 (**6**), 1940943 (**8**), 1940944 (**9**), and 1940947 (**11**).

Table S1. Single-crystal X-ray diffraction data and structure refinements of **3-5I**, **6**, **8**, **9** and **11**.

Data	**3**	**4**	**5I**	**6**
CCDC number	1940941	1940942	1940945	1940946
Empirical formula	$C_{25}H_{20}$	$C_{39}H_{30}$	$C_{32}H_{28}$	$C_{31}H_{26}$
Formula weight / $g{\cdot}mol^{-1}$	320.41	498.63	412.54	398.52
T / K	100(2)	100(2)	100(2)	100(2)
λ / Å, radiation	0.71073, MoK_{α}	0.71073, MoK_{α}	0.71073, MoK_{α}	0.71073, MoK_{α}
Crystal size / mm^3	0.09×0.30×0.77	0.38×0.42×0.51	0.11×0.30×0.53	0.17×0.31×0.47
Crystal color, habit	colorless plate	colorless block	colorless plate	colorless plate
μ / mm^{-1}	0.067	0.069	0.067	0.069
Crystal system	monoclinic	monoclinic	monoclinic	monoclinic
Space group	$P2/c$	$P2_1/c$	$P2_1/c$	$P2_1/c$
a / Å	5.8917(16)	9.271(5)	10.250(6)	9.940(5)
b / Å	8.1086(18)	10.323(7)	26.097(14)	25.777(10)
c / Å	18.714(4)	28.395(15)	8.582(5)	8.527(4)
α / °	90	90	90	90
β / °	91.768(8)	92.585(16)	94.73(2)	96.776(15)
γ / °	90	90	90	90
Volume / $Å^3$	893.6(4)	2715(3)	2288(2)	2169.6(17)
Z	2	4	4	4
ρ_{calc} / $g{\cdot}cm^{-3}$	1.191	1.220	1.198	1.220
F(000)	340	1056	880	848
θ range / °	2.512-28.276	2.099-26.017	2.506-26.016	2.209-30.134
Reflections collected	18896	24862	12924	56981
Unique reflections	3402	5352	4483	6361
Parameters / restraints	116 / 0	382 / 20	292 / 0	282 / 0
GooF on F^2	1.070	1.022	1.041	1.026
R_1 [I>2σ(I)]	0.0446	0.0364	0.0581	0.0469
wR^2 (all data)	0.1098	0.0930	0.1300	0.1258
Max. / min. residual electron density / $e{\cdot}Å^{-3}$	0.252 / -0.219	0.255 / –0.205	0.364 / –0.256	0.376 / –0.239

Table S1. Continued

Data	**8**	**9**	**11**
CCDC number	1940943	1940944	1940947
Empirical formula	$C_{50}H_{40}\cdot CHCl_3$	$C_{36}H_{28}$	$C_{50}H_{38}$
Formula weight / $g\cdot mol^{-1}$	760.19	460.58	638.80
T / K	100(2)	220(2)	100(2)
λ / Å, radiation	0.71073, MoK_{α}	0.71073, MoK_{α}	0.71073, MoK_{α}
Crystal size / mm^3	0.12×0.30×0.65	0.07×0.15×0.32	0.09×0.27×0.63
Crystal color, habit	colorless plate	colorless plate	brown plate
μ / mm^{-1}	0.267	0.066	0.072
Crystal system	monoclinic	triclinic	triclinic
Space group	$P2_1/c$	$P\bar{1}$	$P\bar{1}$
a / Å	44.944(11)	7.4118(2)	10.811(3)
b / Å	7.3084(16)	21.8784(7)	13.318(4)
c / Å	52.154(11)	34.0649(11)	13.475(5)
α / °	90	72.165(3)	69.25(3)
β / °	112.211(9)	88.447(2)	68.230(17)
γ / °	90	88.413(2)	76.65(3)
Volume / $Å^3$	15860(6)	5255.5(3)	1673.8(10)
Z	16	8	2
ρ_{calc} / $g\cdot cm^{-3}$	1.273	1.164	1.268
F(000)	6368	1952	676
θ range / °	1.514-26.022	1.957-25.350	1.646-26.077
Reflections collected	144318	102337	30235
Unique reflections	31163	25578	6545
Parameters / restraints	1961 / 0	1310 / 0	455 / 0
GooF on F^2	1.003	1.035	1.015
R_1 [$I>2\sigma(I)$]	0.0644	0.0521	0.0489
wR^2 (all data)	0.1657	0.1477	0.1258
Max. / min. residual electron density / $e\cdot Å^{-3}$	0.343 / –0.429	0.188 / –0.164	0.251 / –0.236

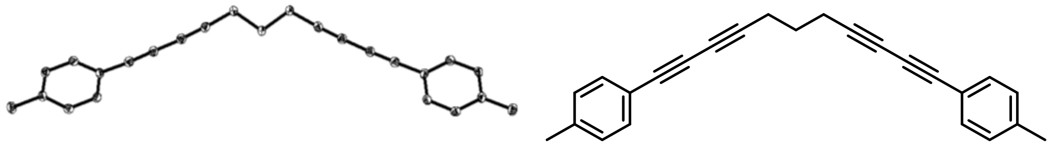

Figure S10. Molecular structure of **3** in the solid state at 100 K. Atomic displacement ellipsoids are drawn at the 50% probability level, and H atoms are omitted for clarity.

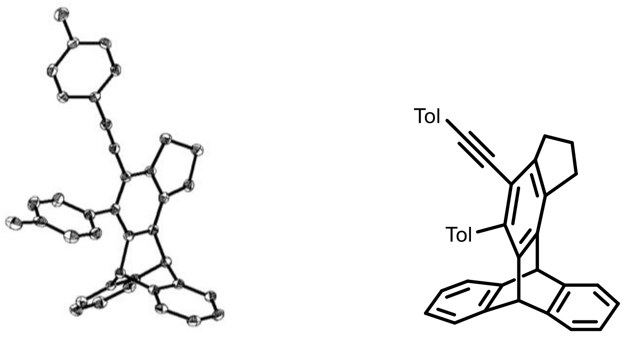

Figure S11. Molecular structure of **4** in the solid state at 100 K. Atomic displacement ellipsoids are drawn at the 50% probability level, and H atoms are omitted for clarity. Only the major part (88%) of the disordered $(CH_2)_3$ moiety is shown here.

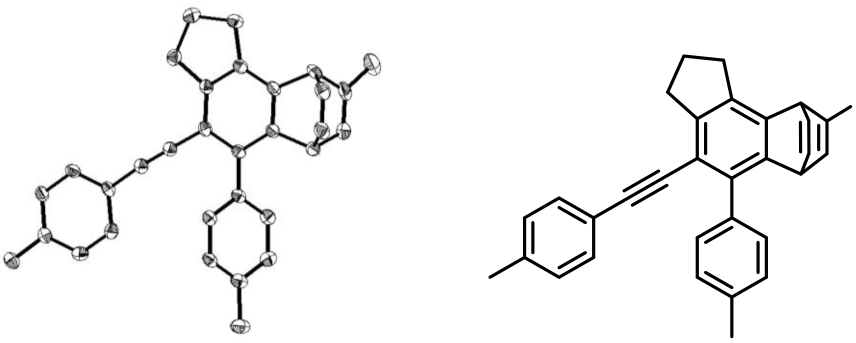

Figure S12. Molecular structure of **5l** in the solid state at 100 K. Atomic displacement ellipsoids are drawn at the 50% probability level, and H atoms are omitted for clarity.

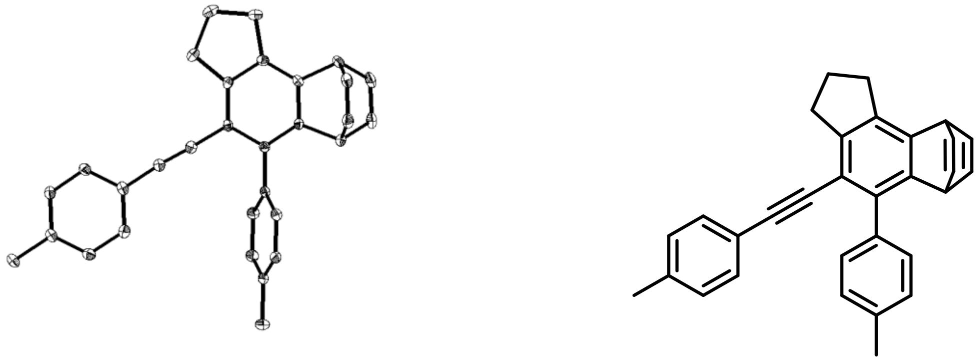

Figure S13. Molecular structure of **6** in the solid state at 100 K. Atomic displacement ellipsoids are drawn at the 50% probability level, and H atoms are omitted for clarity.

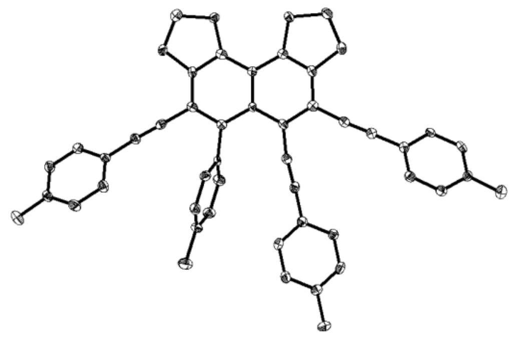

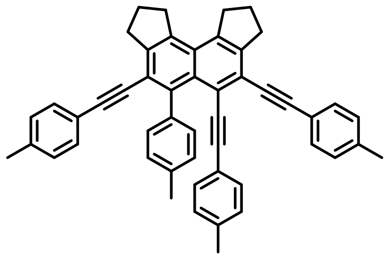

Figure S14. Molecular structure of **8** in the solid state at 100 K. Atomic displacement ellipsoids are drawn at the 50% probability level. Hydrogen atoms and co-crystallized $CHCl_3$ solvent molecules are omitted for clarity. Only one of the four symmetry-independent molecules is shown here.

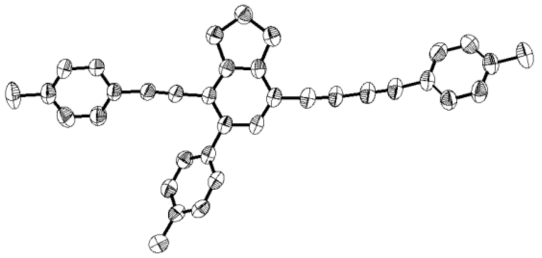

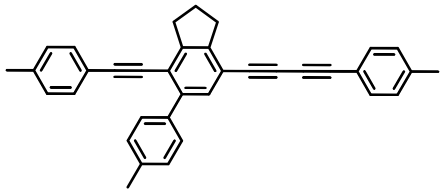

Figure S15. Molecular structure of **9** in the solid state at 220 K. Atomic displacement ellipsoids are drawn at the 50% probability level, and H atoms are omitted for clarity. Only one of the four symmetry-independent molecules is shown here.

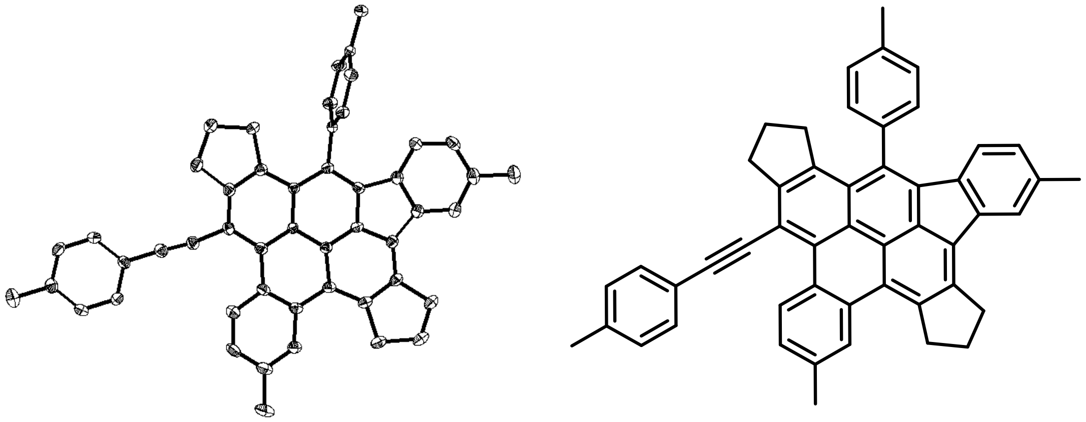

Figure S16. Molecular structure of **11** in the solid state at 100 K. Atomic displacement ellipsoids are drawn at the 50% probability level, and H atoms are omitted for clarity.

Computational details 1

The geometries of **4**, **5**, **6**, **8**, **9** and **11** were optimized without symmetry constraints using the Gaussian 09 and Gaussian 03 program packages.[7] The optimized structures were verified as minima on the potential energy surface by calculation of the vibrational frequencies. The DFT computations were performed using the B3LYP functional and the 6-31+g(d) basis sets.[8] Excited state properties were obtained from time-dependent DFT calculations at the CAM-B3LYP/6-31G(d,p) level.[9] The simulated gas-phase and CH_2Cl_2 solution (PCM model) absorption spectra are shown in the following Figures using Gaussian functions with halfband-widths of 1000 cm^{-1}.

HOMO-1, HOMO, LUMO and LUMO+1 of 4

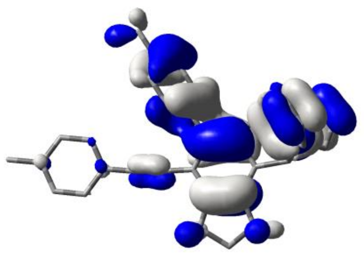

HOMO-1 (131)

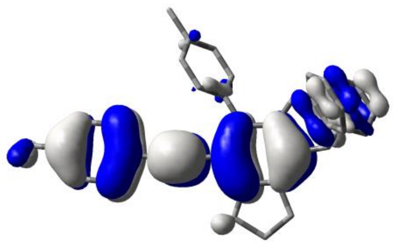

HOMO (132)

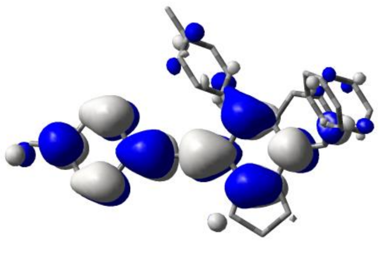

LUMO (133)

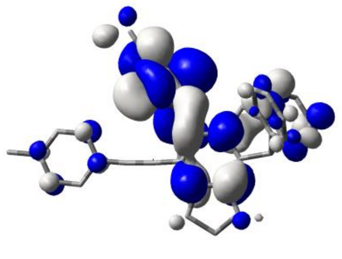

LUMO+1 (134)

Simulated UV/Vis spectra of 4 in the gas phase (left) and in CH_2Cl_2 (right)

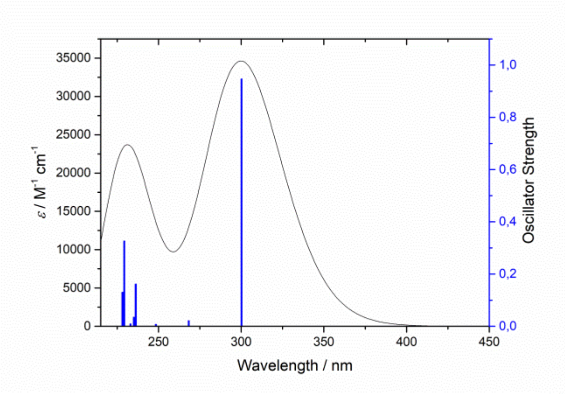

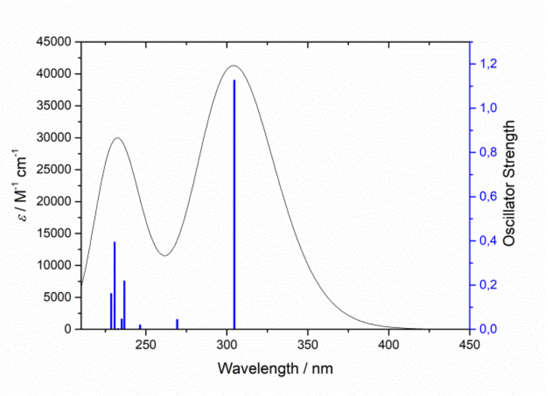

Lowest energy singlet electronic transitions of 4 in the gas phase

State	*E (eV)*	λ (nm)	*f*	Major contributions [>10%]
1	4.13	300	0.948	132→133 (91%)
2	4.62	268	0.022	131→133 (51%), 132→134 (20%)
3	4.99	248	0.008	122→133 (47%), 128→133 (15%), 129→133 (19%)
4	5.05	246	0.002	124→133 (28%), 132→138 (38%)
5	5.25	236	0.162	131→133 (25%), 132→134 (45%)
6	5.28	235	0.036	130→136 (19%), 131→136 (18%), 132→136 (15%)
7	5.32	233	0.010	131→137 (11%)
8	5.41	229	0.327	130→133 (33%), 131→134 (19%)

Lowest energy singlet electronic transitions of 4 in CH_2Cl_2

State	*E (eV)*	λ (nm)	*f*	Major contributions [>10%]
1	4.07	305	1.128	132→133 (92%)
2	4.60	269	0.045	131→133 (56%), 132→134 (16%)
3	5.03	246	0.020	122→133 (49%), 127→133 (16%), 129→133 (15%)
4	5.05	245	0.003	124→138 (29%), 132→138 (42%)
5	5.24	237	0.219	131→133 (20%), 132→134 (45%)

6	5.27	235	0.048	130→136 (21%), 131→136 (17%), 132→136 (16%)
7	5.33	233	0.004	131→139 (16%)
8	5.38	231	0.395	130→133 (35%), 131→134 (20%)

HOMO-1, HOMO, LUMO and LUMO+1 of 5I

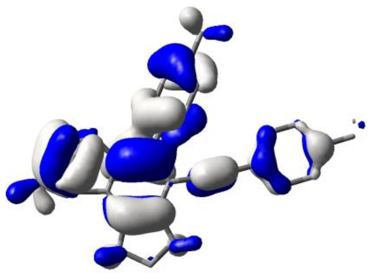

HOMO-1 (109)

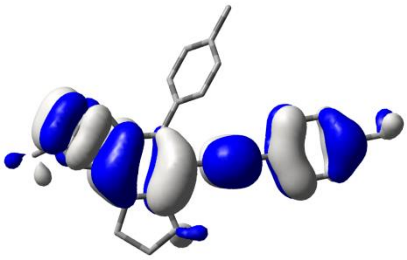

HOMO (110)

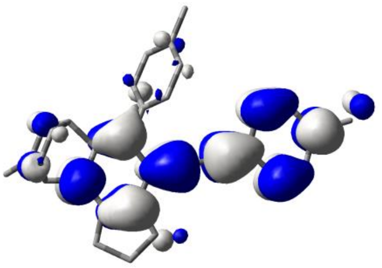

LUMO (111)

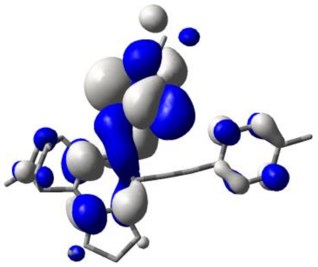

LUMO+1 (112)

Simulated UV/Vis spectra of 5I in the gas phase (left) and in CH_2Cl_2 (right)

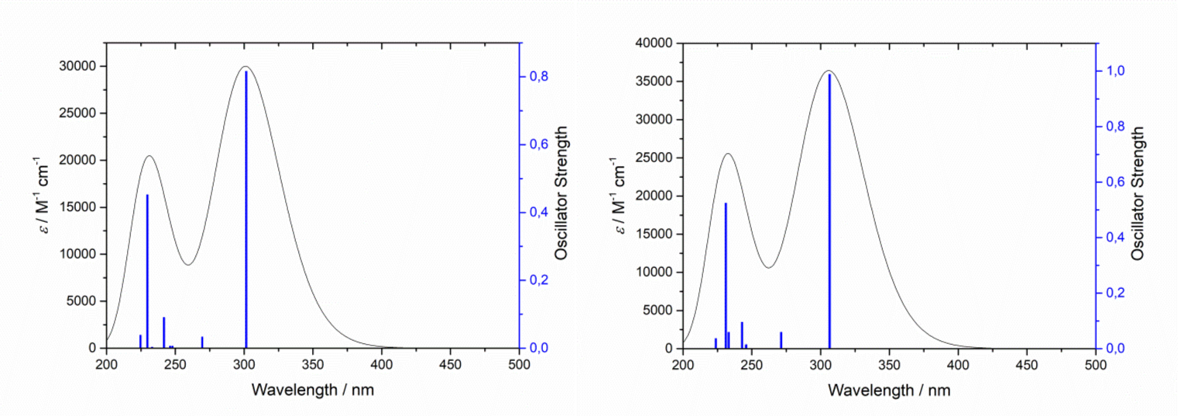

Lowest energy singlet electronic transitions of 5I in the gas phase

State	*E (eV)*	λ (nm)	*f*	Major contributions [>10%]
1	4.11	302	0.816	110→111 (92%)
2	4.60	270	0.033	109→111 (59%), 110→112 (16%)
3	5.00	248	0.006	102→111 (35%), 107→111 (36%)
4	5.04	246	0.006	105→111 (26%), 110→114 (41%)
5	5.13	242	0.091	108→111 (12%), 109→111 (18%), 110→112 (33%), 110→115 (12%)
6	5.32	233	0.003	106→112 (15%), 109→113 (22%)
7	5.40	230	0.453	108→111 (22%), 109→112 (29%), 110→112 (21%), 110→115 (10%)
8	5.52	225	0.038	108→116 (15%), 109→116 (18%), 110→116 (48%)

Lowest energy singlet electronic transitions of 5I in CH_2Cl_2

State	*E (eV)*	λ (nm)	*f*	Major contributions [>10%]
1	4.04	306	0.989	110→111 (91%)
2	4.57	271	0.059	109→111 (64%), 110→112 (12%)
3	5.04	246	0.015	105→111 (26%), 110→113 (28%)
4	5.05	246	0.011	102→111 (39%), 107→111 (29%)
5	5.10	243	0.095	108→111 (18%), 109→111 (13%), 110→112 (25%), 110→115 (12%)
6	5.32	233	0.059	106→112 (15%), 109→114 (14%)
7	5.36	231	0.525	108→111 (20%), 109→112 (25%), 110→112 (21%)
8	5.54	224	0.037	108→116 (18%), 109→116 (20%), 110→116 (50%)

HOMO-1, HOMO, LUMO and LUMO+1 of 5II

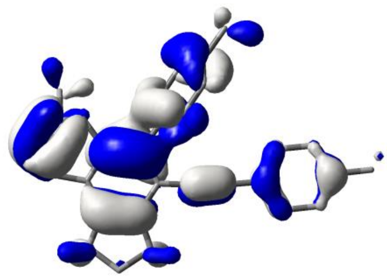

HOMO-1 (109)

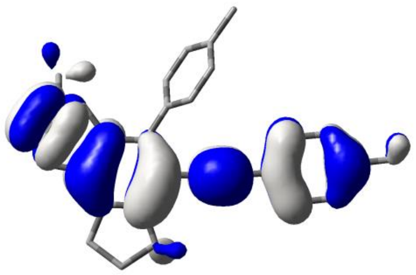

HOMO (110)

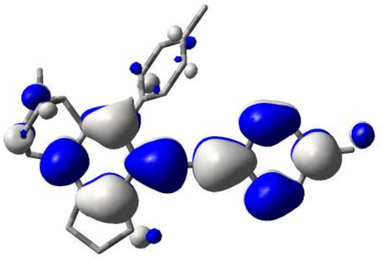

LUMO (111)

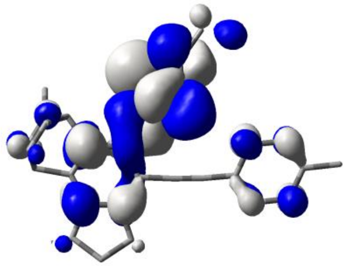

LUMO+1 (112)

Simulated UV/Vis spectra of 5II in the gas phase (left) and CH_2Cl_2 (right)

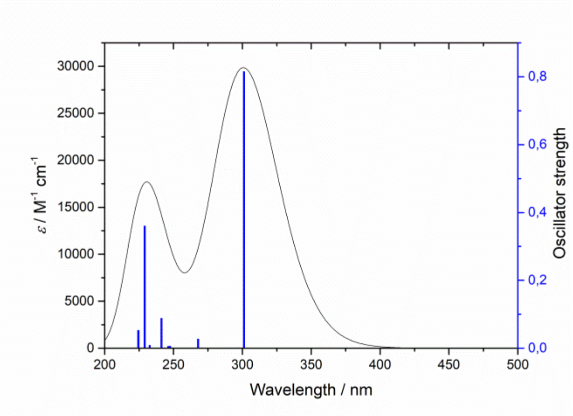

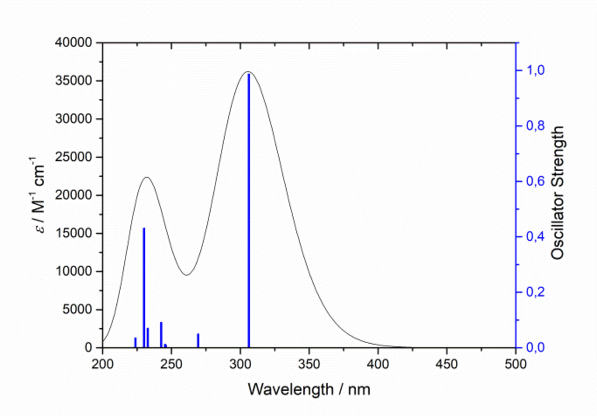

Lowest energy singlet electronic transitions of 5II in the gas phase

State	*E (eV)*	λ (nm)	*f*	Major contributions [>10%]
1	4.12	301	0.815	110→111 (92%)
2	4.63	268	0.027	109→111 (56%), 110→112 (17%)
3	5.01	248	0.006	102→111 (35%), 107→111 (32%)
4	5.04	246	0.006	105→111 (25%), 110→114 (42%)
5	5.14	241	0.088	108→111 (13%), 109→111 (20%), 110→112 (29%), 110→115 (13%)
6	5.33	233	0.009	106→112 (19%), 107→113 (13%), 109→113 (22%), 110→113 (11%)
7	5.41	229	0.360	108→111 (20%), 109→112 (22%), 110→112 (24%), 110→115 (11%)
8	5.52	224	0.053	108→116 (14%), 109→116 (15%), 110→116 (47%)

Lowest energy singlet electronic transitions of 5II in CH_2Cl_2

State	*E (eV)*	λ (nm)	*f*	Major contributions [>10%]
1	4.05	306	0.988	110→111 (91%)
2	4.60	269	0.050	109→111 (61%), 110→112 (13%)
3	5.04	246	0.010	105→111 (27%), 110→113 (11%), 110→114 (28%)
4	5.05	245	0.013	102→111 (41%), 107→111 (25%)
5	5.11	242	0.092	108→111 (19%), 109→111 (14%), 110→112 (21%), 110→115 (12%)
6	5.33	233	0.071	106→112 (17%), 109→113 (13%)
7	5.39	230	0.432	108→111 (20%), 109→112 (18%), 110→112 (25%)
8	5.54	224	0.036	108→116 (18%), 109→116 (17%), 110→116 (48%)

HOMO-1, HOMO, LUMO and LUMO+1 of 5III

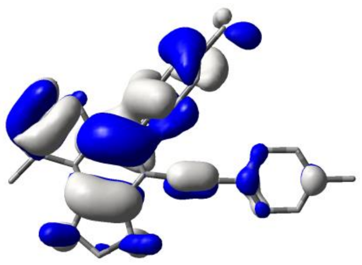

HOMO-1 (109)

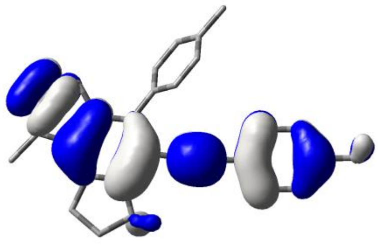

HOMO (110)

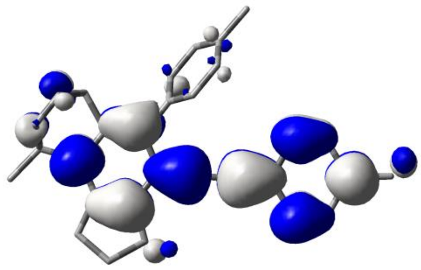

LUMO (111)

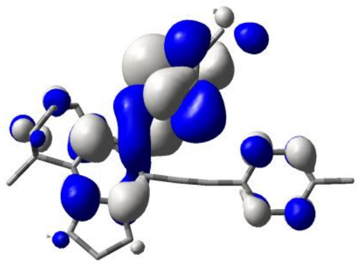

LUMO+1 (112)

Simulated UV/Vis spectra of 5III in the gas phase (left) and CH_2Cl_2 (right)

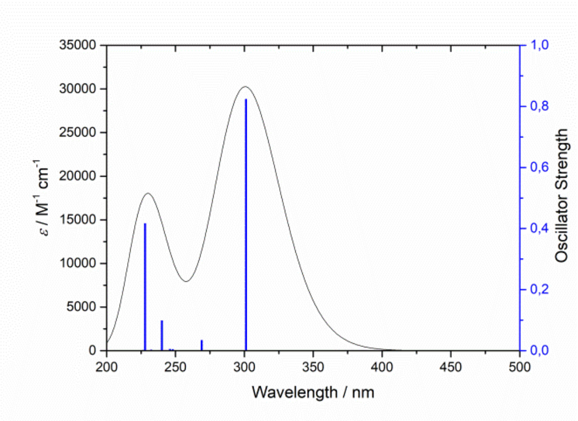

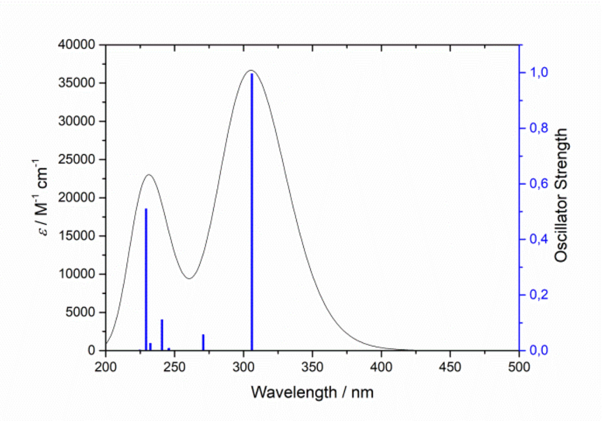

Lowest energy singlet electronic transitions of 5III in the gas phase

State	*E (eV)*	λ (nm)	*f*	Major contributions [>10%]
1	4.12	301	0.824	110→111 (92%)
2	4.61	269	0.034	109→111 (61%), 110→112 (15%)
3	5.00	248	0.004	102→111 (25%), 104→111 (20%), 107→111 (33%)
4	5.04	246	0.005	105→111 (26%), 110→114 (42%)
5	5.16	240	0.098	108→111 (12%), 109→111 (16%), 110→112 (35%), 110→116 (12%)
6	5.34	232	0.003	106→112 (21%), 107→113 (12%), 109→113 (22%)
7	5.44	228	0.416	108→111 (22%), 109→112 (27%), 110→112 (22%)
8	5.48	226	0.002	108→115 (17%), 109→115 (16%), 110→115 (53%)

Lowest energy singlet electronic transitions of 5III in CH_2Cl_2

State	*E (eV)*	λ (nm)	*f*	Major contributions [>10%]
1	4.05	306	0.997	110→111 (92%)
2	4.58	271	0.058	109→111 (65%), 110→112 (11%)
3	5.04	246	0.009	102→111 (38%), 104→111 (14%), 107→111 (27%)
4	5.05	246	0.007	105→111 (28%), 110→113 (13%), 110→114 (27%)
5	5.15	241	0.112	108→111 (17%), 109→111 (12%), 110→112 (29%), 110→115 (12%)
6	5.33	232	0.026	106→112 (20%), 109→113 (14%)
7	5.41	229	0.511	108→111 (21%), 109→112 (26%), 110→112 (23%)
8	5.52	225	0.003	108→116 (20%), 109→116 (17%), 110→116 (55%)

HOMO-1, HOMO, LUMO and LUMO+1 of 5IV

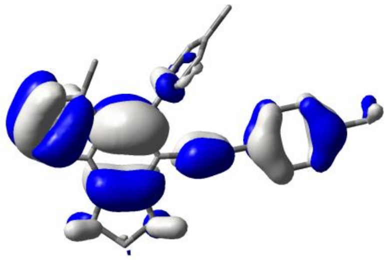

HOMO-1 (109)

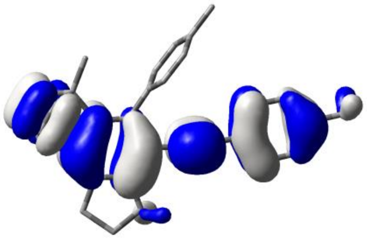

HOMO (110)

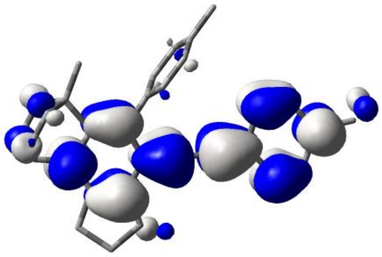

LUMO (111)

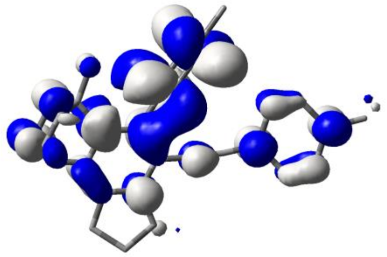

LUMO+1 (112)

Simulated UV/Vis spectra of 5IV in the gas phase (left) and CH_2Cl_2 (right)

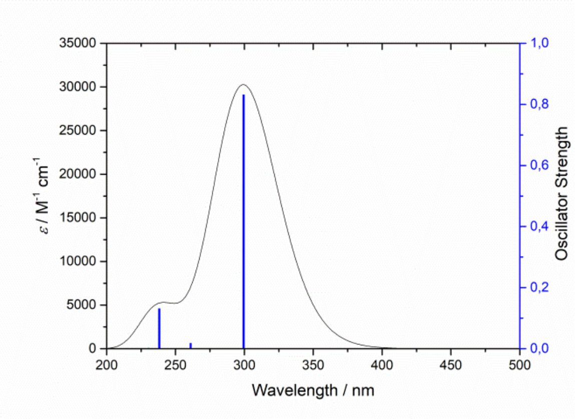

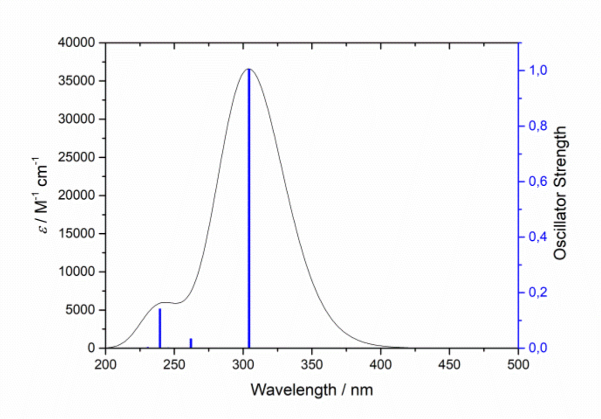

Lowest energy singlet electronic transitions of 5IV in the gas phase

State	*E (eV)*	λ (nm)	*f*	Major contributions [>10%]
1	4.14	299	0.832	110→111 (92%)
2	4.75	261	0.018	109→111 (47%), 110→117 (13%)
3	5.00	248	0.007	102→111 (19%), 104→111 (45%), 108→111 (27%)
4	5.04	246	0.001	105→111 (21%), 110→114 (43%)
5	5.20	238	0.131	107→111 (13%), 109→111 (26%), 110→112 (28%), 110→116 (16%)
6	5.38	230	0.002	106→113 (26%), 108→112 (37%), 108→116 (15%)
7	5.48	226	0.0001	107→115 (12%), 109→115 (20%), 110→115 (62%)
8	5.57	223	0.0002	110→113 (80%), 110→119 (10%)

Lowest energy singlet electronic transitions of 5IV in CH_2Cl_2

State	*E (eV)*	λ (nm)	*f*	Major contributions [>10%]
1	4.07	304	1.009	110→111 (92%)
2	4.73	262	0.035	109→111 (52%), 110→117 (13%)
3	5.03	246	0.001	102→111 (37%), 104→111 (24%), 108→111 (30%)
4	5.05	246	0.002	105→111 (27%), 110→113 (42%)
5	5.18	240	0.145	107→111 (17%), 109→111 (22%), 110→112 (31%), 110→115 (14%)
6	5.37	231	0.004	106→114 (30%), 108→112 (33%), 108→115 (18%)
7	5.52	225	0.0001	107→116 (13%), 109→116 (20%), 110→116 (60%)
8	5.65	220	0.002	108→111 (48%)

HOMO-1, HOMO, LUMO and LUMO+1 of 6

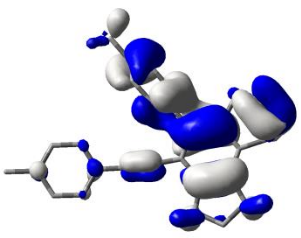

HOMO-1 (105)

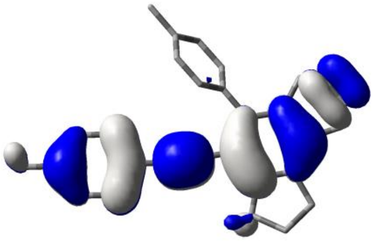

HOMO (106)

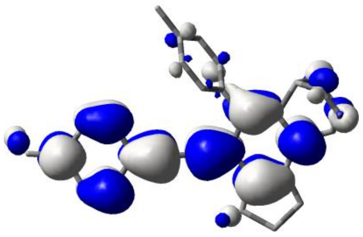

LUMO (107)

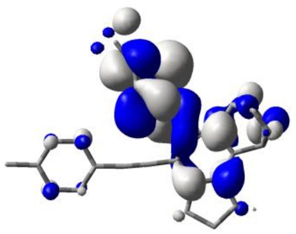

LUMO+1 (108)

Simulated UV/Vis spectra of 6 in the gas phase and CH_2Cl_2

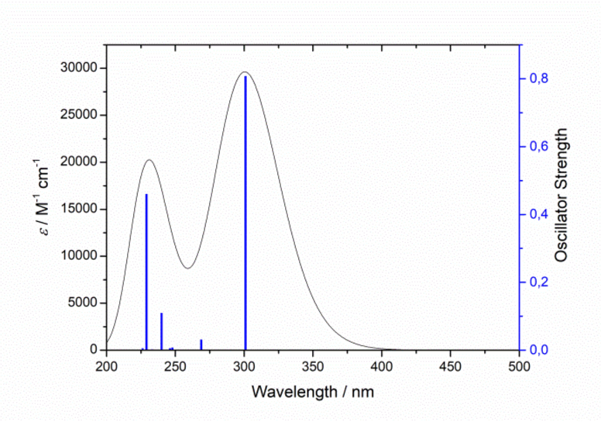

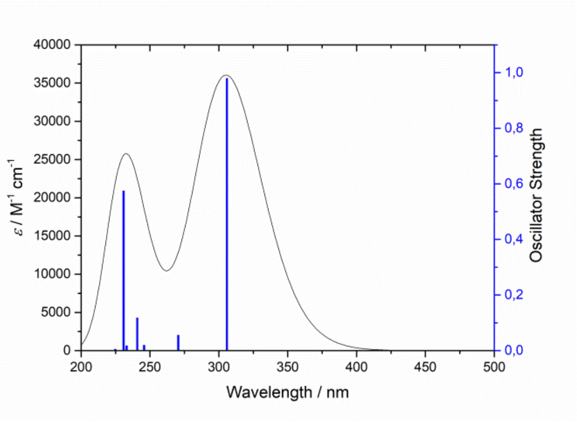

Lowest energy singlet electronic transitions of 6 in the gas phase

State	*E (eV)*	λ (nm)	*f*	Major contributions [>10%]
1	4.12	301	0.807	106→107 (92%)
2	4.61	269	0.030	105→107 (59%), 106→108 (17%)
3	5.00	248	0.007	98→107 (22%), 99→107 (10%), 100→107 (16%), 103→107 (36%)
4	5.04	246	0.005	101→107 (26%), 106→110 (43%)
5	5.17	240	0.109	105→107 (18%), 106→108 (38%), 106→112 (11%)
6	5.32	233	0.002	102→108 (16%), 105→109 (23%), 106→109 (10%)
7	5.41	229	0.460	104→107 (23%), 105→108 (31%), 106→108 (16%), 106→112 (13%)
8	5.48	226	0.048	104→111 (20%), 105→111 (18%), 106→111 (57%)

Lowest energy singlet electronic transitions of 6 in CH_2Cl_2

State	*E (eV)*	λ (nm)	*f*	Major contributions [>10%]
1	4.05	306	0.980	106→107 (92%)
2	4.58	270	0.056	105→107 (64%), 106→108 (13%)
3	5.05	246	0.012	98→107 (32%), 100→107 (12%), 103→107 (28%)
4	5.05	246	0.004	101→107 (25%), 106→109 (21%), 106→110 (18%)
5	5.15	241	0.118	104→107 (14%), 105→107 (13%), 106→108 (34%), 106→111 (11%)
6	5.32	233	0.017	102→108 (17%), 105→110 (13%)
7	5.37	231	0.575	104→107 (23%), 105→108 (31%), 106→108 (17%)
8	5.52	225	0.005	104→112 (22%), 105→112 (18%), 106→112 (54%)

HOMO-1, HOMO, LUMO and LUMO+1 8

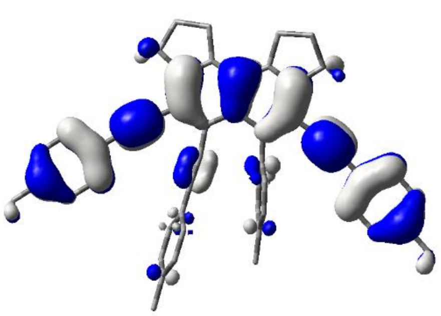

HOMO-1 (169)

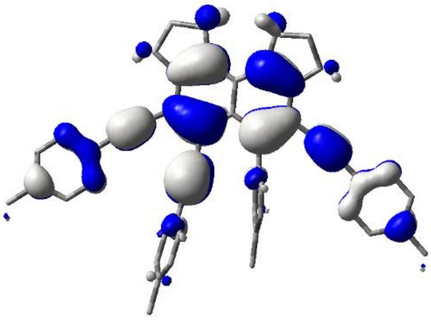

HOMO (170)

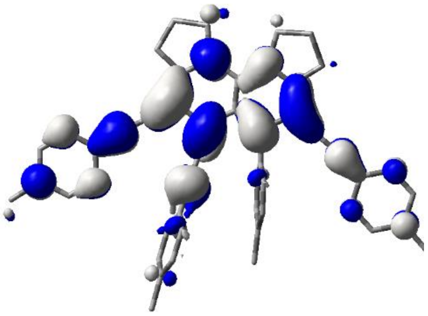

LUMO (171)

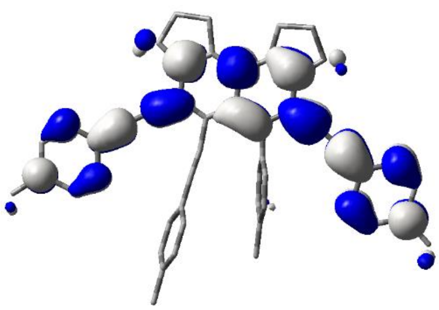

LUMO+1 (172)

Simulated UV/Vis spectra of 8 in the gas phase (left) and in CH_2Cl_2 (right)

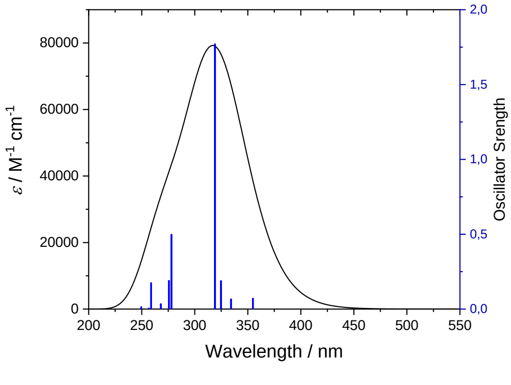

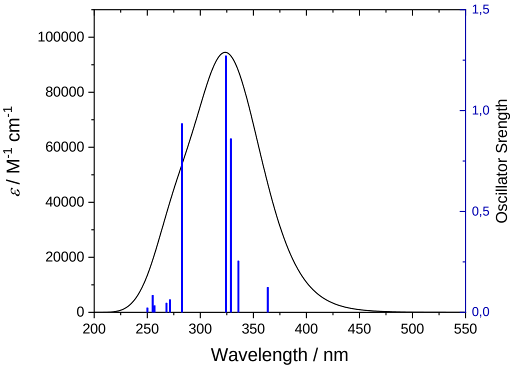

Lowest energy singlet electronic transitions of 8 in the gas phase

State	*E (eV)*	λ (nm)	*f*	Major contributions [>10%]
1	3.49	355	0.072	170→171 (87%)
2	3.71	334	0.068	169→171 (52%), 170→172 (26%)
3	3.82	325	0.190	168→171 (68%), 170→172 (10%),
4	3.89	319	1.772	168→171 (18%), 169→171 (22%), 170→172 (48%)
5	4.46	278	0.499	169→172 (38%), 170→173 (34%)
6	4.50	276	0.191	167→171 (10%), 169→172 (23%), 170→173 (39%)
7	4.63	268	0.035	165→171 (16%), 168→172 (49%)
8	4.79	259	0.177	168→173 (71%)

Lowest energy singlet electronic transitions of 8 in CH_2Cl_2

State	*E (eV)*	λ (nm)	*f*	Major contributions [>10%]
1	3.41	363	0.124	170→171 (88%)
2	3.69	336	0.255	169→171 (62%), 170→172 (15%)
3	3.77	329	0.861	168→171 (31%), 169→171 (16%), 170→172 (30%)
4	3.82	324	1.271	168→171 (55%), 170→172 (29%)
5	4.39	283	0.935	167→171 (21%), 169→172 (59%)
6	4.59	271	0.063	170→173 (61%)
7	4.63	268	0.046	165→171 (12%), 168→172 (49%)
8	4.83	257	0.033	164→171 (15%), 166→171 (21%), 168→173 (22%)

HOMO-1, HOMO, LUMO and LUMO+1 9

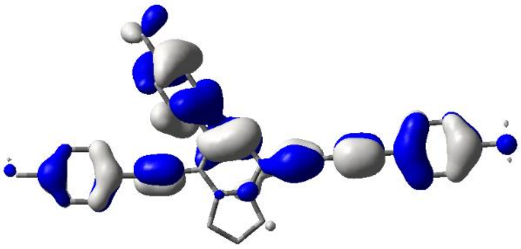

HOMO-1 (121)

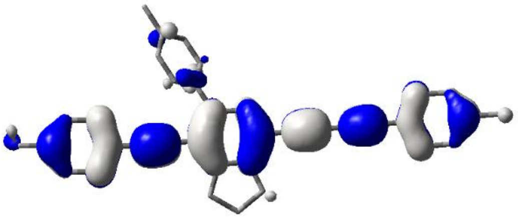

HOMO (122)

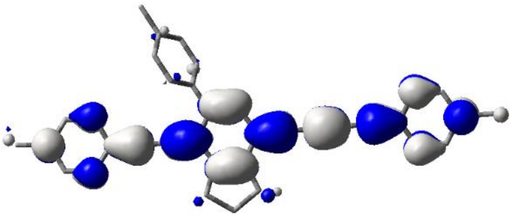

LUMO (123)

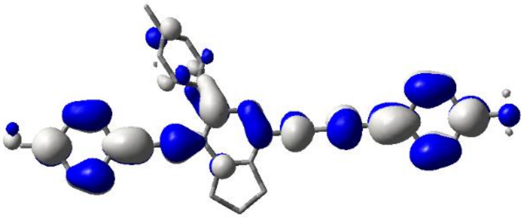

LUMO+1 (124)

Simulated UV/Vis spectra of 9 in the gas phase (left) and CH_2Cl_2 (right)

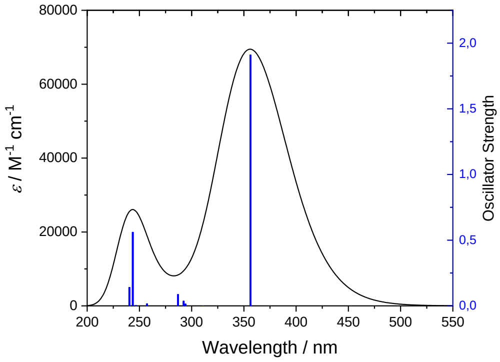

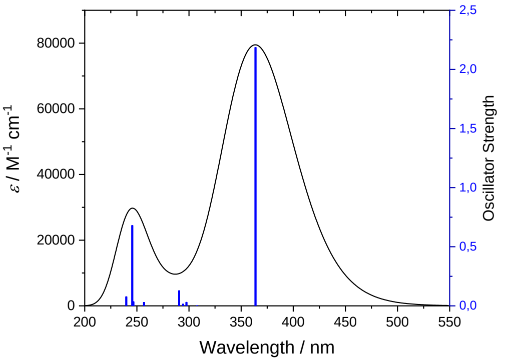

Lowest energy singlet electronic transitions of 9 in the gas phase

State	*E (eV)*	λ (nm)	*f*	Major contributions [>10%]
1	3.48	356	1.910	122→123 (85%)
2	3.99	311	0.001	119→123 (70%), 119→124 (11%)
3	4.22	294	0.014	121→123 (21%), 121→126 (11%), 122→124 (11%), 122→126 (35%)
4	4.24	292	0.037	121→123 (35%), 122→126 (22%)
5	4.32	287	0.087	120→123 (58%), 122→125 (11%)
6	4.82	257	0.017	113→123 (42%), 113→124 (13%), 118→123 (21%)
7	5.02	247	0.003	116→123 (19%), 116→124 (14%), 121→128 (11%), 122→128 (26%)
8	5.09	244	0.561	120→123 (22%), 122→124 (14%), 122→125 (35%)

Lowest energy singlet electronic transitions of 9 in CH_2Cl_2

State	*E (eV)*	λ (nm)	*f*	Major contributions [>10%]
1	3.41	364	2.187	122→123 (86%)
2	4.03	308	0.002	119→123 (60%)
3	4.17	298	0.031	121→123 (42%), 122→124 (22%)
4	4.21	294	0.016	119→123 (16%), 121→123 (11%), 121→126 (13%), 122→126 (38%)
5	4.27	291	0.128	120→123 (61%)
6	4.83	257	0.031	113→123 (40%), 113→124 (13%), 118→123 (11%)
7	5.03	247	0.036	117→123 (21%), 117→124 (13%), 122→128 (22%)
8	5.05	246	0.681	120→123 (15%), 122→125 (38%)

HOMO-1, HOMO, LUMO and LUMO+1 of 11

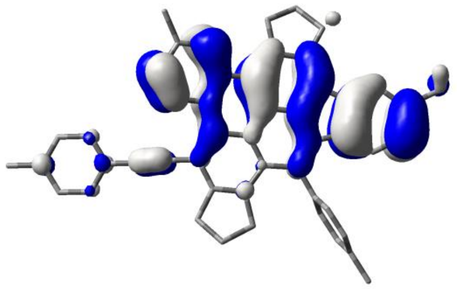

HOMO-1 (168)

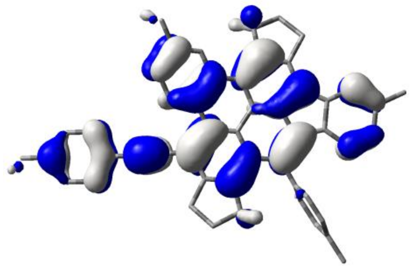

HOMO (169)

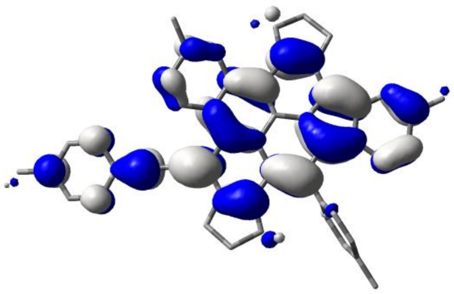

LUMO (170)

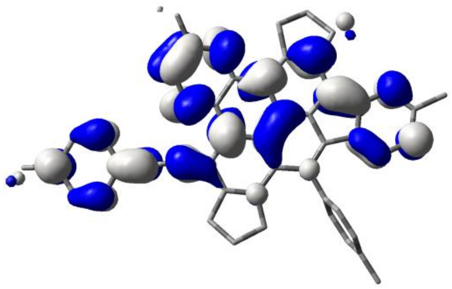

LUMO+1 (171)

Simulated UV/Vis spectra of 11 in the gas phase (left) and CH_2Cl_2 (right)

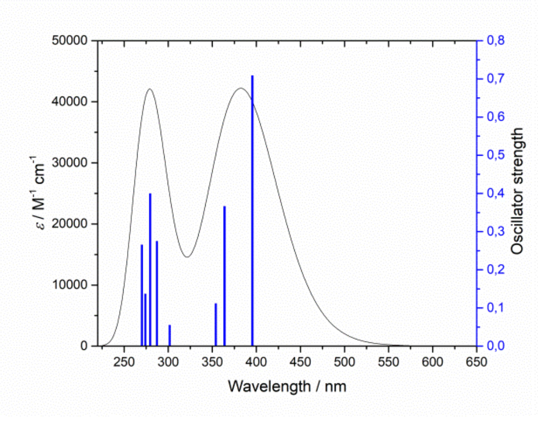

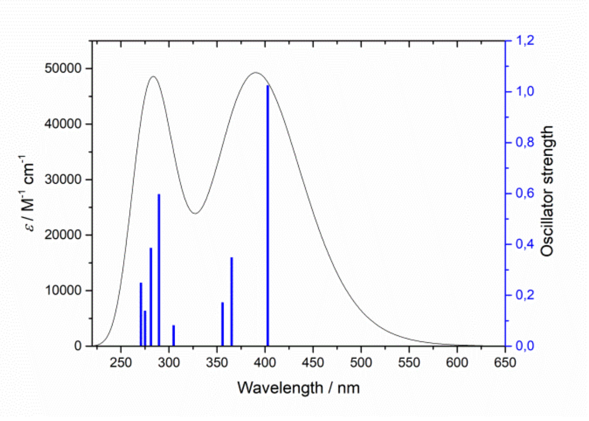

Lowest energy singlet electronic transitions of 11 in the gas phase

State	*E (eV)*	λ (nm)	*f*	Major contributions [>10%]
1	3.14	395	0.709	169→170 (85%)
2	3.41	364	0.366	167→170 (67%)
3	3.50	354	0.112	168→170 (76%)
4	4.11	302	0.055	166→170 (41%), 169→171 (35%)
5	4.32	287	0.275	166→170 (20%), 169→171 (38%)
6	4.43	280	0.400	165→170 (33%), 167→171 (26%), 169→172 (12%)
7	4.52	274	0.137	165→170 (10%), 168→172 (11%), 169→172 (21%), 169→173 (15%)
8	4.59	270	0.265	163→170 (15%), 169→172 (33%), 169→173 (12%)

Lowest energy singlet electronic transitions of 11 in CH_2Cl_2

State	*E (eV)*	λ (nm)	*f*	Major contributions [>10%]
1	3.08	403	1.024	169→170 (91%)
2	3.39	365	0.348	167→170 (77%)
3	3.49	356	0.171	168→170 (83%)
4	4.07	305	0.082	166→170 (41%), 169→171 (39%)
5	4.28	290	0.597	166→170 (22%), 167→171 (15%), 169→171 (33%)
6	4.41	281	0.386	164→170 (30%), 167→171 (15%), 169→172 (19%)
7	4.51	275	0.138	164→170 (18%), 169→172 (22%), 169→173 (12%)
8	4.58	271	0.249	163→170 (14%), 169→172 (24%), 169→173 (14%)

NICS B3LYP / 6-311+g(d) values for selected rings of compound 11

Ring	NICS(0)	NICS(1)
**Benzene**	-7.88	-10.13
**A**	-5.36	-7.55
**B**	5.29	0.64
**C**	-7.86	-10.34
**D**	-3.24	-6.28
**E**	0.45	-3.86
**F**	-7.14	-10.03
**G**	-8.05	-9.31
**H**	-7.17	-9.16
**I**	-7.11	-8.91

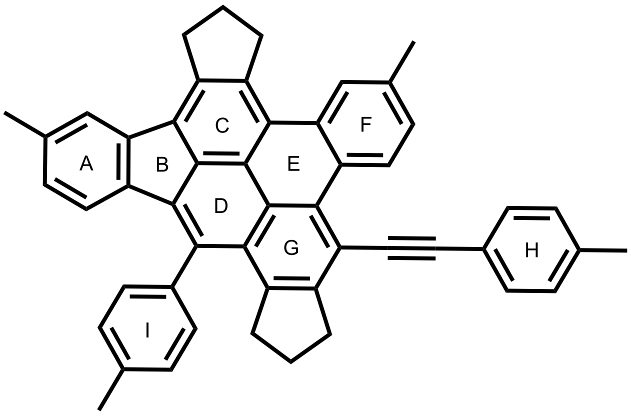

Optimized geometry of 4 (gas phase)

6	0.088516	-2.484718	-0.040495	6	3.541348	1.689930	2.388947
6	-0.608438	-1.269315	0.006627	1	3.009306	2.637219	2.435709
6	0.126482	-0.043322	0.030546	6	4.404772	1.307116	3.426674
6	1.518633	-0.103778	0.021715	1	4.544005	1.962986	4.281871
6	1.490564	-2.520184	-0.061051	6	5.083545	0.088979	3.365216
6	2.205799	-1.333670	-0.026677	1	5.751082	-0.201617	4.171991
6	1.983693	-3.951058	-0.095816	6	4.907630	-0.764529	2.265346
1	2.321869	-4.260789	0.904441	1	5.435207	-1.714451	2.216090
1	2.828274	-4.101850	-0.777719	6	-0.594027	1.257588	0.036060
6	0.723256	-4.743957	-0.527608	6	-0.462242	2.150614	-1.037305
1	0.701307	-5.759560	-0.120221	1	0.175988	1.882042	-1.875429
1	0.708597	-4.825348	-1.621191	6	-1.157173	3.360096	-1.050628
6	-0.480519	-3.883537	-0.066902	1	-1.047767	4.030584	-1.900671
1	-1.350308	-3.975945	-0.725942	6	-2.002325	3.720658	0.008097
1	-0.816295	-4.173648	0.940080	6	-2.130705	2.825948	1.078998
6	3.726349	-1.176146	-0.024288	1	-2.782956	3.078747	1.912258
1	4.254290	-2.130607	-0.072630	6	-1.440404	1.613714	1.094122
6	4.052290	-0.257368	-1.200848	1	-1.564055	0.931228	1.929783
6	2.473624	1.093170	0.089226	6	-2.029899	-1.244927	-0.004762
1	1.955724	2.050186	0.142243	6	-3.244098	-1.165646	-0.013120
6	3.552037	1.925654	-2.132030	6	-4.650552	-0.954498	-0.014333
1	3.026655	2.876503	-2.079463	6	-5.157223	0.362515	-0.054523
6	4.415998	1.648448	-3.202497	1	-4.458759	1.193481	-0.086859
1	4.560703	2.388787	-3.984743	6	-6.529049	0.587938	-0.052806
6	5.089453	0.427525	-3.265575	1	-6.900821	1.610155	-0.085340
1	5.757601	0.218705	-4.096755	6	-7.444291	-0.476720	-0.011664
6	4.908487	-0.533673	-2.259234	6	-6.935373	-1.781382	0.028069
1	5.433528	-1.485059	-2.307222	1	-7.624677	-2.622047	0.060162
6	4.052213	-0.382816	1.239637	6	-5.562753	-2.024241	0.027053
6	3.369537	0.843995	1.300622	1	-5.186401	-3.042400	0.058469

6	3.374306	0.972643	-1.137134	6	-2.730645	5.043563	0.003742
6	-8.930546	-0.213565	-0.012977	1	-2.077908	5.856138	0.350316
1	-9.226859	0.403062	0.845057	1	-3.073035	5.308854	-1.002963
1	-9.238826	0.324116	-0.918666	1	-3.603973	5.021054	0.664367
1	-9.501119	-1.146786	0.032450				

Optimized geometry of 4 (CH_2Cl_2)

6	0.080576	-2.475632	-0.028332	6	5.109926	0.374887	-3.263943
6	-0.609796	-1.255291	0.007935	1	5.780069	0.152913	-4.090045
6	0.129865	-0.030875	0.024089	6	4.919582	-0.574892	-2.247300
6	1.522095	-0.098469	0.016811	1	5.438270	-1.530010	-2.282135
6	1.483275	-2.517731	-0.043540	6	4.049398	-0.379797	1.245727
6	2.203104	-1.333281	-0.017800	6	3.374244	0.852648	1.290496
6	1.971000	-3.950531	-0.062388	6	3.548098	1.711419	2.369509
1	2.304377	-4.249356	0.942279	1	3.023340	2.663205	2.402894
1	2.817426	-4.109960	-0.739168	6	4.406471	1.334734	3.414927
6	0.709071	-4.743065	-0.489419	1	4.547629	1.999862	4.262581
1	0.681122	-5.752290	-0.067923	6	5.077329	0.110309	3.370019
1	0.697474	-4.838227	-1.581801	1	5.740073	-0.175339	4.182436
6	-0.493238	-3.873120	-0.042866	6	4.899506	-0.755834	2.279137
1	-1.360305	-3.970997	-0.704773	1	5.420121	-1.709846	2.242889
1	-0.832523	-4.151311	0.966127	6	-0.587054	1.272523	0.023602
6	3.724729	-1.185985	-0.010673	6	-0.452076	2.163646	-1.049615
1	4.246806	-2.143254	-0.046944	1	0.191880	1.899264	-1.884580
6	4.059942	-0.280814	-1.195326	6	-1.143175	3.377862	-1.063396
6	2.483594	1.094151	0.073418	1	-1.024425	4.050249	-1.910117
1	1.973254	2.055410	0.113934	6	-1.989365	3.740831	-0.007345
6	3.574761	1.895701	-2.153541	6	-2.114414	2.849927	1.070099
1	3.053219	2.849095	-2.115554	1	-2.758294	3.110401	1.907496
6	4.442372	1.601051	-3.217506	6	-1.428922	1.636176	1.085529
1	4.593680	2.331431	-4.007753	1	-1.546255	0.960709	1.928058

6	-2.763045	5.037288	-0.032212
1	-2.351075	5.733943	-0.769445
1	-2.750994	5.528367	0.947466
1	-3.815162	4.862549	-0.293657
6	-2.032315	-1.225503	-0.005698
6	-3.247393	-1.147060	-0.015638
6	-4.656397	-0.944734	-0.013586
6	-5.173413	0.368412	0.013744
1	-4.483789	1.207317	0.033682
6	-6.548431	0.583273	0.021357
1	-6.927771	1.602405	0.045527
6	-7.455116	-0.489286	-0.000243
6	-6.935149	-1.792093	-0.023271
1	-7.617229	-2.638965	-0.034225
6	-5.560829	-2.023949	-0.031081
1	-5.178074	-3.040167	-0.047586
6	-8.943634	-0.242771	-0.024380
1	-9.216672	0.613037	0.602773
1	-9.499334	-1.117955	0.327470
1	-9.287851	-0.021306	-1.043578
6	3.388328	0.954015	-1.148355

Optimized geometry of 5I (gas phase)

6	0.993956	0.282984	-0.160388
6	1.229091	-2.149545	-0.102309
6	0.401112	-1.021061	-0.108431
6	0.823895	-3.602692	-0.028036
1	-0.036649	-3.837998	-0.663414
1	0.535109	-3.863285	1.001429
6	3.201477	-0.768728	-0.170966
6	2.629006	-2.027508	-0.130970
6	3.278738	-3.394728	-0.082789
1	3.650171	-3.605073	0.931544
1	4.134299	-3.493273	-0.760722
6	2.113869	-4.348016	-0.454054
1	2.208982	-5.334837	0.009973
1	2.102231	-4.492926	-1.541222
6	4.692809	-0.421678	-0.219464
1	5.330903	-1.306788	-0.190858
6	4.869798	0.411244	-1.495137
1	5.546420	0.094164	-2.282097
6	4.094892	1.497655	-1.527015
1	4.041837	2.209396	-2.344462
6	3.218581	1.663723	-0.278541
1	2.606062	2.562989	-0.305692
6	4.155006	1.606844	0.935434
1	4.142639	2.391890	1.685709
6	4.930169	0.518904	0.980104
6	5.922435	0.140212	2.033316
1	5.974292	0.893688	2.826323
1	6.928006	0.022864	1.604973
1	5.658948	-0.822889	2.493230
6	2.381102	0.376097	-0.194128
6	0.181666	2.413038	0.895565
1	0.872019	2.238050	1.716988
6	0.126076	1.490766	-0.158564
6	-0.655013	3.529932	0.915718
1	-0.600442	4.224238	1.751746
6	-1.571768	3.765558	-0.117310

6	-1.625345	2.841173	-1.169938	1	-3.950155	-3.291173	0.099431
1	-2.331949	2.996092	-1.982764	6	-5.824175	-2.229066	0.199512
6	-0.792828	1.722824	-1.191570	1	-6.416925	-3.140849	0.217171
1	-0.860945	1.013658	-2.011284	6	-6.471834	-0.987650	0.244347
6	-2.456116	4.989696	-0.110302	6	-5.678843	0.171411	0.220641
1	-3.400464	4.803964	-0.633828	1	-6.159231	1.147267	0.255977
1	-1.965966	5.835261	-0.611780	6	-4.292104	0.097868	0.154361
1	-2.691030	5.307805	0.911435	1	-3.689164	1.000960	0.136648
6	-1.012092	-1.155422	-0.032859	6	-7.976254	-0.889058	0.318101
6	-2.225719	-1.211525	0.038630	1	-8.297099	-0.365289	1.227598
6	-3.645033	-1.156136	0.109234	1	-8.441377	-1.880157	0.321342
6	-4.434702	-2.319884	0.133174	1	-8.381638	-0.331154	-0.535584

Optimized geometry of 5I (CH_2Cl_2)

6	0.998469	0.293551	-0.160928	1	5.552457	0.067713	-2.281075
6	1.211529	-2.144324	-0.110397	6	4.112716	1.483973	-1.521536
6	0.394501	-1.006740	-0.111595	1	4.068225	2.202771	-2.333566
6	0.795287	-3.595095	-0.039177	6	3.237792	1.652675	-0.272141
1	-0.067266	-3.824020	-0.674286	1	2.636828	2.559496	-0.294335
1	0.507656	-3.855810	0.990465	6	4.172107	1.578386	0.943345
6	3.196604	-0.780189	-0.177505	1	4.163837	2.358669	1.698639
6	2.613145	-2.034580	-0.140948	6	4.936546	0.481551	0.981884
6	3.251880	-3.406828	-0.097599	6	5.923988	0.083980	2.033128
1	3.623291	-3.620230	0.915534	1	5.983570	0.830940	2.831637
1	4.105155	-3.508656	-0.777087	1	6.927099	-0.043070	1.602607
6	2.078897	-4.349868	-0.468184	1	5.649349	-0.880359	2.483052
1	2.165920	-5.336514	-0.003149	6	2.386914	0.373719	-0.195513
1	2.063937	-4.494607	-1.555233	6	0.208786	2.430161	0.900786
6	4.691321	-0.449798	-0.223879	1	0.905060	2.254756	1.717063
1	5.319564	-1.341082	-0.200546	6	0.140847	1.509194	-0.153575
6	4.876660	0.388501	-1.494798	6	-0.620951	3.554088	0.926129

1	-0.555082	4.247784	1.761436	6	-4.433766	-2.313275	0.190774
6	-1.541727	3.797387	-0.101521	1	-3.941255	-3.281150	0.203735
6	-1.605413	2.874801	-1.157292	6	-5.824781	-2.233421	0.253552
1	-2.313817	3.036639	-1.967073	1	-6.407353	-3.149508	0.315270
6	-0.780676	1.751143	-1.184016	6	-6.485889	-0.997185	0.239485
1	-0.855635	1.046702	-2.007483	6	-5.704569	0.168400	0.160417
6	-2.425176	5.022020	-0.089073	1	-6.194495	1.139652	0.149417
1	-3.442318	4.783696	-0.420280	6	-4.316491	0.105604	0.096847
1	-2.037422	5.795391	-0.765421	1	-3.725344	1.014705	0.034830
1	-2.485694	5.459737	0.912666	6	-7.991270	-0.911123	0.302301
6	-1.020593	-1.130996	-0.031994	1	-8.318488	-0.284529	1.141042
6	-2.234838	-1.188639	0.043798	1	-8.442370	-1.900949	0.422910
6	-3.655996	-1.142858	0.111142	1	-8.400533	-0.463452	-0.612272

Optimized geometry of 5II (gas phase)

6	1.076060	0.117471	-0.136927	6	2.465872	0.152261	-0.156891
6	1.208630	-2.317577	0.021085	6	0.296357	2.303252	0.829396
6	0.428470	-1.157267	-0.040358	1	0.938192	2.123416	1.687785
6	0.742787	-3.748631	0.148632	6	0.256359	1.357194	-0.203286
1	-0.121142	-3.973683	-0.485893	6	-0.497291	3.450610	0.782620
1	0.434389	-3.955275	1.184625	1	-0.455827	4.163764	1.603345
6	3.238192	-1.023354	-0.079701	6	-1.355126	3.692397	-0.298098
6	2.612816	-2.254481	0.002967	6	-1.394881	2.742815	-1.329054
6	3.204563	-3.644201	0.112376	1	-2.057689	2.901322	-2.177312
1	3.557733	-3.828155	1.138253	6	-0.604796	1.595015	-1.283803
1	4.061387	-3.805000	-0.551884	1	-0.662114	0.866222	-2.087094
6	2.004580	-4.562920	-0.233368	6	-2.192877	4.947163	-0.363988
1	2.054175	-5.533350	0.270590	1	-3.140126	4.768952	-0.885106
1	1.996847	-4.750862	-1.313983	1	-1.668228	5.745591	-0.906138
6	3.351730	1.400229	-0.294627	1	-2.422453	5.327872	0.637205
1	2.772172	2.319567	-0.369987	6	-0.990262	-1.225338	0.018381

6	-2.206076	-1.216082	0.072008	1	-8.300697	-0.057329	-0.641615
6	-3.621388	-1.084870	0.117779	6	4.743561	-0.735593	-0.124234
6	-4.471038	-2.203883	0.183112	1	5.347163	-1.640717	-0.047661
1	-4.037577	-3.199419	0.201359	6	4.271523	2.533563	1.889574
6	-5.854592	-2.038386	0.224528	1	4.553161	3.464532	1.377687
1	-6.494393	-2.916506	0.275221	1	3.268879	2.696292	2.308540
6	-6.436951	-0.764433	0.202811	1	4.967318	2.374313	2.720080
6	-5.584183	0.349764	0.137854	6	4.232078	1.144465	-1.523989
1	-6.013293	1.349725	0.121319	6	4.960219	0.028982	-1.438107
6	-4.202481	0.201686	0.095667	6	4.998227	0.256388	1.018855
1	-3.552867	1.070557	0.045423	6	4.275961	1.378339	0.939397
6	-7.935006	-0.584968	0.248523	1	5.628191	-0.349400	-2.205174
1	-8.239091	0.006165	1.121716	1	4.212350	1.820682	-2.372431
1	-8.450918	-1.549168	0.301173	1	5.698243	0.022229	1.815169

Optimized geometry of 5II (CH_2Cl_2)

6	1.081505	0.127280	-0.137475	1	2.806603	2.312487	-0.358961
6	1.188020	-2.312751	0.013413	6	2.472132	0.146945	-0.157864
6	0.421168	-1.142308	-0.043458	6	0.326823	2.322047	0.830665
6	0.708811	-3.740244	0.137582	1	0.976557	2.144499	1.683439
1	-0.157799	-3.956962	-0.496310	6	0.273084	1.375108	-0.200081
1	0.402087	-3.947064	1.173949	6	-0.455934	3.478959	0.784285
6	3.231406	-1.038897	-0.086052	1	-0.398103	4.195901	1.600254
6	2.593399	-2.264415	-0.006916	6	-1.317857	3.726401	-0.291648
6	3.171672	-3.660091	0.096195	6	-1.364480	2.778838	-1.326559
1	3.525566	-3.849115	1.120420	1	-2.021229	2.948002	-2.177401
1	4.024983	-3.825325	-0.570677	6	-0.586276	1.623456	-1.281752
6	1.962010	-4.565884	-0.248655	1	-0.645526	0.900850	-2.090814
1	2.002298	-5.536104	0.255667	6	-2.186228	4.961053	-0.335186
1	1.949670	-4.752915	-1.329270	1	-3.239822	4.708798	-0.157079
6	3.372791	1.384918	-0.287987	1	-2.133393	5.451904	-1.313996

1	-1.885410	5.686999	0.426993
6	-0.999106	-1.197920	0.018742
6	-2.215627	-1.189240	0.077145
6	-3.633173	-1.066831	0.120333
6	-4.472132	-2.189657	0.248796
1	-4.031219	-3.179697	0.318975
6	-5.857746	-2.034926	0.285004
1	-6.488265	-2.915262	0.384265
6	-6.452936	-0.768669	0.196204
6	-5.610733	0.349100	0.067258
1	-6.049108	1.342153	-0.004733
6	-4.227104	0.211521	0.029411
1	-3.588348	1.084083	-0.073035
6	-7.951959	-0.600743	0.243966
1	-8.258354	-0.034832	1.132963

1	-8.460935	-1.569273	0.270748
1	-8.318010	-0.048769	-0.630330
6	4.739806	-0.770060	-0.128646
1	5.332156	-1.682177	-0.057205
6	4.303761	2.495292	1.903750
1	4.589596	3.426044	1.394316
1	3.304035	2.663754	2.326901
1	5.001494	2.325705	2.730429
6	4.250994	1.125046	-1.518409
6	4.965322	-0.000901	-1.438394
6	5.004407	0.212658	1.020746
6	4.295543	1.344595	0.947497
1	5.630214	-0.384506	-2.205690
1	4.241186	1.808964	-2.361029
1	5.700900	-0.036219	1.815757

Optimized geometry of 5III (gas phase)

6	1.000064	0.371136	-0.046682
6	1.347080	-2.038617	0.010060
6	0.466234	-0.954655	-0.021292
6	0.980582	-3.501273	0.075677
1	0.142506	-3.758436	-0.580761
1	0.672063	-3.766804	1.098257
6	3.269439	-0.572740	0.022296
6	2.745934	-1.861544	0.028519
6	3.420544	-3.224355	0.097696
1	3.759810	-3.427421	1.124186
1	4.295566	-3.316818	-0.548742
6	2.297919	-4.207008	-0.313008
1	2.409651	-5.192786	0.149748
1	2.324501	-4.346870	-1.400791

6	4.753824	-0.112396	0.055725
6	4.901172	0.760499	-1.204406
1	5.636911	0.498080	-1.959334
6	4.056407	1.789268	-1.269732
1	3.989364	2.500925	-2.086113
6	3.112549	1.877467	-0.067266
1	2.427800	2.721206	-0.123400
6	3.982929	1.884336	1.192651
1	3.873409	2.656909	1.947026
6	2.380218	0.526714	-0.027231
6	-0.003355	2.417791	1.014227
1	0.635406	2.252022	1.878245
6	0.072108	1.534324	-0.071065
6	-0.904302	3.483710	1.008914

1	-0.951772	4.148393	1.869088	1	-6.281288	-3.320837	0.065779
6	-1.756651	3.704832	-0.081052	6	-6.409594	-1.170777	0.093838
6	-1.679178	2.818748	-1.164565	6	-5.655867	0.014453	0.098245
1	-2.334403	2.962328	-2.021276	1	-6.169848	0.973467	0.118846
6	-0.782121	1.751049	-1.160803	6	-4.266032	-0.012140	0.078301
1	-0.750158	1.067726	-2.004530	1	-3.693567	0.910721	0.081787
6	-2.711232	4.874931	-0.098566	6	-7.918103	-1.123035	0.116491
1	-3.598082	4.658531	-0.704337	1	-8.286944	-0.603997	1.010318
1	-2.234966	5.768324	-0.524805	1	-8.349305	-2.129342	0.112205
1	-3.045193	5.134048	0.912217	1	-8.313052	-0.585367	-0.754928
6	-0.941454	-1.151303	0.003530	6	4.822786	0.851018	1.256239
6	-2.154078	-1.250104	0.031192	1	5.511471	0.646858	2.071151
6	-3.576037	-1.243646	0.053246	6	5.827046	-1.194202	0.133830
6	-4.326151	-2.433423	0.049114	1	5.688884	-1.841943	1.004714
1	-3.808235	-3.387745	0.030534	1	5.838158	-1.818868	-0.763899
6	-5.719351	-2.389602	0.069056	1	6.811342	-0.719023	0.219740

Optimized geometry of 5III (CH_2Cl_2)

6	-1.005871	0.381981	0.046076	1	-2.284184	-4.351922	1.409675
6	-1.330436	-2.034097	-0.006360	6	-4.756346	-0.141890	-0.052635
6	-0.460447	-0.940214	0.021294	6	-4.911543	0.733881	1.204965
6	-0.951941	-3.494245	-0.070564	1	-5.645388	0.465581	1.959730
1	-0.110853	-3.745052	0.584580	6	-4.077121	1.772460	1.266782
1	-0.645449	-3.758639	-1.093940	1	-4.017561	2.489542	2.079210
6	-3.266871	-0.585505	-0.018040	6	-3.134356	1.866889	0.063663
6	-2.731660	-1.870134	-0.021975	1	-2.460645	2.719212	0.116654
6	-3.394623	-3.238720	-0.085696	6	-4.003843	1.859556	-1.197348
1	-3.736235	-3.444999	-1.110177	1	-3.898820	2.630351	-1.954239
1	-4.265413	-3.335855	0.565068	6	-2.388069	0.523989	0.027984
6	-2.262236	-4.210954	0.322057	6	-0.029763	2.442895	-1.014520
1	-2.365815	-5.196382	-0.142392	1	-0.680840	2.284198	-1.870571

6	-0.087520	1.553337	0.066568
6	0.861733	3.518820	-1.010678
1	0.889532	4.192517	-1.864260
6	1.723819	3.740567	0.071348
6	1.656352	2.853382	1.156781
1	2.308944	3.006145	2.013845
6	0.769911	1.777222	1.154281
1	0.742610	1.098273	2.001990
6	2.713410	4.881138	0.064176
1	3.721011	4.526163	-0.190294
1	2.778165	5.360073	1.047690
1	2.437633	5.644270	-0.670870
6	0.949769	-1.126423	-0.007360
6	2.163054	-1.227316	-0.038514
6	3.586429	-1.230142	-0.055800
6	4.326639	-2.425506	-0.120808
1	3.802692	-3.375985	-0.160458
6	5.720843	-2.392235	-0.132883
1	6.274301	-3.326897	-0.182604
6	6.422128	-1.179269	-0.081965
6	5.678156	0.011281	-0.016053
1	6.199720	0.965039	0.026147
6	4.287344	-0.005026	-0.003060
1	3.724928	0.922660	0.050616
6	7.930618	-1.143545	-0.106996
1	8.299442	-0.678302	-1.030176
1	8.354207	-2.150955	-0.047293
1	8.328439	-0.556887	0.729820
6	-4.833645	0.816842	-1.257049
1	-5.519870	0.600797	-2.071022
6	-5.818378	-1.234981	-0.127282
1	-5.673766	-1.882568	-0.996896
1	-5.823519	-1.855900	0.772714
1	-6.806486	-0.768705	-0.214898

Optimized geometry of 5IV (gas phase)

6	1.170944	0.192912	0.010002
6	1.443695	-2.240791	0.029885
6	0.603902	-1.125855	0.021726
6	1.067448	-3.702594	0.057622
1	0.229954	-3.939859	-0.607150
1	0.756281	-3.993973	1.072152
6	3.386182	-0.815888	0.037294
6	2.838492	-2.084761	0.037402
6	3.518457	-3.438463	0.071639
1	3.870313	-3.662953	1.089833
1	4.391412	-3.504021	-0.587631
6	2.385162	-4.409283	-0.349587
1	2.491036	-5.403346	0.096232
1	2.404867	-4.531397	-1.439420
6	5.107100	0.377375	-1.189704
1	5.805067	0.081183	-1.966039
6	4.341677	1.468319	-1.208323
1	4.321131	2.208780	-2.002789
6	3.414086	1.638474	0.010408
6	4.313769	1.496679	1.253564
1	4.275751	2.255485	2.029884
6	2.557091	0.332569	0.017701
6	-0.317460	1.847959	1.180238
1	0.037864	1.454623	2.129181

6	0.196649	1.324518	-0.011339	1	-6.273975	-3.119633	0.065385
6	-1.286454	2.851453	1.160000	6	-6.270282	-0.966007	0.023686
1	-1.674848	3.239582	2.099344	6	-5.445270	0.170593	0.000660
6	-1.777620	3.358371	-0.050874	1	-5.899548	1.159404	-0.017738
6	-1.271428	2.819356	-1.241492	6	-4.059620	0.059055	0.000682
1	-1.647863	3.182280	-2.195667	1	-3.431600	0.944457	-0.017261
6	-0.302300	1.815894	-1.222796	6	-7.773222	-0.825729	0.022587
1	0.065028	1.397617	-2.156383	1	-8.120878	-0.253840	0.892354
6	-2.809870	4.460643	-0.072056	1	-8.265289	-1.803454	0.046733
1	-3.446036	4.395593	-0.961674	1	-8.123490	-0.296289	-0.872681
1	-2.333075	5.450130	-0.082992	6	5.078493	0.405297	1.277771
1	-3.456224	4.420109	0.811654	1	5.758722	0.127235	2.076226
6	-0.810987	-1.267813	0.021531	6	4.882865	-0.491882	0.051666
6	-2.027384	-1.303290	0.024666	1	5.503432	-1.388200	0.070083
6	-3.446459	-1.212726	0.024420	6	2.707325	2.991862	-0.013689
6	-4.267838	-2.354045	0.047796	1	2.086112	3.115124	-0.903630
1	-3.809336	-3.338322	0.066551	1	2.068995	3.137913	0.860488
6	-5.655960	-2.224785	0.047145	1	3.469108	3.781261	-0.016439

Optimized geometry of 5IV (CH_2Cl_2)

6	1.174147	-0.198983	-0.008484	6	2.384007	4.409141	0.345976
6	1.443410	2.238650	-0.030070	1	2.488193	5.400892	-0.104374
6	0.606327	1.120621	-0.021833	1	2.403093	4.534877	1.435296
6	1.066661	3.700855	-0.059890	6	5.111192	-0.371717	1.191217
1	0.229771	3.940667	0.604878	1	5.809265	-0.070998	1.965811
1	0.757156	3.990386	-1.075324	6	4.347947	-1.465247	1.211773
6	3.388199	0.815668	-0.036162	1	4.328788	-2.205490	2.006654
6	2.839173	2.084640	-0.037298	6	3.420397	-1.639294	-0.007019
6	3.518041	3.438671	-0.072883	6	4.318625	-1.496179	-1.251620
1	3.869614	3.660179	-1.091312	1	4.280971	-2.256175	-2.026971
1	4.390472	3.504106	0.586191	6	2.560898	-0.335473	-0.015441

6	-0.309891	-1.856958	-1.180957	6	-5.657048	2.227553	-0.046733
1	0.054988	-1.473300	-2.130400	1	-6.273372	3.123249	-0.064437
6	0.200044	-1.331393	0.011975	6	-6.273467	0.968674	-0.020742
6	-1.285280	-2.855126	-1.162822	6	-5.449343	-0.169684	0.001793
1	-1.669945	-3.244664	-2.102961	1	-5.904058	-1.157838	0.022348
6	-1.786396	-3.355463	0.047521	6	-4.062939	-0.059229	-0.001123
6	-1.279958	-2.818112	1.239616	1	-3.438516	-0.947540	0.016852
1	-1.660427	-3.178555	2.192964	6	5.081035	-0.402093	-1.276919
6	-0.304529	-1.819848	1.222825	1	5.760323	-0.120969	-2.075204
1	0.064470	-1.407207	2.158417	6	4.885554	0.495949	-0.051179
6	-2.828515	-4.448297	0.066527	1	5.504320	1.392553	-0.070710
1	-3.464484	-4.377343	0.955452	6	2.715664	-2.993518	0.019076
1	-2.359451	-5.441314	0.078288	1	2.095613	-3.114653	0.910143
1	-3.471243	-4.401652	-0.819154	1	2.079979	-3.140859	-0.856790
6	-0.809858	1.261226	-0.023765	1	3.478447	-3.781527	0.023896
6	-2.027044	1.301620	-0.028655	6	-7.776233	0.830051	-0.017336
6	-3.447657	1.212490	-0.027181	1	-8.124527	0.268335	-0.893129
6	-4.268073	2.355636	-0.050165	1	-8.122375	0.287091	0.870960
1	-3.810698	3.340512	-0.070215	1	-8.266905	1.808263	-0.027305

Optimized geometry of 6 (gas phase)

6	1.230510	0.296453	0.027622	1	4.365648	-3.500585	0.519350
6	0.627619	-1.004821	0.000097	1	3.819673	-3.610470	-1.153876
6	1.030184	-3.592111	-0.090803	6	2.847813	-2.028498	-0.057204
1	0.703313	-3.851694	-1.108934	6	3.429872	-0.773113	-0.041611
1	0.193240	-3.823303	0.576892	6	4.927332	-0.440529	-0.050535
6	1.446253	-2.140712	-0.033733	1	5.555800	-1.329283	-0.102434
6	2.330737	-4.345171	0.287031	6	5.118209	0.508241	-1.243290
1	2.358020	-4.491802	1.373573	1	5.801784	0.258929	-2.048389
1	2.401505	-5.330812	-0.182801	6	4.356885	1.604109	-1.180020
6	3.486902	-3.399390	-0.126915	1	4.320774	2.391911	-1.925694

6	3.465890	1.662475	0.068672
1	2.861573	2.565502	0.123465
6	4.386111	1.482071	1.284434
1	4.367115	2.194126	2.103157
6	5.149940	0.388265	1.223263
1	5.851856	0.062494	1.984075
6	2.619169	0.378635	0.010201
6	0.370392	1.510010	0.052787
6	-0.511401	1.750521	1.117488
1	-0.550872	1.048387	1.945351
6	-1.336306	2.874583	1.123419
1	-2.008667	3.039131	1.962801
6	-1.315770	3.795903	0.065027
6	-0.428919	3.557926	-0.993468
1	-0.388541	4.257162	-1.825768
6	0.400795	2.433992	-1.000892
1	1.071536	2.263818	-1.839358
6	-2.240326	4.989773	0.058453
1	-1.872344	5.776103	-0.608759
1	-2.350694	5.415283	1.062105
1	-3.244110	4.707859	-0.286706
6	-0.789267	-1.131617	-0.023561
6	-2.005421	-1.191970	-0.049788
6	-3.428208	-1.147360	-0.058120
6	-4.207825	-2.317737	-0.118526
1	-3.715649	-3.284869	-0.162421
6	-5.600223	-2.238770	-0.120275
1	-6.184401	-3.154761	-0.166359
6	-6.260972	-1.003484	-0.063451
6	-5.477864	0.161898	-0.002265
1	-5.967536	1.132208	0.044557
6	-4.088240	0.099951	0.000461
1	-3.495461	1.008730	0.050639
6	-7.767611	-0.918284	-0.078052
1	-8.127720	-0.449474	-1.002908
1	-8.139776	-0.311396	0.756088
1	-8.223620	-1.910632	-0.006318

Optimized geometry of 6 (CH_2Cl_2)

6	1.230531	0.296487	0.027671
6	0.627582	-1.004756	0.000069
6	1.030033	-3.592062	-0.090783
1	0.703257	-3.851619	-1.108950
1	0.193006	-3.823218	0.576821
6	1.446169	-2.140686	-0.033659
6	2.330506	-4.345195	0.287186
1	2.357645	-4.491867	1.373726
1	2.401283	-5.330823	-0.182670
6	3.486773	-3.399459	-0.126584
1	4.365416	-3.500697	0.519816
1	3.819694	-3.610542	-1.153495
6	2.847736	-2.028540	-0.056951
6	3.429854	-0.773184	-0.041281
6	4.927330	-0.440676	-0.050068
1	5.555761	-1.329461	-0.101873
6	5.118361	0.508043	-1.242842
1	5.802005	0.258673	-2.047865
6	4.357090	1.603955	-1.179674
1	4.321096	2.391741	-1.925371

6	3.465974	1.662407	0.068931	6	-3.428247	-1.147428	-0.058523
1	2.861692	2.565461	0.123634	6	-4.207775	-2.317632	-0.121770
6	4.386070	1.481998	1.284786	1	-3.715589	-3.284641	-0.168152
1	4.367037	2.194087	2.103480	6	-5.600247	-2.238675	-0.123935
6	5.149862	0.388160	1.223717	1	-6.184346	-3.154559	-0.172829
1	5.851703	0.062391	1.984598	6	-6.261017	-1.003613	-0.064318
6	2.619199	0.378604	0.010419	6	-5.477912	0.161739	-0.000908
6	0.370463	1.510081	0.052671	1	-5.967643	1.131972	0.047110
6	-0.511378	1.750772	1.117228	6	-4.088360	0.099815	0.002192
1	-0.550941	1.048784	1.945213	1	-3.495594	1.008525	0.053746
6	-1.336249	2.874923	1.122929	6	-7.767692	-0.918186	-0.074045
1	-2.008620	3.039640	1.962262	1	-8.128647	-0.398113	-0.970490
6	-1.315604	3.796053	0.064427	1	-8.139437	-0.359173	0.793375
6	-0.428598	3.557909	-0.993964	1	-8.223353	-1.913099	-0.056566
1	-0.388065	4.257068	-1.826330	6	-2.240360	4.989756	0.057067
6	0.401027	2.433975	-1.001149	1	-1.866116	5.780790	-0.601087
1	1.071903	2.263685	-1.839483	1	-3.240552	4.710021	-0.300139
6	-0.789306	-1.131514	-0.023764	1	-2.360767	5.408223	1.062480
6	-2.005452	-1.191948	-0.050235				

Optimized geometry of 8 (gas phase)

6	8.823482	-3.985368	-0.233283	6	5.512597	-2.087053	-0.173896
1	9.851498	-3.614008	-0.171270	1	4.448817	-2.298444	-0.227740
1	8.725569	-4.547473	-1.170700	6	6.445990	-3.115091	-0.235028
1	8.674198	-4.698366	0.587543	1	6.100486	-4.141716	-0.339215
6	7.824879	-2.855617	-0.165521	6	4.961729	0.284442	0.020629
6	8.239725	-1.524065	-0.032675	6	4.027834	1.062967	0.064906
1	9.302422	-1.299333	0.022576	6	2.869811	1.886992	0.097103
6	7.317023	-0.480959	0.029670	6	2.982515	3.295566	0.188635
1	7.656232	0.545596	0.132184	6	4.260662	4.091131	0.273605
6	5.938215	-0.747071	-0.039712	1	4.748150	4.128948	-0.712363

1	4.988189	3.651238	0.964262
6	3.762563	5.484460	0.717048
1	4.379287	6.303395	0.333485
1	3.780011	5.541782	1.812202
6	2.295794	5.563993	0.228912
1	1.678224	6.170257	0.895730
1	2.240998	6.027399	-0.767102
6	1.866593	4.105761	0.158496
6	0.558192	3.552918	0.046528
6	0.423387	2.107191	0.026610
6	1.601854	1.295267	0.025106
6	1.560781	-0.190627	-0.086141
6	1.444246	-0.804875	-1.337586
1	1.367189	-0.187285	-2.228407
6	1.390744	-2.193599	-1.442619
1	1.272427	-2.653582	-2.421354
6	1.460177	-3.006609	-0.304273
6	1.639669	-2.385907	0.939466
1	1.718037	-2.998375	1.835182
6	1.696599	-0.998544	1.048390
1	1.821143	-0.532344	2.021932
6	1.281858	-4.500532	-0.405953
1	1.636284	-4.884648	-1.368983
1	0.218917	-4.761277	-0.315442
1	1.818640	-5.026208	0.391711
6	-3.195689	-6.541149	0.365876
1	-3.752737	-6.765698	1.282896
1	-2.316026	-7.198965	0.351841
1	-3.824258	-6.814066	-0.488206
6	-2.789418	-5.088661	0.308746
6	-2.880062	-4.354572	-0.881344
1	-3.272522	-4.833125	-1.775906
6	-2.478614	-3.020940	-0.942541

1	-2.557203	-2.466062	-1.872470
6	-1.968212	-2.375144	0.198792
6	-1.877752	-3.108677	1.398300
1	-1.484349	-2.621416	2.284795
6	-2.282887	-4.438837	1.445456
1	-2.205310	-4.985887	2.382816
6	-1.560481	-1.013645	0.141983
6	-1.160485	0.133162	0.080476
6	-0.900311	1.530857	0.010916
6	-0.621384	4.346305	-0.050234
6	-0.763658	5.860236	-0.083077
1	-0.035385	6.353588	-0.730856
1	-0.628543	6.277979	0.925484
6	-2.214180	6.076013	-0.577682
1	-2.665939	6.987531	-0.173805
1	-2.211307	6.164261	-1.670886
6	-2.973191	4.793102	-0.173644
1	-3.452887	4.897852	0.811208
1	-3.764929	4.518365	-0.878708
6	-1.871897	3.764690	-0.105101
6	-2.039026	2.361722	-0.051865
6	-3.338581	1.793151	-0.061014
6	-4.432549	1.259702	-0.058701
6	-5.654388	0.531838	-0.051516
6	-6.901896	1.176950	-0.128902
1	-6.936746	2.260354	-0.195106
6	-8.081586	0.434414	-0.121196
1	-9.036613	0.951207	-0.182226
6	-8.060838	-0.963904	-0.036363
6	-6.812326	-1.603022	0.041390
1	-6.768994	-2.688169	0.108427
6	-5.627285	-0.877156	0.034922
1	-4.670822	-1.385953	0.095595

6	-9.337740	-1.768415	-0.025790
1	-9.434640	-2.348213	0.900981
1	-9.364725	-2.483352	-0.857848
1	-10.217683	-1.122540	-0.110049

Optimized geometry of 8 (CH_2Cl_2)

6	8.989685	-3.792666	-0.287328
1	10.001975	-3.382212	-0.220391
1	8.909920	-4.344041	-1.232530
1	8.865792	-4.522802	0.522073
6	7.948298	-2.703924	-0.202969
6	8.311947	-1.356261	-0.071650
1	9.365131	-1.089596	-0.028260
6	7.349675	-0.349617	0.004017
1	7.651653	0.688680	0.105047
6	5.980813	-0.670154	-0.049968
6	5.606984	-2.026403	-0.181976
1	4.552003	-2.281978	-0.222671
6	6.579129	-3.017884	-0.256219
1	6.273102	-4.056827	-0.357919
6	4.966458	0.325022	0.023051
6	4.009096	1.074951	0.080365
6	2.838359	1.882425	0.123885
6	2.937771	3.290927	0.239332
6	4.206398	4.099030	0.350549
1	4.705513	4.153683	-0.628616
1	4.929176	3.660871	1.047311
6	3.687814	5.482431	0.801711
1	4.301008	6.310041	0.432722
1	3.690349	5.528882	1.897335
6	2.227574	5.552732	0.293827
1	1.593914	6.144428	0.958034
1	2.181958	6.024012	-0.698259
6	1.814376	4.091422	0.202326
6	0.513121	3.526245	0.067581
6	0.391330	2.078980	0.039330
6	1.577221	1.277295	0.031886
6	1.552692	-0.205248	-0.121297
6	1.396842	-0.783688	-1.385911
1	1.269074	-0.142154	-2.254065
6	1.371161	-2.170451	-1.532967
1	1.224793	-2.602264	-2.520669
6	1.505792	-3.016487	-0.424494
6	1.717541	-2.429625	0.831340
1	1.842192	-3.066721	1.704147
6	1.747441	-1.045194	0.981727
1	1.897531	-0.607932	1.965274
6	1.364234	-4.511284	-0.565046
1	1.644952	-4.850327	-1.567991
1	0.321193	-4.809285	-0.392615
1	1.981961	-5.045223	0.165517
6	-3.077528	-6.617242	0.489576
1	-3.557173	-6.861232	1.444498
1	-2.187533	-7.254685	0.403069
1	-3.762924	-6.893067	-0.318046
6	-2.704490	-5.156839	0.419008
6	-2.961826	-4.394549	-0.728658
1	-3.458976	-4.857474	-1.577876
6	-2.593295	-3.051480	-0.803833
1	-2.800466	-2.477641	-1.702203

6	-1.947191	-2.426412	0.279173
6	-1.690496	-3.187765	1.437274
1	-1.189162	-2.719401	2.278253
6	-2.065290	-4.526138	1.499504
1	-1.856249	-5.095711	2.402399
6	-1.561657	-1.058144	0.202731
6	-1.177047	0.093190	0.115998
6	-0.928259	1.491782	0.021778
6	-0.670962	4.311459	-0.045675
6	-0.823291	5.823913	-0.082326
1	-0.088088	6.320381	-0.719127
1	-0.706886	6.241502	0.927972
6	-2.267157	6.028957	-0.599693
6	-2.731394	6.935704	-0.200582
1	-2.248569	6.117698	-1.692501
6	-3.023694	4.741067	-0.206830
1	-3.518157	4.844241	0.770658
1	-3.802136	4.461957	-0.924848
6	-1.916605	3.719953	-0.119386
6	-2.071993	2.315351	-0.059732
6	-3.369522	1.739343	-0.074994
6	-4.468072	1.213507	-0.080652
6	-5.705122	0.509751	-0.079472
6	-6.937261	1.182938	-0.178534
1	-6.948827	2.265985	-0.257630
6	-8.133804	0.466829	-0.174549
1	-9.075568	1.004633	-0.251857
6	-8.145983	-0.931490	-0.072572
6	-6.912577	-1.598255	0.027468
1	-6.895874	-2.682818	0.109043
6	-5.710986	-0.898842	0.024446
1	-4.767386	-1.429920	0.101032
6	-9.440259	-1.706966	-0.074265
1	-9.522839	-2.342027	0.816180
1	-9.503138	-2.368272	-0.947750
1	-10.306150	-1.038050	-0.095485

Optimized geometry of 9 (gas phase)

6	0.281647	0.617123	0.005456
1	0.737787	1.602048	0.010047
6	1.119542	-0.519798	0.002004
6	0.515033	-1.790317	0.012732
6	1.175727	-3.147421	0.033019
1	1.554052	-3.365944	1.042762
1	2.034897	-3.212310	-0.643211
6	0.019811	-4.103898	-0.357606
1	0.110577	-5.090127	0.107749
1	0.025762	-4.249879	-1.444378
6	-1.281105	-3.365446	0.049221
1	-2.121944	-3.576648	-0.619948
1	-1.606110	-3.649511	1.061194
6	-0.872198	-1.911034	0.029795
6	-1.708935	-0.780540	0.040970
6	-1.109195	0.513559	0.029950
6	-1.935354	1.747522	0.027498
6	-1.718644	2.747480	-0.933692
1	-0.955118	2.598686	-1.692862
6	-2.486614	3.910629	-0.943116
1	-2.304307	4.664505	-1.706408
6	-3.493952	4.121284	0.009273

6	-3.702046	3.125849	0.972356
1	-4.469580	3.267148	1.730331
6	-2.939350	1.957795	0.983095
1	-3.120332	1.205837	1.744479
6	-4.318674	5.386158	-0.004471
1	-5.089486	5.367091	0.772874
1	-4.818844	5.526030	-0.970893
1	-3.693023	6.271372	0.166979
6	-3.119240	-0.937888	0.022998
6	-4.330937	-1.052339	0.002391
6	-5.752291	-1.069789	-0.008697
6	-6.465975	0.145627	-0.081113
1	-5.912612	1.078593	-0.133468
6	-7.856366	0.144470	-0.084574
1	-8.389818	1.091119	-0.139985
6	-8.585485	-1.053836	-0.018409
6	-7.871170	-2.257458	0.055004
1	-8.414853	-3.197733	0.110847
6	-6.478080	-2.273392	0.059506
1	-5.940690	-3.215121	0.119115
6	-10.094250	-1.039946	-0.046303
1	-10.508340	-2.011724	0.241548
1	-10.468944	-0.804269	-1.051316
1	-10.499737	-0.283239	0.635640
6	2.530391	-0.383852	-0.002585
6	3.749526	-0.286039	-0.003310
6	5.103606	-0.172036	-0.001493
6	6.321830	-0.069234	0.002254
6	7.736504	0.051611	0.011331
6	8.351186	1.315614	0.122933
1	7.729140	2.202124	0.199489
6	9.738193	1.425645	0.131500
1	10.193311	2.409826	0.216170
6	10.560132	0.293120	0.031993
6	9.944031	-0.961865	-0.083764
1	10.560758	-1.853688	-0.168861
6	8.558465	-1.089053	-0.093479
1	8.097392	-2.067671	-0.185315
6	12.063026	0.418469	0.078254
1	12.549432	-0.386528	-0.483030
1	12.429705	0.364563	1.112360
1	12.398180	1.374827	-0.337422

Optimized geometry of 9 (CH_2Cl_2)

6	0.284542	0.63777	0.008548
1	0.747245	1.619646	0.009412
6	1.114171	-0.505871	0.00917
6	0.503654	-1.773757	0.020835
6	1.154963	-3.135234	0.047571
1	1.527114	-3.352248	1.059666
1	2.014697	-3.209458	-0.626891
6	-0.005685	-4.085793	-0.342666
1	0.076892	-5.068447	0.130506
1	0.003295	-4.237887	-1.428307
6	-1.302951	-3.337131	0.056097
1	-2.141556	-3.54666	-0.616185
1	-1.632388	-3.614698	1.068129
6	-0.88513	-1.885255	0.033465
6	-1.713986	-0.748214	0.040309
6	-1.107296	0.54346	0.029704

6	-1.924634	1.783688	0.021763	6	-6.457094	-2.293917	-0.020818
6	-1.685065	2.787759	-0.931077	1	-5.900497	-3.226346	-0.02713
1	-0.909559	2.640035	-1.678176	6	-10.100348	-1.136722	-0.036597
6	-2.444323	3.957312	-0.945876	1	-10.491801	-2.121302	0.238017
1	-2.243985	4.713921	-1.701574	1	-10.477766	-0.897249	-1.03962
6	-3.466061	4.170554	-0.008049	1	-10.520847	-0.395262	0.652102
6	-3.695465	3.171879	0.947922	6	2.52643	-0.377207	0.006926
1	-4.473923	3.314466	1.694029	6	3.746618	-0.284082	0.006433
6	-2.941023	1.997789	0.964362	6	5.101934	-0.176552	0.00735
1	-3.138864	1.245694	1.721423	6	6.32152	-0.079694	0.009263
6	-4.286566	5.437781	-0.033435	6	7.7376	0.031677	0.014874
1	-5.035173	5.442615	0.765069	6	8.360066	1.291456	0.140749
1	-4.812201	5.550701	-0.989759	1	7.746225	2.182374	0.231444
1	-3.652618	6.324256	0.09233	6	9.748136	1.391611	0.145268
6	-3.125486	-0.901655	0.018492	1	10.210059	2.371207	0.240867
6	-4.336776	-1.02629	-0.003811	6	10.562047	0.253589	0.027665
6	-5.75848	-1.071224	-0.010362	6	9.937093	-0.99668	-0.102193
6	-6.49893	0.13059	0.002363	1	10.546989	-1.891251	-0.201478
1	-5.968812	1.078373	0.014318	6	8.550185	-1.114743	-0.108108
6	-7.889701	0.099314	0.003163	1	8.084079	-2.089912	-0.210888
1	-8.443519	1.035279	0.015135	6	12.06535	0.369002	0.068559
6	-8.592021	-1.117463	-0.01023	1	12.543861	-0.446933	-0.482728
6	-7.850571	-2.308243	-0.019528	1	12.430757	0.324597	1.103344
1	-8.37279	-3.2619	-0.025114	1	12.404788	1.319355	-0.356542

Optimized geometry of 11 (gas phase)

6	-2.140807	-1.007925	0.306006	6	2.186841	1.146300	0.052269
6	-1.478179	-2.251164	0.318600	6	2.883061	-0.101715	0.064852
6	-0.091912	-2.369528	0.369235	6	3.063232	2.217369	-0.205780
6	0.725375	-1.212079	0.320560	6	2.498934	3.475316	-0.357625
6	2.176729	-1.271900	0.203640	6	1.104143	3.656126	-0.172291

6	0.240355	2.589549	0.172146	1	-4.503007	5.647158	0.868825
6	-1.174507	2.696130	0.526605	1	-2.947001	6.202768	1.505497
6	-1.952340	1.507613	0.675806	6	4.308174	0.224037	-0.157640
6	-1.370029	0.180108	0.438960	6	5.456809	-0.559260	-0.230993
6	0.046816	0.068896	0.356817	1	5.408866	-1.635296	-0.110716
6	0.812119	1.277345	0.225793	6	6.693161	0.059899	-0.463383
6	-2.148029	-3.602836	0.240733	1	7.588434	-0.554947	-0.519703
1	-2.440581	-3.817849	-0.798306	6	6.809059	1.447093	-0.622173
1	-3.062546	-3.656509	0.840081	6	5.648721	2.239018	-0.549643
6	-1.040336	-4.558085	0.725690	1	5.728392	3.317093	-0.665217
1	-1.111465	-5.558295	0.286376	6	4.412687	1.643017	-0.324530
1	-1.109891	-4.668781	1.815073	6	8.153090	2.093697	-0.860935
6	0.282781	-3.846008	0.368408	1	8.415185	2.780569	-0.045754
1	1.086357	-4.095060	1.062441	1	8.155638	2.678641	-1.789426
1	0.633097	-4.147516	-0.627759	1	8.948412	1.344950	-0.934729
6	3.174072	4.771735	-0.735815	6	2.937349	-2.553563	0.186470
1	3.950910	4.638148	-1.496407	6	3.184297	-3.225204	-1.015628
1	3.660171	5.221501	0.143581	1	2.774800	-2.827332	-1.940435
6	1.997682	5.639397	-1.227120	6	3.955944	-4.387457	-1.036120
1	2.163079	6.712150	-1.084586	1	4.138693	-4.891527	-1.982752
1	1.843717	5.464964	-2.299009	6	4.506800	-4.911546	0.141256
6	0.772386	5.116555	-0.445248	6	4.270286	-4.224153	1.340318
1	0.662148	5.675687	0.496576	1	4.700207	-4.599956	2.266383
1	-0.158606	5.245531	-1.005993	6	3.500744	-3.060891	1.363520
6	-1.777308	3.936914	0.831987	1	3.337264	-2.534240	2.300104
1	-1.187986	4.838841	0.775728	6	5.314842	-6.187071	0.122885
6	-3.091534	4.057168	1.264961	1	4.676187	-7.061325	0.307748
6	-3.833894	2.878316	1.440104	1	6.090462	-6.180461	0.896535
1	-4.850130	2.928074	1.825747	1	5.802545	-6.337402	-0.846278
6	-3.270274	1.644559	1.165981	6	-3.541955	-1.039442	0.075961
1	-3.854630	0.759883	1.369958	6	-4.726989	-1.159185	-0.180411
6	-3.694904	5.405721	1.571821	6	-6.121198	-1.172720	-0.456546
1	-4.126868	5.430034	2.579998	6	-6.847844	-2.376515	-0.513329

1	-6.330713	-3.316735	-0.346851	6	-6.809924	0.039660	-0.676499
6	-8.215779	-2.363525	-0.778419	1	-6.256913	0.973673	-0.640574
1	-8.759387	-3.304635	-0.817437	6	-10.384898	-1.148543	-1.288052
6	-8.904260	-1.162727	-0.996380	1	-10.930405	-0.546817	-0.550251
6	-8.174757	0.036206	-0.940546	1	-10.804539	-2.159494	-1.272550
1	-8.687311	0.981096	-1.108831	1	-10.591549	-0.714797	-2.274744

Optimized geometry of 11 (CH_2Cl_2)

6	-2.156954	-0.957104	0.297126	1	1.024764	-4.086368	1.067758
6	-1.512384	-2.210599	0.313587	1	0.573002	-4.137073	-0.624469
6	-0.127435	-2.348604	0.366522	6	3.250679	4.745727	-0.733850
6	0.706611	-1.202944	0.316609	1	4.029032	4.601799	-1.490555
6	2.157597	-1.285842	0.203520	1	3.738910	5.184373	0.149392
6	2.203722	1.133535	0.049023	6	2.090489	5.633295	-1.227660
6	2.880752	-0.125329	0.065478	1	2.272331	6.701751	-1.077227
6	3.096944	2.191996	-0.206571	1	1.939332	5.466836	-2.301107
6	2.553813	3.459615	-0.360933	6	0.853008	5.127907	-0.453767
6	1.160253	3.661913	-0.181419	1	0.746468	5.685420	0.488717
6	0.279416	2.607605	0.160212	1	-0.072202	5.272872	-1.019765
6	-1.135832	2.735819	0.508928	6	-1.721249	3.987146	0.807418
6	-1.932379	1.558660	0.657925	1	-1.119127	4.880282	0.749249
6	-1.369331	0.220976	0.427209	6	-3.036570	4.129376	1.233341
6	0.046484	0.088668	0.348363	6	-3.798034	2.961752	1.407832
6	0.830225	1.285886	0.217462	1	-4.816298	3.028309	1.784570
6	-2.200562	-3.553432	0.238891	6	-3.251323	1.718247	1.140272
1	-2.493056	-3.767682	-0.800126	1	-3.852529	0.844280	1.341432
1	-3.116249	-3.593973	0.837226	6	-3.621344	5.488040	1.530514
6	-1.107058	-4.522851	0.727693	1	-4.064627	5.519867	2.533218
1	-1.191505	-5.521411	0.288023	1	-4.418715	5.737536	0.818529
1	-1.180049	-4.631097	1.816937	1	-2.860310	6.272456	1.469193
6	0.226090	-3.829970	0.370867	6	4.311417	0.179213	-0.152729

6	5.450191	-0.621006	-0.218047	1	3.292636	-2.563351	2.307572
1	5.386128	-1.695786	-0.092967	6	5.249931	-6.230626	0.142959
6	6.697192	-0.020394	-0.444824	1	4.729617	-7.033990	0.679874
1	7.583867	-0.648118	-0.492550	1	6.219638	-6.089391	0.635019
6	6.833991	1.365937	-0.607928	1	5.434911	-6.576361	-0.878942
6	5.684720	2.175211	-0.540289	6	-3.559572	-0.973095	0.069053
1	5.781253	3.251669	-0.655181	6	-4.746394	-1.090284	-0.182851
6	4.438194	1.596707	-0.320771	6	-6.142952	-1.126417	-0.449759
6	8.185574	1.988932	-0.865594	6	-6.834160	-2.351909	-0.555065
1	8.358433	2.850996	-0.210429	1	-6.287694	-3.282497	-0.433874
1	8.264082	2.349247	-1.899793	6	-8.201755	-2.369310	-0.809058
1	8.994192	1.269556	-0.701981	1	-8.717059	-3.324065	-0.885422
6	2.900282	-2.578289	0.189845	6	-8.929817	-1.178085	-0.969158
6	3.137620	-3.256312	-1.009534	6	-8.238039	0.037370	-0.864056
1	2.729030	-2.861814	-1.936212	1	-8.780073	0.972221	-0.984153
6	3.894779	-4.430170	-1.025567	6	-6.868305	0.071388	-0.609269
1	4.067422	-4.941579	-1.969746	1	-6.348291	1.021722	-0.532878
6	4.439730	-4.956340	0.153054	6	-10.410790	-1.215164	-1.255057
6	4.208348	-4.263176	1.352033	1	-10.839873	-0.208511	-1.265732
1	4.628605	-4.644257	2.280351	1	-10.945777	-1.805662	-0.501495
6	3.454634	-3.090395	1.370855	1	-10.612890	-1.677857	-2.229462

Computational details 2

All geometry optimizations were performed with the Gaussian 09 Rev. E[7a] program package employing UB3LYP/6-311++G(d,p).[7a] Grimme's dispersion correction D3[10] was applied. To investigate the influence of computational approaches on the geometries, we performed single point energy calculations with MP2, SCS-MP2, CC2, SCS-CC2 and CCSD/aug-cc-pVD.[11] employing the Turbomole 7.0.1[12] program package. M06-2X-D3/aug-cc-pVDZ[13] calculations were conducted with the Gaussian 09 Rev. E[7a] package.

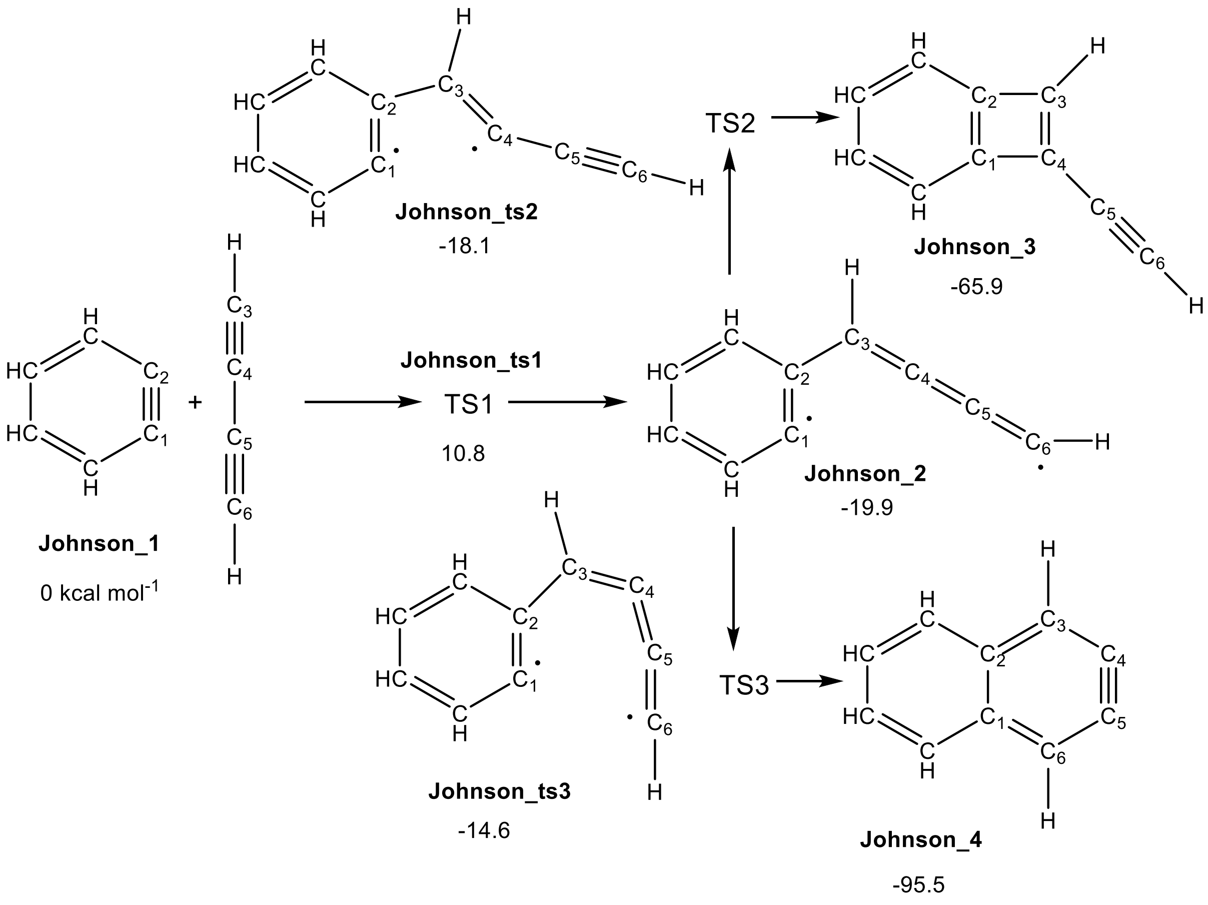

Figure S17. Detailed reaction mechanism for Step 2 (Figure 10 main text) according to Cahill and Johnson.[14]

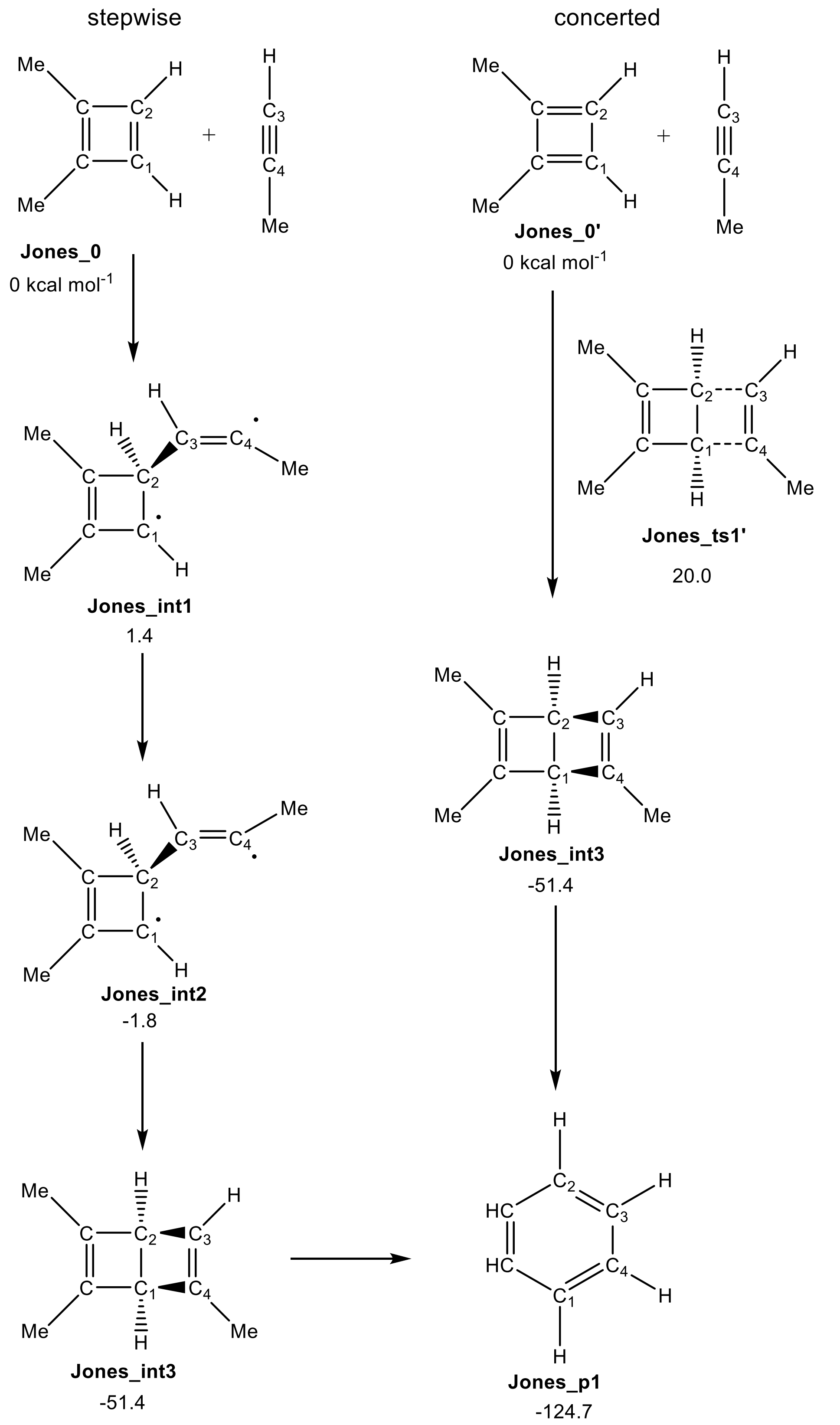

Figure S18. Mechanism computed by Jones and Krebs[15] for the reaction of cyclobutadiene and acetylene. The energies, from Jones and Krebs, were obtained by (U)B3LYP-D3/6-31G(d) computations.[15]

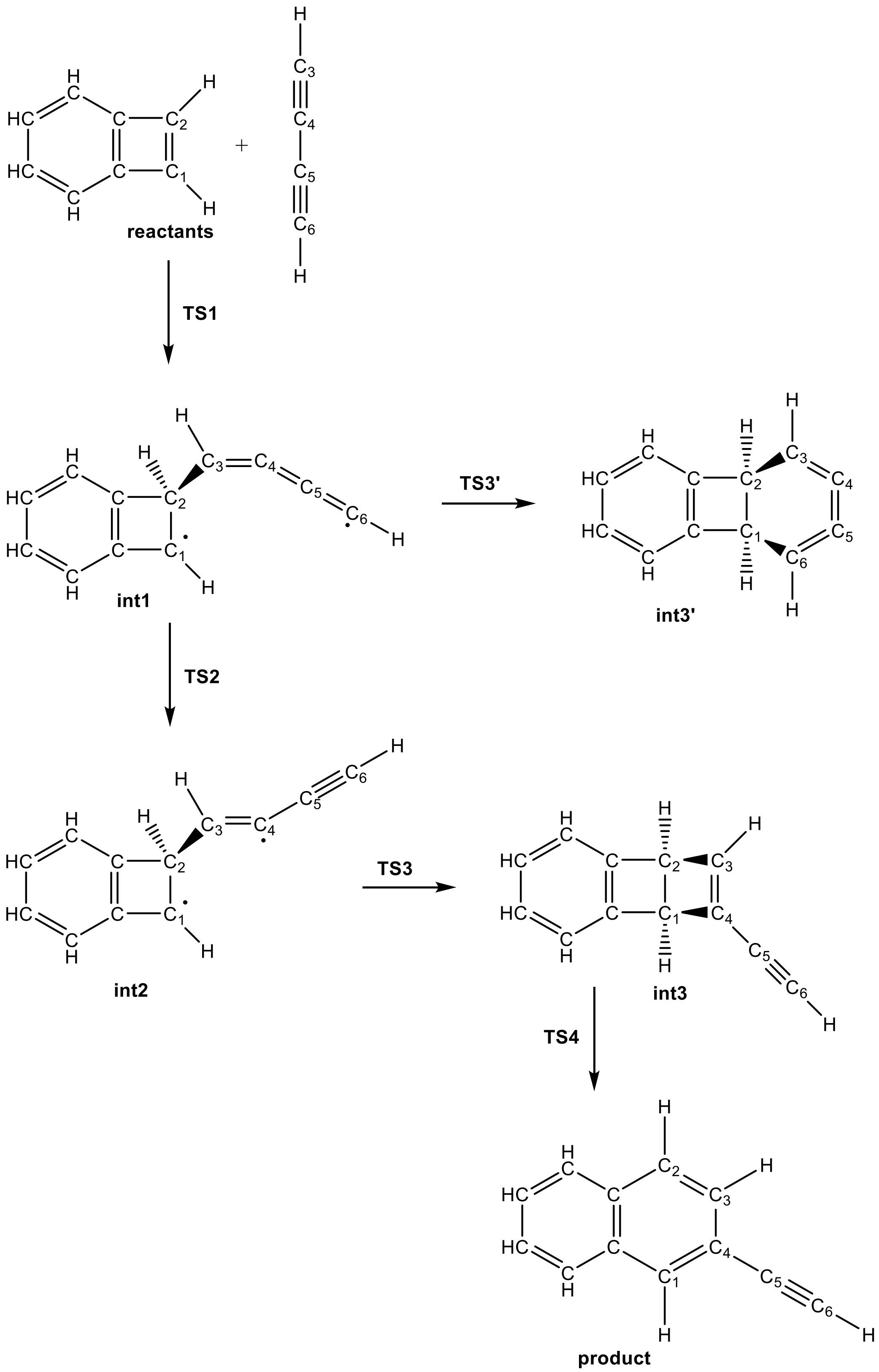

Figure S19. Detailed reaction mechanism for Steps 3 and 4 (Figure 10 main text), modelled by the reaction of benzocyclobutadiene with butadiyne.

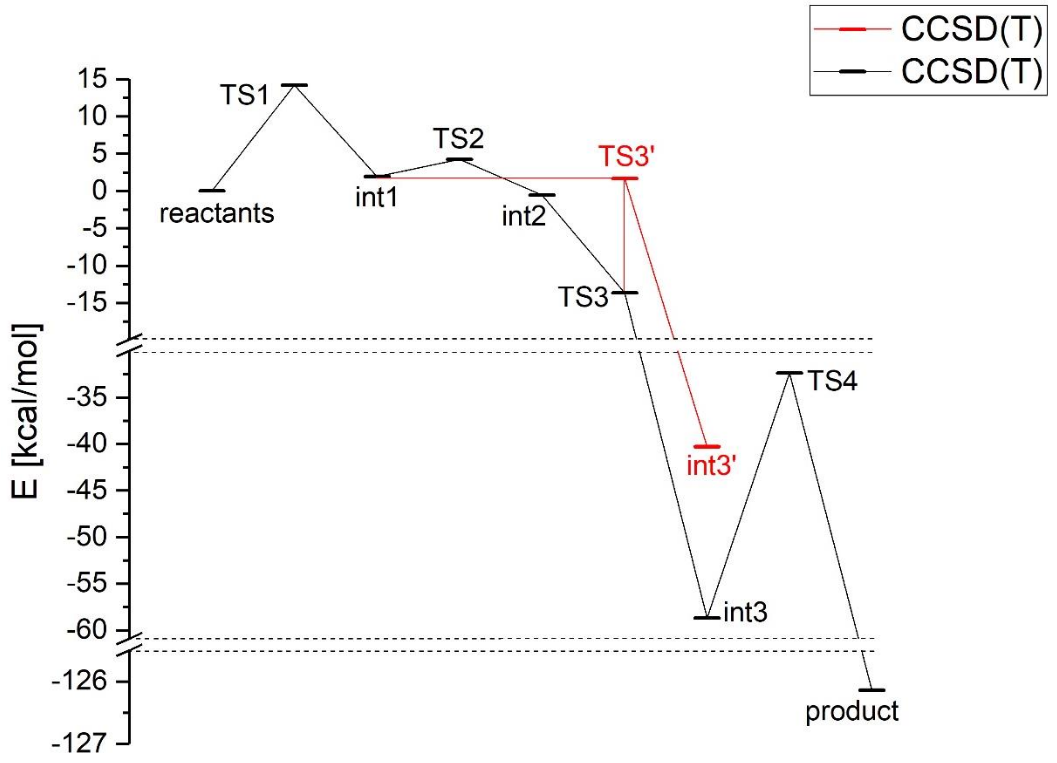

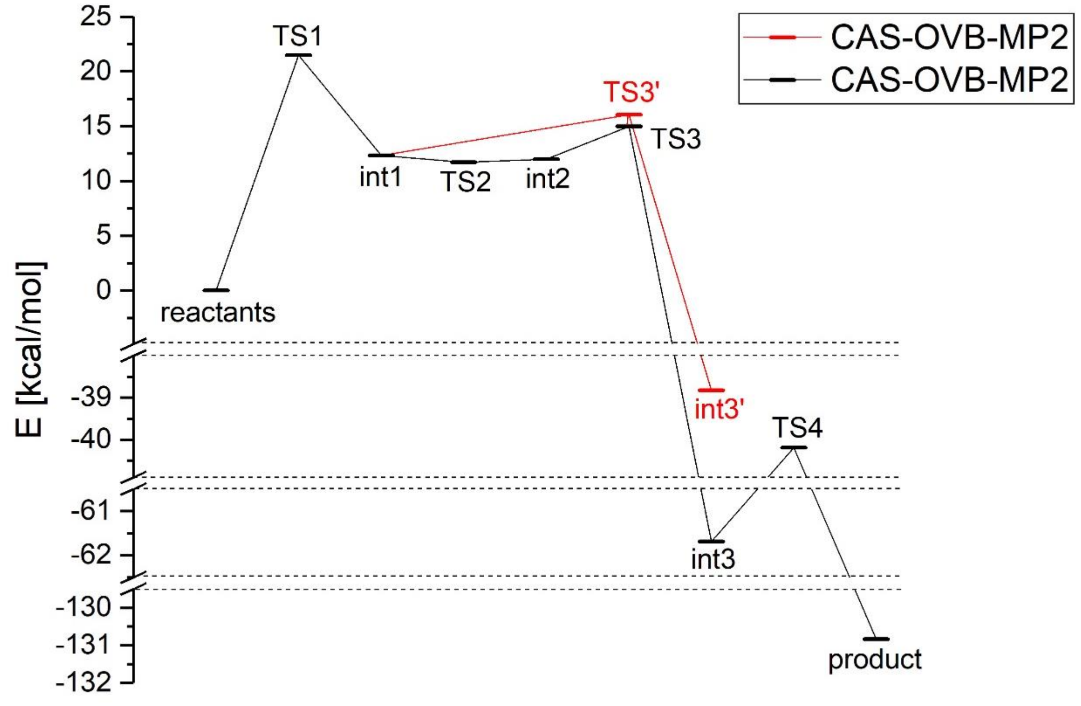

Figure S20. Top: Computed energies for the reaction mechanism given in Figure S19 at the CCSD(T)/aug-cc-pVDZ level of theory. For the labelling of the various stationary points, see Figure S19. Bottom: Computed energies for the reaction mechanism given in Figure S19 at the

CAS-OVB-MP2(8,8)/aug-cc-pVDZ level of theory. For the labelling of the various stationary points, see Figure S19.

Table S2: Single point energies [kcal/mol] of the various stationary points along the reaction path depicted in Figure S19

	MP2	SCS-MP2	CC2	SCS-CC2	CCSD
**reactants**	0.0	0.0	0.0	0.0	0.0
**TS1**	6.99	14.58	4.59	12.42	22.93
**int1**	5.45	13.91	-7.54	1.14	22.41
**TS2**	5.14	13.71	-5.69	3.04	24.16
**int2**	10.43	18.63	-6.89	1.71	23.76
**TS3**	26.19	33.49	-0.23	7.58	30.39
**TS3’**	14.65	22.53	-0.82	7.26	28.53
**int3**	-62.85	-58.04	-62.56	-57.52	-57.84
**int3’**	-40.73	-34.78	-42.56	-35.86	-35.65
**TS4**	-38.02	-29.79	-39.10	-30.62	-27.09
**product**	-132.97	-126.24	-131.70	-124.97	-124.92

Table S3. Single point energies [kcal/mol] of the various stationary points along the reaction path depicted in Figure S19. All energies are given with respect to the **reactants** benzocyclobutadiene and butadiyne. The geometries were determined employing UB3LYP-D3/6-311++G(d,p). For the labelling of the various stationary points, see Figure S19. In addition, we obtained single-point energies using M06-2X-D3, CCSD(T), and CAS-OVB-MP2(8,8) in combination with the aug-cc-pVDZ basis set.

	M06-2X-D3	CCSD(T)	D1-amplitude	CAS-OVB-MP2(8,8)
**reactants**	0.0	0.0	0.033+0.0281	0.0
**TS1**	20.25	14.21	0.072	21.51
**int1**	22.19	1.95	0.262	12.32
**TS2**	23.80	4.26	0.217	11.72
**int2**	23.84	-0.56	0.345	12.00
**TS3**	31.86	-13.68	0.570	15.00
**TS3’**	29.24	1.70	0.369	16.04
**int3**	-57.58	-58.68	0.032	-61.69
**int3’**	-34.13	-40.30	0.039	-38.83
**TS4**	-27.31	-32.39	0.079	-40.19
**product**	-126.60	-126.14	0.030	-130.83

The first step of the overall reaction represents a formal intramolecular [4+2]-cycloaddition within a 1,11-bis(*p*-tolyl)undeca-1,3,8,10-tetrayne **3**. In this reaction, as we have described in the main text, two alkyne units of one butadiyne react with one alkyne unit of the other butadiyne. Step 2, which connects the intermediate **Me-BZ-Me** from Figure 10 (main text) with $\mathbf{I}_{\mathbf{a/b}}$, is a formal [2+2]-cyclization of the *o*-benzyne moiety formed in Step 1 and one of the alkyne moieties of an additional molecule of **3**. The corresponding model reaction between *o*-benzyne and butadiyne has already been computed by Johnson *et al.* in 2010[14] employing the CCSD(T)/6-311+G(d,p)//B3LYP/6-311+G(d,p) +ZPVE level of theory. The

data are summarized in Figure S17. The calculations predicted that the biradical intermediate **Johnson_2** is formed with a reaction barrier of only ca. 11 kcal/mol. This biradicaloid intermediate is already ca. -20 kcal/mol more stable than the reactants (**Johnson_1**). According to Johnson *et al.*, this intermediate can either form a four- (**Johnson_3**) or a six-membered ring (**Johnson_4**).

The six-membered ring is thermodynamically favored (-96 kcal/mol vs. -66 kcal/mol), but the formation of the four-membered ring is kinetically facilitated because its reaction barrier of about 2 kcal/mol is slightly lower than the reaction barrier (ca. 5 kcal/mol) leading to **Johnson_4**. Both barriers are given with respect to intermediate **Johnson_2**, *i.e.* assuming that the reaction energy from the formation of **Johnson_2** is not fully dissipated, both barriers can easily be surmounted. Hence, the formation of the benzyne derivative **Johnson_4** cannot be excluded. Its further reaction might lead to some of the various yet unknown side products.

In the next step (Step 3) of the overall reaction (Figure 10 main text), an intermediate is formed ($\mathbf{II_{a/b}}$), which contains a Dewar benzene moiety. It subsequently opens (Step 4) to $\mathbf{III_a}$ / **8**, which already contains two benzene rings. Step 3 of the mechanism from $\mathbf{I_{a/b}}$ to $\mathbf{II_{a/b}}$ (Figure 10 main text) can be formulated as a thermally allowed [4+2]-cycloaddition between an acetylene unit and the four-membered ring, if the double bonds of the four-membered ring rearrange so that they can act as a diene unit ($\mathbf{I_{a/b}}$**’**). Without this rearrangement, the transformation must be described as a thermally forbidden [2+2]-cycloaddition between both moieties. In 2017, Jones and Krebs[15] investigated the cycloaddition of methylacetylene and cyclobutadiene employing (U)B3LYP-D3 in combination with the 6-31G(d) basis set. The data are summarized in Figure S18. Their investigations predict that, for this smaller model system, both the Diels-Alder reaction and a stepwise addition via biradical intermediates represent valid pathways for the formation of a Dewar benzene derivative. The Diels-Alder reaction proceeds through a single transition state **Jones_ts1’** with an activation energy of 20 kcal/mol. The biradical pathway starts with a π-bond addition of the methylacetylene to the cyclobutadiene also possessing a barrier of ca. 20 kcal/mol. The resulting biradical intermediate **Jones_int1** can then be converted into **Jones_int2** by a rotation around the C^3-C^4 double-bond with a rotation barrier of less than 2 kcal/mol. The follow-up rotation of the dihedral C^1-C^2-C^3-C^4 angle with a barrier below 1 kcal/mol then leads to the ring closure resulting in the Dewar benzene derivative **Jones_int3**. According to their computations, the Dewar benzene moiety can then open to provide **Jones_p1** with a barrier of ca. 30 kcal/mol. The total reaction energy of Step 3 and Step 4 amounts to -124.7 kcal/mol, *i.e.* the overall

reaction is strongly exothermic. Two key differences exist between the model system investigated by Jones and Krebs[15] and our present system. First, our system contains a benzocyclobutadiene unit instead of a cyclobutadiene. Going from cyclobutadiene to benzocyclobutadiene, one double bond of the cyclobutadiene subunit is integrated into the benzene ring. Consequently, an electronic structure as indicated in **$I_{a/b}$'** is highly unfavorable and, as such, the four-membered ring can no longer act as a diene, but only as a double bond. Hence, the concerted cycloaddition via the [4+2] pathway becomes highly unfavorable. According to our CCSD(T)/aug-cc-pVDZ calculations, the activation barrier increases from ca. 20 kcal/mol, which was predicted for cyclobutadiene,[15] to more than 60 kcal/mol. A second difference in our system is that the cyclobutadiene subunit reacts with a butadiyne instead of methylacetylene. To investigate effects resulting from this difference, we recomputed the reaction paths for our system. The details of the mechanisms are depicted in Figure S19, and the computed relative energies are summarized in Table S2 and S3. Figure S20 gives single-point relative energies of the various stationary points using CCSD(T) (Figure S20 (top)) or CAS-OVB-MP2(8,8) (Figure S20 (bottom)), both in combination with the aug-cc-pVDZ basis set. To determine the geometries of the stationary points we used UB3LYP-D3/6-311++G(d,p). Going from one to two acetylene units, besides the four-membered ring (**int3**), a six-membered ring (**int3'**) can also be formed. Both paths proceed through **int1**. According to our CAS-OVB-MP2(8,8)/aug-cc-pVDZ calculations, C-C bond formation between the atoms C^2 and C^3 of the reactants leading to **int1** possesses a barrier of ca. 22 kcal/mol (**TS1**). From this intermediate, the six-membered ring can be formed directly via **TS3'**. The formation of the four-membered ring proceeds along a similar reaction path as described by Jones and Krebs for the biradical reaction of the mono-acetylene with cyclobutadiene. It includes the rotation around the C^3-C^4 bond leading, via **TS2,** to **int2** followed by a ring closure to **int3** via **TS3**. M06-2X-D3-calculations predict that the six-membered ring formation of the benzyne derivative **int3'** is kinetically favored because the barrier connected with **TS3'** (29 kcal/mol) is slightly lower than the combined barriers for **TS2** (ca. 24 kcal/mol) and **TS3** (ca. 32 kcal/mol) leading from **int1** to the four-membered ring.

It is important to note that M06-2X-D3 predicts that both possible second steps (**TS2/TS3** and **TS3'**) possess a higher barrier than the formation of **int1**. MP2, SCS-MP2, CC2, SCS-CC2 and CCSD calculations lead to a similar conclusion (Table S2). In contrast, CCSD(T) and CAS-OVB-MP2(8,8) calculations indicate that the pathway leading to the Dewar benzene

derivative **int3** via **int2** is the energetically favored one. CCSD(T) predicts a barrier of only ca. 2 kcal/mol for the conversion of **int1** to **int2** which is ca. -2 kcal/mol lower in energy than **int1**. Additionally, the barrier **TS3** is lower in energy than **TS3'**. CCSD(T) predicts that **TS3** is ca. 13 kcal/mol more stable than **int2**, so that the reaction can directly form the four-membered ring from **int2** without any further barrier. It is important to note that CCSD(T) computes the barriers for the second step (**TS2**/**TS3** and **TS3'**) to be considerably lower than the barrier for the first step (**TS1**). The significant difference between CCSD(T) and the other single-reference approaches indicates that the electronic structures of the intermediates are very difficult to compute. This is also indicated by the D1-amplitudes[16] (Table S3). They are a measure for the multi-reference character of a wave function. For values larger than 0.05, experience shows that multi-reference effects become so strong that all single-reference methods should be handled with care. To obtain more reliable insight, we employed the CAS-OVB-MP2 approach. The CAS-OVB-MP2(8,8) results predict no barrier between **int1** and **int2**, and place **int2** slightly lower in energy (-1.1 kcal/mol) than **int1**. CAS-OVB-MP2(8,8) computes the barrier between **int2** and **int3** to be ca. 3 kcal/mol (**TS3**). The barrier to form the six-membered ring is slightly higher (**TS3'** 4 kcal/mol); *i.e.*, CAS-OVB-MP2(8,8) also favors the formation of the four-membered ring, which is in line with our experimental findings.

According to our CAS-OVB-MP2(8,8) computations, the conversion of **int3** to the naphthalene **product** via **TS4** should possess an activation barrier of ca. 24 kcal/mol. The final **product** is predicted to lie -92.9 kcal/mol below **TS4**. The overall reaction energy is computed to be -131 kcal/mol. The barrier is, therefore, ca. 7 kcal/mol below the results of the ring opening in the Dewar benzene derivative **Jones_int3** investigated by Jones and Krebs.[15] Considering the various very exothermic steps along the overall reaction path (Figure 10 main text) up to $\mathbf{II_{a/b}}$, the system should contain sufficient energy to surmount this barrier quite quickly. This underpins the validity of the proposed mechanism up to $\mathbf{III_{a}}$ **/ 8**. The computed data supports the formation of a Dewar benzene derivative, but the differences in the barrier heights are so small that the formation of the benzyne derivative (**int3'**) as a reactive side product cannot be excluded. It may be responsible for some of the additional as of yet unidentified side products.

Table S4. Relative energies (in kcal/mol) of one reaction path of the reaction of *o*-benzyne and 1,11-bis(*p*-tolyl)undeca-1,3,8,10-tetrayne leading to a strand break of 1,11-bis(*p*-tolyl)undeca-1,3,8,10-tetrayne. The CAS-OVB-MP2(8,8) computations were performed with the aug-cc-pVDZ basis set. The geometries were obtained from UB3LYP-D3/6-311++G(d,p) optimizations (see text).

structure	C_2-C_3 [Å]	C_1-H [Å]	B3LYP-D3	S^2	CAS-OVB-MP2(8,8)
**SB 1**	2.51	2.04	0.0	0.0	0.0
**SB 2**	2.11	2.04	2.4	0.0	19.3
**SB 3**	1.51	2.04	-25.3	1.0214	-9.5
**SB 3’**[1]	1.48	2.78	-28.2	1.0224	-12.2
**SB 4**	2.11	1.74	6.9	0.0	25.9
**SB 5**	1.51	1.44	-18.7	1.0104	-3.6
**SB 6**	1.51	1.34	-25.8	0.8976	-10.0
**SB 6”**	1.51	1.34	-22.4	0.1245	-9.7
**SB 7**	1.51	1.24	-35.1	0.8982	-9.5
**SB 7”**	1.51	1.24	-31.7	0.1206	-19.2
**SB 8**	1.51	1.14	-41.4	0.899	-25.4
**SB 8”**	1.51	1.14	-38.0	0.1173	-25.3
**SB 9**	1.51	1.04	-41.9	0.8999	-25.2
**SB 9”**	1.51	1.04	-38.5	0.1137	-25.2
**SB 10**	1.42	-	-71.7	0.0	-59.9
**SB 11**	2.51	1.34	-66.6	-	-

[1] Obtained by a full optimization starting from **SB 3**.

[2] **SB X”** gives the barrier of the scan with increasing C_3-C_4 distances starting at SB X and ending at **SB 10**.

The PES shows a barrier of 2.4 kcal/mol at **SB 2** (19.3 kcal/mol for the CAS-OVB-MP2(8,8)/aug-cc-pVDZ single-point calculation) between the initial structure **SB 1** (see Figure 11 and Figure 12 in the main text) and the biradical structure **SB 3**, *i.e.* in a first step,only the C^2-C^3 bond is formed (see Figure 12 main text). In comparision the concerted reaction path via **SB 4** has to overcome a barrier of 6.9 kcal/mol (25.9 kcal/mol CAS-OVB-MP2(8,8)/aug-cc-pVDZ). A full optimization starting from **SB 3** leads to structure **SB 3’** which, using CAS-OVB-MP2(8,8), is lower in energy than the structure **SB 3** (see Table S4). Structure **SB 3’** represents a complete biradical as indicated in Figure 11 (main text). Using the most accurate CAS-OVB-MP2(8,8) approach, it is about -12 kcal/mol more stable than the starting structure **SB 1**, *i.e.* the biradicalic intermediate represents a shallow local minimum on the PES. The CAS-OVB-MP2(8,8)/aug-cc-pVDZ single-point calculations predict that, starting from this biradical intermediate **SB 3’**, a barrier of about 8-9 kcal/mol (structure **SB 5**) has to be overcome to get to the next minimum **SB 9**, which is about 25 kcal/mol lower in energy than the starting structure **SB 1**. In structure **SB 9**, the hydrogen is already attached to the C^1 center, but the C^3-C^4 bond is not yet broken, *i.e.* in the UB3LYP/6-311++G(d,p) calculations, the C^3-C^4 bond does not break spontaneously if the hydrogen is shifted to C^1. To estimate the barrier between structure **SB 9** and the final product **SB 10**, *i.e.* to estimate the height of barrier which has to be overcome to break the C^3-C^4 bond, we scanned the potential energy curve starting from structure **SB 9** and stretched the C^3-C^4 bond within the UB3LYP/6-311++G(d,p) level of theory. Structure **SB 9”**, which represents the top of the barrier on the reaction path from **SB 9** to the final product **SB 10**, is less than 1 kcal/mol higher in energy than **SB 9** according to CAS-OVB-MP2(8,8) single-point calculations, *i.e.* the final C^3-C^4 bond cleavage has no further real barrier. Similar scans were also started from the structures **SB 6** – **SB 8**. Starting from **SB 6**, the barrier is also less than 0.5 kcal/mol (Table S4, structure **SB 6”**: -9.7 kcal/mol). The scan starting from structure **SB 7** already indicated no further barrier, *i.e.* from structure **SB 7**, the system can relax barrier-free to structure **SB 9**, but also directly to **SB 10**. In summary, our CAS-OVB-MP2(8,8)

calculations support our proposal that the final product **SB 10** can be formed via the mechanisms depicted in Figure 11 (main text). According to our calculations, the strand cleavage is a stepwise reaction that proceeds through intermediate **SB 3'** that is formed from the starting structure **SB 1** with a reaction barrier of ca. 19 kcal/mol.

However, intermediate **SB 3'** represents only a shallow minimum on the PES. It can easily rearrange to the identified product **SB 10** (barrier height ~ 8 – 10 kcal/mol), but assuming high reactivity and a non-vanishing lifetime, further reactions of intermediate **SB 3'** with other compounds may also lead to other, perhaps yet unidentified side products.

Optimized geometry of reactants (benzocyclobutadiene)

6	-0.002147	-0.002256	0.003969	6	-0.486993	-0.511695	-1.159164
6	-0.003412	-0.003585	1.528474	1	2.249989	2.364117	-1.081565
6	0.927137	0.974164	1.562251	1	1.386314	1.456633	-3.257657
6	0.978478	1.028110	0.039530	1	-0.295967	-0.310980	-3.318649
6	1.502712	1.578935	-1.087188	1	-1.233525	-1.296094	-1.207702
6	1.003168	1.054052	-2.326700	1	-0.534243	-0.561342	2.287589
6	0.054597	0.057366	-2.361058	1	1.431054	1.503642	2.359019

Optimized geometry of reactants (butadiyne)

6	2.394696	0.054438	11.224488	6	0.623235	1.851943	11.720545
6	1.560590	0.895969	11.455536	6	-0.200980	2.701351	11.958132
1	3.131083	-0.684762	11.022887	1	-0.924447	3.450519	12.169506

Optimized geometry of TS1

6	0.000000	0.000000	0.000000	6	3.113560	0.377180	2.252040
6	1.550630	0.000000	0.000000	6	1.487830	-1.330190	-0.465490
6	1.990400	0.000000	1.848410	6	0.021540	-1.367680	-0.373960

6	-1.138620	-2.091700	-0.552330	1	-1.178980	1.749170	0.469570
6	-2.347300	-1.382410	-0.330310	1	2.216580	0.784250	-0.334510
6	-2.359460	-0.043660	0.028680	1	2.248450	-2.036720	-0.767320
6	-1.152150	0.699740	0.199630	1	1.047910	-0.326130	2.261320
1	-1.151810	-3.128690	-0.866800	6	4.389910	0.786750	2.374460
1	-3.291120	-1.902770	-0.449890	6	5.540280	1.157970	2.515510
1	-3.309680	0.451810	0.192660	1	6.543990	1.482310	2.641950

Optimized geometry of int1

6	0.000000	0.000000	0.000000	6	-2.842220	0.920590	1.092340
6	1.288630	0.000000	0.000000	1	-1.549260	1.385710	1.478950
6	2.579220	0.000000	0.316380	1	3.780020	0.004030	0.565210
1	-0.620100	-0.076270	-0.896210	1	-4.584730	2.242040	1.022700
6	-0.920760	0.055900	1.285110	1	-6.075170	0.360950	0.356320
6	-2.368790	-0.409500	0.940950	1	-5.233050	-1.915990	0.093980
6	-3.178240	-1.458540	0.603260	1	-2.825090	-2.478190	0.499280
6	-4.542430	-1.133050	0.385890	1	-0.397000	-0.411810	2.117220
6	-5.020910	0.173340	0.528460	6	-1.131830	2.344060	1.756220
6	-4.188340	1.242470	0.889660	1	4.821120	-0.003220	0.775230

Optimized geometry of TS2

6	0.000000	0.000000	0.000000	6	-5.008480	0.592670	-0.344370
6	1.289310	0.000000	0.000000	6	-4.120680	1.186460	-1.251370
6	2.577890	0.000000	0.315890	6	-2.793450	1.295240	-0.818440
1	-0.618480	-0.889100	-0.158950	6	-1.479560	1.761730	-1.112000
6	-0.924710	1.261560	0.174450	6	3.770190	0.005390	0.605380
6	-2.394200	0.830260	0.463070	1	-4.462070	1.554240	-2.211640
6	-3.259980	0.263660	1.357910	1	-6.050340	0.479520	-0.623920
6	-4.602620	0.142200	0.917050	1	-5.334790	-0.318650	1.570250

1	-2.963350	-0.079950	2.342420	1	-1.006640	2.236560	-1.960650
1	-0.442240	1.966480	0.850030	1	4.797690	-0.008670	0.873780

Optimized geometry of int2

6	0.000000	0.000000	0.000000	6	-2.801370	-1.067560	-1.076370
6	1.290700	0.000000	0.000000	6	-1.491240	-1.441030	-1.479440
6	2.580360	0.000000	0.316150	6	3.782370	-0.010730	0.561530
1	-0.601830	0.077820	0.914410	1	-4.463180	-2.495290	-1.046160
6	-0.939040	-0.071510	-1.242130	1	-6.053760	-0.739120	-0.284850
6	-2.406120	0.281460	-0.864550	1	-5.346750	1.570150	0.076390
6	-3.275360	1.262480	-0.467680	1	-2.982390	2.295070	-0.315220
6	-4.612260	0.845750	-0.256650	1	-0.483870	0.452470	-2.081300
6	-5.014150	-0.482430	-0.457210	1	-1.011590	-2.368160	-1.762140
6	-4.126910	-1.479830	-0.874240	1	4.816660	-0.016510	0.802620

Optimized geometry of TS3

6	2.738737	-0.242670	0.007517	6	3.970943	-0.036354	-0.436892
6	1.605137	-0.737981	0.371662	6	5.106475	0.207772	-0.835624
6	0.366287	-0.090684	1.083310	1	1.462491	-1.824263	0.355206
6	-0.042298	1.316132	0.790989	1	-2.715869	2.397769	-0.544392
6	-1.282011	0.931302	0.219767	1	-4.297026	0.600970	-1.237007
6	-1.012786	-0.446136	0.438628	1	-3.800656	-1.761022	-0.863744
6	-1.875908	-1.441668	0.084014	1	-1.679678	-2.494973	0.249950
1	0.496147	2.251989	0.858825	1	0.479100	-0.346304	2.142316
6	-3.359904	0.334919	-0.760276	6	-3.080485	-1.017000	-0.543229
6	-2.473738	1.354369	-0.382094	1	6.092785	0.383822	-1.187797

Optimized geometry of TS3'

6	2.707217	-0.666821	-0.226415	6	3.786691	0.072123	-0.450530
6	1.562349	-1.128260	0.134016	6	4.791059	0.740315	-0.674990
6	0.415353	-0.446439	1.013718	1	1.277079	-2.167566	-0.036614
6	0.165800	1.012753	0.976449	1	-2.367317	2.588876	-0.128889
6	-1.109642	0.868530	0.356246	1	-4.159262	1.113478	-1.041139
6	-1.003166	-0.546967	0.360308	1	-3.945661	-1.318794	-1.032427
6	-1.986370	-1.371437	-0.100115	1	-1.920279	-2.453529	-0.088116
1	0.777292	1.859457	1.258457	1	0.544912	-0.896876	2.003205
6	-3.256217	0.670338	-0.635802	6	-3.139319	-0.719223	-0.625752
6	-2.247161	1.512179	-0.135312	1	5.673675	1.292401	-0.885399

Optimized geometry of int3

6	0.613447	0.092061	0.510895	6	0.367868	0.372752	-0.848396
6	-0.135927	-0.343925	1.543045	6	0.204171	0.617345	-2.018678
6	0.982759	-0.239065	2.571150	1	-1.183777	-0.604089	1.631523
6	1.881301	0.273006	1.363096	1	3.135613	3.229354	1.264239
6	1.983495	1.595544	2.122302	1	2.607637	4.710548	3.199634
6	1.197778	1.149621	3.184758	1	1.228513	3.928065	5.064311
6	0.894588	1.953421	4.270077	1	0.293472	1.617612	5.108096
6	2.788997	-0.244324	1.054322	1	1.250329	-1.120645	3.153776
6	2.216699	3.699172	3.174099	6	1.428016	3.251891	4.240012
1	2.516011	2.872502	2.079489	1	0.047549	0.832501	-3.047372

Optimized geometry of int3'

6	0.687212	-1.543490	0.114120	6	0.366802	0.584852	1.123837
6	0.979138	-0.512104	0.886582	1	1.206872	-2.487797	0.004219

6	-0.631662	-1.286030	-0.657521	6	-0.782118	1.069957	0.685911
6	-0.465929	-0.784077	-2.093732	1	-1.718807	2.355897	-2.667448
6	0.028671	-1.129428	-3.340510	1	-0.822659	1.779002	-4.918482
6	-0.117342	-0.166038	-4.350117	1	0.256351	-0.378021	-5.345930
6	-0.733742	1.066229	-4.105920	1	0.508227	-2.080183	-3.545684
6	-1.239310	1.398695	-2.839957	1	-1.290144	-2.156461	-0.555421
6	-1.082844	0.441078	-1.851881	1	-2.455033	-0.004379	-0.186800
6	-1.385050	0.129344	-0.386024	1	-1.300563	1.975947	0.975723

Optimized geometry of TS4

6	1.910668	-0.275468	0.332969	1	2.677092	0.715566	-0.310086
6	1.621721	-1.583557	-0.137939	1	3.373353	1.556745	-0.827514
6	0.254612	-1.844888	0.000073	1	2.304068	-2.267977	-0.636166
6	0.804393	-0.093386	1.194770	1	-0.922704	2.461628	0.962623
6	-0.457585	0.383654	0.632421	1	-3.136037	2.261634	-0.164184
6	-0.835570	-0.841626	0.026566	6	-3.906994	0.092956	-1.043691
6	-2.107803	-0.965100	-0.537177	6	-2.448308	-1.916505	-0.933262
6	0.676934	-0.857562	1.956952	1	-0.079759	-2.869092	-0.184621
6	-2.487175	1.396243	-0.092221	6	-2.922719	0.165950	-0.595226
1	-1.242469	1.518954	0.534950	1	3.981705	2.296401	-1.287165

Optimized geometry of product (Figure 13 main text)

6	-3.165445	1.632041	-0.000469	6	-0.723311	-1.205102	0.006535
6	-4.361490	0.953840	-0.014698	6	0.488743	-0.535808	0.020891
6	-4.380565	-0.461119	-0.022267	6	0.497819	0.890851	0.028359
6	-3.203811	-1.170591	-0.015461	6	1.722196	-1.253914	0.028068
6	-1.951132	-0.500512	-0.000761	6	2.770877	-1.848159	0.034182
6	-1.932964	0.930395	0.006882	1	-0.735460	-2.289133	0.000737
6	-0.677686	1.595019	0.021563	1	1.450075	1.406782	0.039516

1	-0.659662	2.679835	0.027382
1	-3.150231	2.716997	0.005333
1	-5.296832	1.502021	-0.020193
1	-5.330219	-0.983919	-0.033492
1	-3.216699	-2.255451	-0.021244
1	3.693059	-2.375814	0.039518

Optimized geometry of SB 1

6	-4.896211	1.044402	0.578260
6	-3.673447	0.360196	0.193992
1	-4.676660	2.026504	1.009000
1	-5.449436	0.466851	1.325448
1	-5.551926	1.194023	-0.285148
6	-2.652198	-0.210835	-0.120643
6	-1.506134	-0.852125	-0.474295
6	-0.435704	-1.346473	-0.775130
1	0.710079	-2.167009	-1.173933
1	1.633524	-2.561288	-0.001432
1	0.321381	-3.065948	-1.668363
1	1.293475	-1.619168	-1.921843
6	0.936516	-3.388723	1.077350
6	2.023573	-1.618683	0.419874
6	2.484939	-3.110372	-0.416712
6	0.104287	-2.833923	1.519195
6	1.632318	-3.645829	1.880342
6	0.535138	-4.322771	0.669585
1	1.270262	2.944741	-0.321978
1	1.952427	0.420447	0.392507
6	2.513761	2.692929	0.284102
1	2.909215	1.398276	0.668104
6	0.864465	0.711142	-0.162039
6	0.345800	1.912181	-0.580029
1	-0.618665	2.077723	-1.042329
1	1.011513	3.960769	-0.602587
1	3.186722	3.525573	0.464920
1	3.869193	1.218090	1.139602

Optimized geometry of SB 2

6	-4.927665	1.194351	0.595779
6	-3.686890	0.552659	0.202205
1	-4.732937	2.172153	1.048570
1	-5.466301	0.587109	1.330047
1	-5.584517	1.347034	-0.266212
6	-2.643824	0.019829	-0.118910
6	-1.491213	-0.588083	-0.477530
6	-0.359530	-0.994994	-0.750390
1	0.613604	-2.016452	-1.205978
1	1.415388	-2.629181	-0.043383
1	0.051951	-2.791057	-1.739403
1	1.302247	-1.556482	-1.920735
6	0.562658	-3.458377	0.915883
6	1.881162	-1.779223	0.490050
6	2.224321	-3.233276	-0.466324
6	-0.227546	-2.847902	1.361649

6	1.172282	-3.861796	1.728624	6	0.900269	0.586471	-0.147089
6	0.085181	-4.300968	0.404380	6	0.644694	1.800625	-0.752473
1	1.639075	2.748481	-0.461693	1	-0.202975	2.013523	-1.392400
1	1.854627	0.256241	0.631638	1	1.562215	3.742666	-0.889493
6	2.729531	2.432742	0.365056	1	3.471126	3.197770	0.575950
1	2.874070	1.158450	0.938321	1	3.715023	0.933879	1.586688

Optimized geometry of SB 3

6	-5.238200	1.103716	0.250565	6	1.339276	-3.616324	-0.574794
6	-3.874880	0.657516	0.037932	6	0.165622	-2.042376	1.801739
1	-5.292499	2.193567	0.359939	6	1.134792	-3.516493	1.922810
1	-5.659877	0.657791	1.157892	6	-0.480980	-3.572235	1.203175
1	-5.883731	0.822431	-0.589220	1	1.909687	2.547001	0.318308
6	-2.709710	0.285334	-0.143482	1	2.248108	-0.072943	-0.307742
6	-1.473000	-0.112687	-0.335079	6	3.202419	2.016827	0.258399
6	-0.234073	-0.515506	-0.525912	1	3.382206	0.664312	-0.061609
1	0.083792	-1.920404	-1.025574	6	0.947431	0.386131	-0.259080
1	1.031090	-2.685593	-0.086797	6	0.798540	1.750447	0.065405
1	-0.849397	-2.472203	-1.162819	1	-0.202434	2.168402	0.114129
1	0.558650	-1.826047	-2.010075	1	1.770083	3.593400	0.565367
6	0.430456	-2.971178	1.289011	1	4.063646	2.645513	0.458393
6	1.938533	-2.063381	0.018121	1	4.375126	0.230116	-0.110742

Optimized geometry of SB 3'

6	-5.192710	0.652239	0.265740	6	-2.567072	0.221455	-0.099638
6	-3.776321	0.412314	0.067443	6	-1.269756	0.066098	-0.256883
1	-5.386511	1.692218	0.554970	6	-0.039393	-0.345152	-0.502878
1	-5.594947	0.007879	1.055103	1	0.220697	-1.772291	-0.970934
1	-5.760100	0.451277	-0.649687	1	0.771155	-2.684248	0.144303

1	-0.707224	-2.196181	-1.363590	1	2.420613	0.154115	-0.671205
1	0.941789	-1.733313	-1.795883	6	3.434659	2.169628	0.029258
6	-0.221252	-2.912034	1.286997	1	3.569421	0.893569	-0.535096
6	1.699453	-2.255007	0.534938	6	1.141723	0.529730	-0.310016
6	1.041623	-3.645226	-0.307746	6	1.044922	1.819986	0.257538
6	-0.488001	-1.969799	1.772543	1	0.066012	2.174386	0.567477
6	0.200077	-3.575576	2.047078	1	2.062776	3.606234	0.857400
6	-1.146833	-3.366319	0.918766	1	4.308107	2.799459	0.160192
1	2.169337	2.620116	0.419650	1	4.538432	0.519579	-0.847580

Optimized geometry of SB 4

6	-5.038945	0.965083	0.584092	6	2.463889	-2.979060	-0.509888
6	-3.777398	0.357496	0.199687	6	0.162496	-2.686551	1.525313
1	-4.879852	1.959353	1.014317	6	1.708218	-3.486124	1.835157
1	-5.555200	0.355500	1.332465	6	0.558660	-4.194497	0.692915
1	-5.703232	1.074319	-0.278911	1	1.437782	2.856062	-0.294975
6	-2.719186	-0.147030	-0.115206	1	1.916241	0.273288	0.386945
6	-1.544729	-0.721936	-0.467734	6	2.639957	2.506364	0.343415
6	-0.409628	-1.100574	-0.745569	1	2.925532	1.178931	0.706767
1	0.643107	-2.046002	-1.186571	6	0.855933	0.630979	-0.212488
1	1.617344	-2.445429	-0.067472	6	0.460234	1.892837	-0.600596
1	0.135846	-2.928843	-1.591235	1	-0.473551	2.136027	-1.093424
1	1.203962	-1.588036	-2.007675	1	1.260277	3.892346	-0.564065
6	0.975860	-3.249763	1.058745	1	3.361911	3.285000	0.570960
6	2.012419	-1.463625	0.328538	1	3.851530	0.922358	1.211291

Optimized geometry of SB 5

6	-5.398375	0.909694	0.311694	1	-5.515724	1.932456	0.689935
6	-4.001453	0.576847	0.107994	1	-5.864542	0.232769	1.035983

1	-5.962779	0.835636	-0.624344	6	1.600373	-3.419290	1.619749
6	-2.806347	0.313452	-0.058953	6	-0.061937	-3.756698	1.115016
6	-1.521413	0.074500	-0.220585	1	2.078834	2.541578	0.146680
6	-0.311444	-0.385983	-0.451859	1	2.161659	-0.190213	-0.109615
1	-0.071641	-1.798667	-1.009508	6	3.303125	1.872966	0.250786
1	1.047824	-2.527196	-0.279477	1	3.348084	0.480441	0.124058
1	-1.007740	-2.363558	-0.966746	6	0.930875	0.439099	-0.215254
1	0.197705	-1.690771	-2.067015	6	0.901166	1.838294	-0.083988
6	0.724079	-2.991208	1.126281	1	-0.047297	2.359378	-0.168679
6	1.907774	-1.605987	-0.189130	1	2.046933	3.621213	0.243081
6	1.528487	-3.289400	-0.896286	1	4.215449	2.432033	0.430947
6	0.362683	-2.159248	1.737734	1	4.290779	-0.050161	0.210230

Optimized geometry of SB 6

6	5.085135	-1.595463	-0.003101	6	-0.944835	2.871454	0.390371
6	3.700320	-1.174831	-0.104969	6	1.354628	2.100697	1.808810
1	5.164827	-2.596755	0.436138	6	0.817488	3.785325	1.850302
1	5.665909	-0.909587	0.623165	6	2.081449	3.267374	0.714145
1	5.558594	-1.628970	-0.990230	1	-2.734204	-1.936672	0.728867
6	2.524377	-0.823542	-0.190631	1	-2.370431	0.431639	-0.648773
6	1.229825	-0.527350	-0.252964	6	-3.847550	-1.228298	0.271482
6	0.172380	0.227799	-0.453169	1	-3.659773	-0.035236	-0.423648
1	0.391730	1.705747	-0.928023	6	-1.237613	-0.257084	-0.207689
1	0.094430	2.660408	0.162206	6	-1.451675	-1.458225	0.493821
1	1.429810	1.791163	-1.255077	1	-0.591071	-2.009483	0.855798
1	-0.245569	1.871753	-1.801560	1	-2.868185	-2.863983	1.274789
6	1.132002	2.980307	1.182296	1	-4.848846	-1.600408	0.456645
6	-2.237138	1.578996	-1.329322	1	-4.510786	0.527234	-0.788209

Optimized geometry of SB 6”

6	5.287871	-1.339029	-0.009153	6	-1.137604	2.711128	0.057747
6	3.863179	-1.069739	-0.088220	6	0.377329	2.497564	2.162444
1	5.482577	-2.312278	0.455034	6	0.459058	4.107278	1.464593
1	5.807496	-0.576720	0.580973	6	1.759532	2.938590	1.147612
1	5.737771	-1.352564	-1.007410	1	-2.865594	-1.761363	0.929750
6	2.664805	-0.852436	-0.155618	1	-2.253954	0.220472	-0.901743
6	1.333739	-0.653799	-0.218645	6	-3.890924	-1.147681	0.208789
6	0.247912	-0.026348	-0.373387	1	-3.579045	-0.152422	-0.716766
1	0.502042	1.747207	-0.901139	6	-1.210556	-0.379548	-0.195704
1	-0.061050	2.566268	0.061500	6	-1.542078	-1.384885	0.729787
1	1.582860	1.757662	-0.993024	1	-0.743710	-1.860411	1.286343
1	-0.003090	1.654805	-1.858815	1	-3.097775	-2.535806	1.652279
6	0.678713	3.052632	1.262153	1	-4.922563	-1.442098	0.364818
6	-1.990038	1.169524	-1.811133	1	-4.362193	0.327355	-1.291092

Optimized geometry of SB 7

6	-5.238200	1.103716	0.250565	6	0.430456	-2.971178	1.289011
6	-3.874880	0.657516	0.037932	6	1.938533	-2.063381	0.018121
1	-5.292499	2.193567	0.359939	6	1.339276	-3.616324	-0.574794
1	-5.659877	0.657791	1.157892	6	0.165622	-2.042376	1.801739
1	-5.883731	0.822431	-0.589220	6	1.134792	-3.516493	1.922810
6	-2.709710	0.285334	-0.143482	6	-0.480980	-3.572235	1.203175
6	-1.473000	-0.112687	-0.335079	1	1.909687	2.547001	0.318308
6	-0.234073	-0.515506	-0.525912	1	2.248108	-0.072943	-0.307742
1	0.083792	-1.920404	-1.025574	6	3.202419	2.016827	0.258399
1	1.031090	-2.685593	-0.086797	1	3.382206	0.664312	-0.061609
1	-0.849397	-2.472203	-1.162819	6	0.947431	0.386131	-0.259080
1	0.558650	-1.826047	-2.010075	6	0.798540	1.750447	0.065405

1	-0.202434	2.168402	0.114129	1	4.063646	2.645513	0.458393
1	1.770083	3.593400	0.565367	1	4.375126	0.230116	-0.110742

Optimized geometry of SB 7"

6	5.276568	-1.355773	-0.014223	6	-1.106212	2.728523	0.087489
6	3.852583	-1.081737	-0.089547	6	0.455252	2.494502	2.157272
1	5.469028	-2.332454	0.443658	6	0.534824	4.104316	1.459068
1	5.799358	-0.598660	0.579773	6	1.816854	2.924318	1.110566
1	5.724991	-1.364563	-1.013210	1	-2.877827	-1.782300	0.896056
6	2.654688	-0.860771	-0.153754	1	-2.260090	0.265348	-0.870865
6	1.324254	-0.657602	-0.213739	6	-3.902333	-1.133876	0.204804
6	0.239585	-0.027981	-0.366902	1	-3.586939	-0.108283	-0.684802
1	0.500681	1.742048	-0.905572	6	-1.220646	-0.374268	-0.190087
1	-0.031901	2.569426	0.067166	6	-1.552696	-1.409733	0.700620
1	1.579282	1.740549	-1.020494	1	-0.754913	-1.912034	1.233888
1	-0.024062	1.653528	-1.853092	1	-3.111976	-2.580609	1.591548
6	0.740112	3.047981	1.250655	1	-4.935378	-1.425203	0.357136
6	-2.011930	1.174140	-1.678161	1	-4.371342	0.398770	-1.234295

Optimized geometry of SB 8

6	5.072101	-1.612468	0.011850	1	0.132332	2.661344	0.155374
6	3.689401	-1.185626	-0.092590	1	1.403500	1.770908	-1.305707
1	5.147517	-2.606492	0.468096	1	-0.289205	1.865225	-1.790840
1	5.659261	-0.919376	0.624091	6	1.209955	2.968231	1.137169
1	5.541844	-1.665966	-0.976188	6	-2.240383	1.502027	-1.098607
6	2.514668	-0.829961	-0.180034	6	-0.894145	2.892980	0.418968
6	1.222137	-0.527832	-0.243464	6	1.448272	2.083719	1.750750
6	0.162180	0.222421	-0.447765	6	0.927904	3.773298	1.819330
1	0.377577	1.696900	-0.939719	6	2.143688	3.248997	0.634696

1	-2.757253	-1.960310	0.662153	6	-1.469953	-1.493505	0.430497
1	-2.369913	0.492962	-0.582764	1	-0.614147	-2.083276	0.738978
6	-3.863519	-1.202462	0.271122	1	-2.900823	-2.917137	1.152033
1	-3.662423	0.026105	-0.352684	1	-4.868550	-1.565875	0.453119
6	-1.249495	-0.256463	-0.200183	1	-4.511491	0.624950	-0.662485

Optimized geometry of SB 8”

6	5.268492	-1.368641	-0.017177	6	-1.084238	2.741754	0.110702
6	3.845306	-1.089795	-0.089643	6	0.516192	2.492462	2.150193
1	5.457993	-2.353665	0.423778	6	0.590812	4.102654	1.451998
1	5.792210	-0.623253	0.590638	6	1.858893	2.915744	1.077030
1	5.718356	-1.361043	-1.015542	1	-2.886676	-1.800740	0.866661
6	2.647915	-0.865342	-0.151393	1	-2.263114	0.301430	-0.843529
6	1.318064	-0.658408	-0.208890	6	-3.910088	-1.124086	0.201183
6	0.233454	-0.028735	-0.360967	1	-3.591323	-0.073820	-0.657011
1	0.495822	1.738601	-0.910266	6	-1.227807	-0.370960	-0.184751
1	-0.012122	2.572209	0.070208	6	-1.560228	-1.431134	0.675461
1	1.572053	1.729120	-1.044820	1	-0.763146	-1.955033	1.188503
1	-0.045514	1.652127	-1.848674	1	-3.122794	-2.618575	1.538472
6	0.785873	3.045414	1.238622	1	-4.944279	-1.413061	0.350028
6	-2.031383	1.159594	-1.558329	1	-4.376566	0.455234	-1.185025

Optimized geometry of SB 9

6	5.063037	-1.624316	0.024334	6	1.216505	-0.529119	-0.234734
6	3.681688	-1.193546	-0.081620	6	0.155998	0.218934	-0.443578
1	5.136010	-2.612429	0.493660	1	0.369352	1.689866	-0.949109
1	5.654918	-0.925421	0.625339	1	0.160050	2.663220	0.145802
1	5.529404	-1.692990	-0.964372	1	1.386303	1.754338	-1.341200
6	2.507794	-0.835044	-0.170263	1	-0.317633	1.858609	-1.784065

6	1.262199	2.959205	1.103368	6	-3.874813	-1.184765	0.271465
6	-2.240851	1.471428	-0.956857	1	-3.663819	0.067690	-0.298256
6	-0.856613	2.915853	0.427612	6	-1.256940	-0.255446	-0.194529
6	1.506144	2.071612	1.710127	6	-1.484025	-1.516645	0.382249
6	1.003287	3.766318	1.792157	1	-0.632559	-2.132326	0.649222
6	2.187030	3.231637	0.580087	1	-2.925540	-2.952037	1.058349
1	-2.774549	-1.975458	0.611466	1	-4.882354	-1.542315	0.451056
1	-2.368801	0.531474	-0.528993	1	-4.509597	0.691418	-0.566723

Optimized geometry of SB 9”

6	5.257863	-1.384190	-0.019391	6	-1.051608	2.759873	0.138064
6	3.835397	-1.100941	-0.088622	6	0.596582	2.489169	2.138733
1	5.444962	-2.374771	0.410002	6	0.667571	4.099233	1.439497
1	5.783354	-0.647181	0.597031	6	1.915313	2.901376	1.032425
1	5.707813	-1.366038	-1.017596	1	-2.900580	-1.822775	0.826120
6	2.638488	-0.873102	-0.147580	1	-2.267647	0.347217	-0.802949
6	1.309225	-0.662261	-0.202176	6	-3.921881	-1.108379	0.197653
6	0.225581	-0.030976	-0.353616	1	-3.597954	-0.026555	-0.617480
1	0.491672	1.732258	-0.916547	6	-1.237253	-0.366818	-0.178173
1	0.016682	2.574733	0.072739	6	-1.572089	-1.458654	0.639869
1	1.564243	1.710806	-1.076147	1	-0.776512	-2.011251	1.124221
1	-0.071488	1.648803	-1.842360	1	-3.140120	-2.665584	1.465126
6	0.847876	3.040488	1.220936	1	-4.957807	-1.392998	0.342596
6	-2.051630	1.156148	-1.420946	1	-4.382957	0.532128	-1.115339

Optimized geometry of SB 10 (propene)

6	1.281430	-0.220638	-0.000007	1	2.239972	0.286139	-0.000093
6	0.133985	0.454450	0.000038	6	-1.233834	-0.162929	-0.000015
1	1.301141	-1.306782	0.000072	1	0.165252	1.542955	0.000008

1	-1.182757	-1.254620	-0.000557	1	-1.806429	0.153053	0.879164
1	-1.806672	0.153954	-0.878693				

Optimized geometry of SB 10 (ring)

6	5.572470	-0.000040	0.000299	6	-1.802543	-1.212260	0.000064
6	4.119609	-0.000075	-0.000227	6	-3.892864	-0.000096	0.000018
1	5.964209	0.992764	-0.241953	6	-3.192727	-1.207120	0.000041
1	5.964912	-0.706247	-0.738114	6	-1.087249	0.000138	0.000040
1	5.964566	-0.286393	0.981301	6	-1.802682	1.212319	0.000056
6	2.908183	0.000020	-0.000263	1	-1.256743	2.148005	0.000096
6	1.547593	0.000106	-0.000219	1	-3.732395	2.147361	0.000066
6	0.333280	0.000072	-0.000141	1	-4.976867	-0.000161	-0.000006
1	-1.256386	-2.147765	0.000198	1	-3.732091	-2.147588	0.000033
6	-3.192937	1.206940	0.000062				

References

[1] A. S. Batsanov, J. C. Collings, I. J. S. Fairlamb, J. P. Holland, J. A. K. Howard, Z. Lin, T. B. Marder, A. C. Parsons, R. M. Ward and J. Zhu, *J. Org. Chem.* **2005**, *70*, 703-706.

[2] G. Eglinton and W. McCrae, *J. Chem. Soc.* **1963**, 2295-2299.

[3] G. Sheldrick, *Acta Crystallogr. A* **2015**, *71*, 3-8.

[4] G. Sheldrick, *Acta Crystallogr. A* **2008**, *64*, 112-122.

[5] C. B. Hübschle, G. M. Sheldrick and B. Dittrich, *J. Appl. Crystallogr.* **2011**, *44*, 1281-1284.

[6] C. Müller, R. J. Lachicotte and W. D. Jones, *Organometallics* **2002**, *21*, 1118-1123.

[7] a) M. J. Frisch, G. W. Trucks, H. B. Schlegel, G. E. Scuseria, M. A. Robb, J. R. Cheeseman, J. Montgomery, J. A., T. Vreven, K. N. Kudin, J. C. Burant, J. M. Millam, S. S. Iyengar, J. Tomasi, V. Barone, B. Mennucci, M. Cossi, G. Scalmani, N. Rega, G. A. Petersson, H. Nakatsuji, M. Hada, M. Ehara, K. Toyota, R. Fukuda, J. Hasegawa, M. Ishida, T. Nakajima, Y. Honda, O. Kitao, H. Nakai, M. Klene, X. Li, J. E. Knox, H. P. Hratchian, J. B. Cross, V. Bakken, C. Adamo, J. Jaramillo, R. Gomperts, R. E. Stratmann, O. Yazyev, A. J. Austin, R. Cammi, C. Pomelli, J. W. Ochterski, P. Y. Ayala, K. Morokuma, G. A. Voth, P. Salvador, J. J. Dannenberg, V. G. Zakrzewski, S. Dapprich, A. D. Daniels, M. C. Strain, O. Farkas, D. K. Malick, A. D. Rabuck, K. Raghavachari, J. B. Foresman, J. V. Ortiz, Q. Cui, A. G. Baboul, S. Clifford, J. Cioslowski, B. B. Stefanov, G. Liu, A. Liashenko, P. Piskorz, I. Komaromi, R. L. Martin, D. J. Fox, T. Keith, M. A. Al-Laham, C. Y. Peng, A. Nanayakkara, M. Challacombe, P. M. W. Gill, B. Johnson, W. Chen, M. W. Wong, C. Gonzalez and J. A. Pople in *Gaussian 03 Rev. E.01*, *Vol.* Gaussian, Inc., Wallingford, CT, **2004**; b) M. J. Frisch, G. W. Trucks, H. B. Schlegel, G. E. Scuseria, M. A. Robb, J. R. Cheeseman, G. Scalmani, V. Barone, G. A. Petersson, H. Nakatsuji, X. Li, M. Caricato, A. V. Marenich, J. Bloino, B. G. Janesko, R. Gomperts, B. Mennucci, H. P. Hratchian, J. V. Ortiz, A. F. Izmaylov, J. L. Sonnenberg, Williams, F. Ding, F. Lipparini, F. Egidi, J. Goings, B. Peng, A. Petrone, T. Henderson, D.

Ranasinghe, V. G. Zakrzewski, J. Gao, N. Rega, G. Zheng, W. Liang, M. Hada, M. Ehara, K. Toyota, R. Fukuda, J. Hasegawa, M. Ishida, T. Nakajima, Y. Honda, O. Kitao, H. Nakai, T. Vreven, K. Throssell, J. A. Montgomery Jr., J. E. Peralta, F. Ogliaro, M. J. Bearpark, J. J. Heyd, E. N. Brothers, K. N. Kudin, V. N. Staroverov, T. A. Keith, R. Kobayashi, J. Normand, K. Raghavachari, A. P. Rendell, J. C. Burant, S. S. Iyengar, J. Tomasi, M. Cossi, J. M. Millam, M. Klene, C. Adamo, R. Cammi, J. W. Ochterski, R. L. Martin, K. Morokuma, O. Farkas, J. B. Foresman and D. J. Fox in *Gaussian 09 Rev. E*, *Vol.* Wallingford, CT, **2009**.

[8] A. D. Becke, *J. Chem. Phys.* **1993**, *98*, 5648-5652.

[9] T. Yanai, D. P. Tew and N. C. Handy, *Chem. Phys. Lett.* **2004**, *393*, 51-57.

[10] S. Grimme, J. Antony, S. Ehrlich and H. Krieg, *J. Chem. Phys.* **2010**, *132*, 154104.

[11] a) T. H. Dunning Jr., *J. Chem. Phys.* **1989**, *90*, 1007-1023; b) R. A. Kendall, T. H. Dunning Jr. and R. J. Harrison, *J. Chem. Phys.* **1992**, *96*, 6796-6806; c) D. E. Woon and T. H. Dunning Jr., *J. Chem. Phys.* **1993**, *98*, 1358-1371.

[12] Y. Y. Chia and M. G. Tay, *Dalton Transactions* **2014**, *43*, 13159-13168.

[13] Y. Zhao and D. G. Truhlar, *Theor. Chem. Acc.* **2008**, *120*, 215-241.

[14] K. J. Cahill, A. Ajaz and R. P. Johnson, *Austr. J. Chem.* **2010**, *63*, 1007-1012.

[15] G. O. Jones and Z. J. Krebs, *Org. Biomol. Chem.* **2017**, *15*, 8326-8333.

[16] C. L. Janssen and I. M. B. Nielsen, *Chem. Phys. Lett.* **1998**, *290*, 423-430.
